# Non-canonical integrin signaling activates EGFR and RAS-MAPK-ERK signaling in small cell lung cancer

**DOI:** 10.7150/thno.79493

**Published:** 2023-04-17

**Authors:** Karla Rubio, Addi J. Romero-Olmedo, Pouya Sarvari, Guruprasadh Swaminathan, Vikas P. Ranvir, Diana G. Rogel-Ayala, Julio Cordero, Stefan Günther, Aditi Mehta, Birgit Bassaly, Peter Braubach, Malgorzata Wygrecka, Stefan Gattenlöhner, Achim Tresch, Thomas Braun, Gergana Dobreva, Miguel N. Rivera, Indrabahadur Singh, Johannes Graumann, Guillermo Barreto

**Affiliations:** 1 Université de Lorraine, CNRS, Laboratoire IMoPA, UMR 7365; F-54000 Nancy, France.; 2 Lung Cancer Epigenetics, Max-Planck-Institute for Heart and Lung Research; 61231 Bad Nauheim, Germany.; 3 Department of Pathology, Massachusetts General Hospital and Harvard Medical School; Charlestown, MA, 02129, USA.; 4 International Laboratory EPIGEN, Consejo de Ciencia y Tecnología del Estado de Puebla (CONCYTEP), Instituto de Ciencias, EcoCampus, Benemérita Universidad Autónoma de Puebla; Puebla 72570, Mexico.; 5 Institute of Medical Microbiology and Hospital Hygiene, Department of Medicine, Philipps-University Marburg; Marburg, Germany.; 6 Independent Researcher, collaborator of International Laboratory EPIGEN-CONCYTEP.; 7 Emmy Noether Research Group Epigenetic Machineries and Cancer, Division of Chronic Inflammation and Cancer, German Cancer Research Center (DKFZ), 69120 Heidelberg, Germany.; 8 Department of Cardiovascular Genomics and Epigenomics, European Center for Angioscience (ECAS), Medical Faculty Mannheim, Heidelberg University, Mannheim, Germany.; 9 German Centre for Cardiovascular Research (DZHK).; 10 ECCPS Bioinformatics and Deep Sequencing Platform, Max-Planck-Institute for Heart and Lung Research; 61231 Bad Nauheim, Germany.; 11 Department of Cardiac Development, Max-Planck-Institute for Heart and Lung Research; 61231 Bad Nauheim, Germany.; 12 Pharmaceutical Technology and Biopharmaceutics, Department of Pharmacy, Ludwig-Maximilians-University of Munich; Munich, Germany.; 13 Institute for Pathology, Justus Liebig University; 35392 Gießen, Germany.; 14 Institute for Pathology, Hannover Medical School; Hanover, Germany.; 15 Biomedical Research in Endstage and Obstructive Lung Disease Hannover (BREATH) Research Network; Hanover, Germany.; 16 Center for Infection and Genomics of the Lung (CIGL), Universities of Giessen and Marburg Lung Center; Giessen, Germany.; 17 Institute of Lung Health, German Center for Lung Research (DZL); Giessen, Germany.; 18 CECAD, University of Cologne; Cologne, Germany.; 19 Faculty of Medicine and University Hospital, University of Cologne; Cologne, Germany.; 20 Center for Data and Simulation Science, University of Cologne; Cologne, Germany.; 21 Center for Cancer Research, Massachusetts General Hospital and Harvard Medical School; Charlestown, MA, 02129, USA.; 22 Biomolecular Mass Spectrometry, Max-Planck-Institute for Heart and Lung Research; 61231 Bad Nauheim, Germany.; 23 Institute of Translational Proteomics, Department of Medicine, Philipps-University Marburg; 35043 Marburg, Germany.

**Keywords:** small cell lung cancer, integrin, EGFR, KRAS, extracellular vesicles

## Abstract

**Background:** Small cell lung cancer (SCLC) is an extremely aggressive cancer type with a patient median survival of 6-12 months. Epidermal growth factor (EGF) signaling plays an important role in triggering SCLC. In addition, growth factor-dependent signals and alpha-, beta-integrin (ITGA, ITGB) heterodimer receptors functionally cooperate and integrate their signaling pathways. However, the precise role of integrins in EGF receptor (EGFR) activation in SCLC remains elusive.

**Methods:** We analyzed human precision-cut lung slices (hPCLS), retrospectively collected human lung tissue samples and cell lines by classical methods of molecular biology and biochemistry. In addition, we performed RNA-sequencing-based transcriptomic analysis in human lung cancer cells and human lung tissue samples, as well as high-resolution mass spectrometric analysis of the protein cargo from extracellular vesicles (EVs) that were isolated from human lung cancer cells.

**Results:** Our results demonstrate that non-canonical ITGB2 signaling activates EGFR and RAS/MAPK/ERK signaling in SCLC. Further, we identified a novel SCLC gene expression signature consisting of 93 transcripts that were induced by ITGB2, which may be used for stratification of SCLC patients and prognosis prediction of LC patients. We also found a cell-cell communication mechanism based on EVs containing ITGB2, which were secreted by SCLC cells and induced in control human lung tissue RAS/MAPK/ERK signaling and SCLC markers.

**Conclusions:** We uncovered a mechanism of ITGB2-mediated EGFR activation in SCLC that explains EGFR-inhibitor resistance independently of EGFR mutations, suggesting the development of therapies targeting ITGB2 for patients with this extremely aggressive lung cancer type.

## Introduction

Lung cancer (LC) causes more deaths worldwide than the next three most prevalent cancers together (colorectal, breast and prostate) 1. Based on histology, LC is classified into non-small cell (NSCLC) and small cell lung cancer (SCLC). NSCLC can be further classified into three subtypes: squamous cell carcinoma, adenocarcinoma, and large-cell lung cancer 2. SCLC accounts for 15-20% of all LC cases and is strongly associated with cigarette smoking 3. SCLC is a neuroendocrine type of lung cancer characterized by aggressive progression due to high cellular proliferation and early metastasis 4. The first line of therapy includes a combination of platinum-based treatment (cisplatin or carboplatin) with etoposide or irinotecan. While SCLC is initially chemo- and radiosensitive, therapy resistance frequently arises. Consequently, patient prognosis is poor with a median survival of 6 to 12 months 5.

Extracellular vesicles (EVs) are nano-sized, phospholipid membrane-enclosed vesicles that are secreted by different cell types into the extracellular space and can be found in a wide spectrum of human body fluids including serum, plasma, saliva, breast milk, amniotic fluid, cerebrospinal fluid, urine, among others 6, 7. EVs are classified based on their size, biogenesis and method of cellular release into three groups: exosomes, microvesicles and apoptotic bodies. Microvesicles and apoptotic bodies are formed by budding from the plasma membrane, and generally range in size from 0.1 to 1 μm and 1 to 4 μm, respectively 8, 9. In contrast, exosomes are smaller with a diameter ranging from 30 to 150 nm, and are formed by inward budding of the endosome lumen to form a multivesicular body, which fuses with the plasma membrane during secretion 10. Due to an overlap in size (100-150 nm) and density (1.08-1.19 g/mL), as well as the presence of common markers, such as CD63 and CD81 11, it is difficult to differentiate exosomes and microvesicles. Thus, exosomes and microvesicles with a diameter below 150 nm are collectively referred to as small extracellular vesicles (small EVs) 12. EV secretion is elevated in response to inflammation 13 and hypoxia 14, 15 and is associated with human diseases. In cancer, for example, EV levels correlate with tumor invasiveness 16-18. Interestingly, EVs contain proteins and nucleic acids, and it has been reported that tumor cells can influence their microenvironment through EV-based cell-cell communication mechanisms 19-23.

Epidermal growth factor (EGF) signaling plays an important role in LC development and metastasis. In particular, activation of EGF receptor (EGFR) tyrosine kinases (RTK) is crucial for triggering SCLC and NSCLC 24. EGFR is composed of an extracellular ligand-binding domain, a transmembrane domain and an intracellular tyrosine kinase domain. The binding of a ligand to the extracellular domain of EGFR induces receptor dimerization, activation of the intracellular kinase domain and auto-phosphorylation of tyrosine residues within the cytoplasmic domain of the receptor 25. The tyrosine-phosphorylated motifs of EGFR recruit adapters or signaling molecules that initiate various downstream signaling cascades including the RAS/MAPK/ERK, PIK3-Akt and STAT pathways 26. These signaling cascades result in transcriptional activation of gene signatures that mediate specific cellular responses, such as cell proliferation, cell migration, epithelial-mesenchymal transition (EMT), among others.

Integrins are heterodimeric transmembrane protein complexes resulting from noncovalent association of specific alpha (ITGA) and a beta (ITGB) subunits 27. In general, each integrin subunit has a large extracellular domain, a single-pass transmembrane domain, and a short cytoplasmic tail 27. Integrins mediate cell-cell and cell-ECM (extracellular matrix) interactions and transmit signals in both directions, outside-in and inside-out, across the cell membrane 28. Recent studies have shown that growth factor- and integrin-dependent signals functionally cooperate to integrate their signaling pathways 29, 30. The crosstalk between EGFR and integrins has been reported to play a key role in multiple biological processes in cancer 31. Moreover, several integrin dimers, including ITGA5-ITGB1, ITGA6-ITGB4, ITGAv-ITGB3 and ITGAv-ITGB5, exert different effects on the regulation of EGF signaling 32-34. We have previously demonstrated that ITGA2, ITGB2 and ITGB6 are enriched in the membrane of alveolar type-II (ATII) cells, which are lung progenitor cells responsible for regeneration of the alveolar epithelium during homeostatic turnover and in response to injury 35. In addition, ATII cells were also reported as cells of origin of lung adenocarcinoma 36. These observations motivated us to investigate the function of integrin receptor subunits in LC.

## Results

### Non-canonical ITGB2 signaling activates EGFR in SCLC

**Figure 1:**
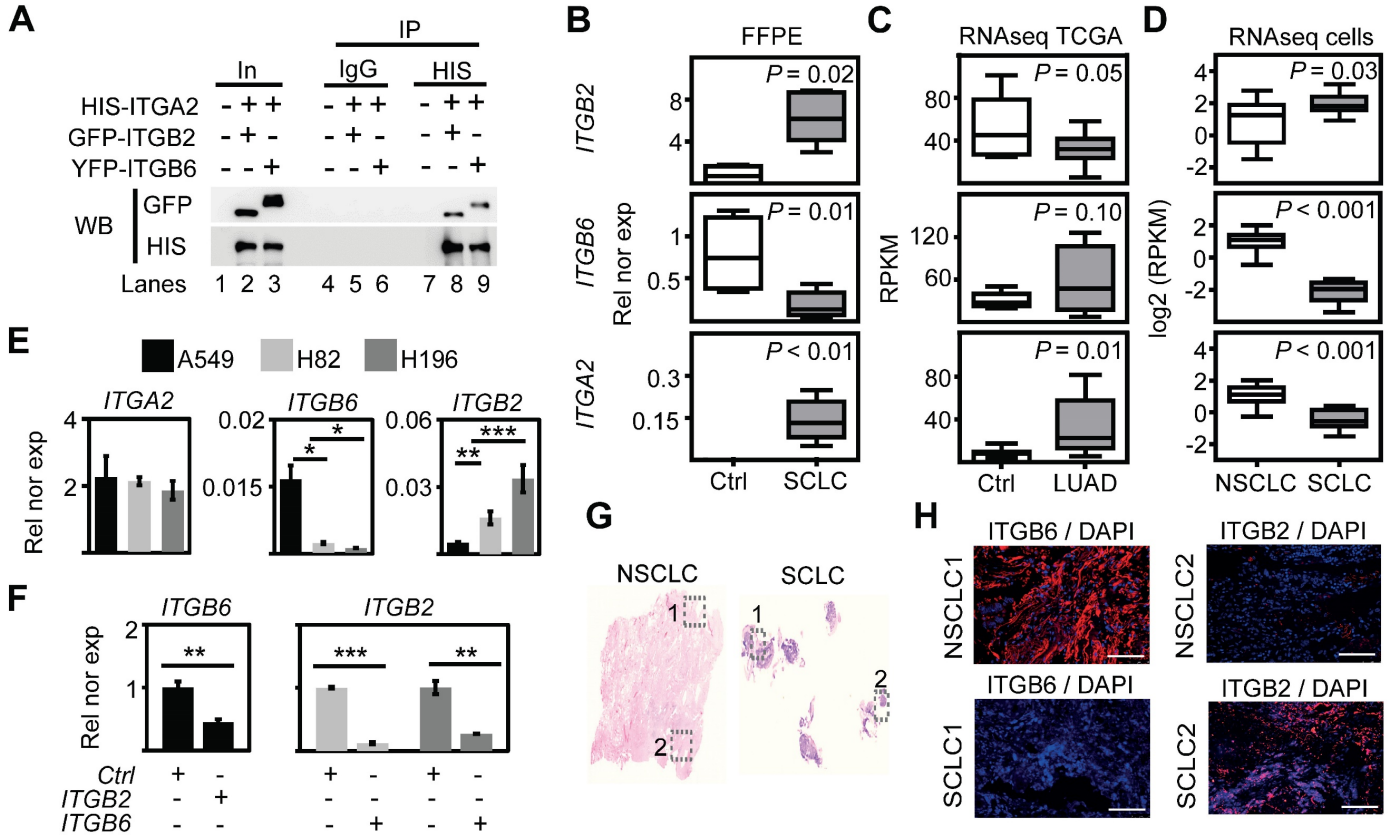
** Mutual negative regulation of ITGB2 and ITGB6 levels in SCLC and NSCLC.** (**A**) Protein extracts of MLE-12 cells co-transfected with *ITGA2*-*HIS* and *ITGB2*-*YFP* or *ITGA2*-*HIS* and *ITGB6*-*GFP* were immunoprecipitated (IP) using either immunoglobulin G (IgG, as control) or HIS-specific antibodies. Co-IP proteins were analyzed by WB using the indicated antibodies. In, input, 3% of material used for the IP. (**B**) Box plots of qRT-PCR-based expression analysis of indicated transcripts using RNA isolated from FFPE lung tissue sections from Ctrl (*n* = 4) and small cell-lung cancer (SCLC, *n* = 5) patients. Rel nor exp, relative normalized expression to *GAPDH*. (**C**) Box plots of RNA-seq-based expression analysis of indicated transcripts in matched control donors (Ctrl; *n* = 9) and matched lung adenocarcinoma (LUAD; *n* = 11) patients from the Cancer Genome Atlas (TCGA). Values were normalized using reads per kilobase per million (RPKM). (**D**) Box plots of RNA-seq-based expression analysis of indicated transcripts in non-small cell lung cancer (NSCLC; *n* = 34) and small cell lung cancer (SCLC; *n* = 17) cell lines. Values are represented as log2 RPKM. All box plots (B-D) indicate median (middle line), 25th, 75th percentile (box) and 5th and 95th percentile (whiskers); *P*-values after two-tailed t-test. (**E**) qRT-PCR-based expression analysis of indicated mRNA in A549, NCI-H82 and NCI-H196 cell lines. (**F**) qRT-PCR-based expression analysis of indicated mRNA in A549 cells (left panel) transfected with *ITGB2* or NCI-H196 cells (right panel) transfected with *ITGB6*. In the bar plots (E-F), data are shown as means ± SD (*n* = 3); asterisks, *P*-values after two-tailed t-test, *** *P* ˂ 0.001; ** *P* ˂ 0.01; * *P* ˂ 0.05. (**G**) Hematoxylin and eosin staining in human lung tissue from NSCLC (left) and SCLC (right) patients. Squares are respectively shown in E at higher magnification. (**H**) Fluorescence microscopy after immunostaining using ITGB6 or ITGB2-specific antibodies in NSCLC and SCLC FFPE lung tissues (in G). DAPI, nuclear staining. Scale bar, 500 µm. Source data for all plots are provided as Source Data S1. See also Figure S1-S5 and Source Data S2.

The previously reported enrichment of ITGA2, ITGB2 and ITGB6 in the membrane of lung progenitor cells 35 suggests a potential interaction between these integrin receptor subunits. To test this hypothesis, we performed *in silico* analyses of proteomic data repositories derived from protein-protein interaction databases (Figure S1A). Using the STRING database, we identified 50 interaction partners of human ITGA2 with high confidence (combined score ≥ 0.9; 2 nodes, Table S1), including ITGB2 (combined score = 0.96) and ITGB6 (combined score = 0.97). To confirm the interaction of ITGA2 with ITGB2 and ITGB6, we performed co-immunoprecipitation (Co-IP) assays using total protein extracts from mouse lung epithelial cells (MLE-12) transiently transfected with *HIS-ITGA2* and *YFP*-*ITGB2* or *GFP-ITGB6* (Figure 1A). ITGA2 precipitated both ITGB2 and ITGB6, thus validating our *in silico* analysis and demonstrating the interaction between these integrin receptor subunits. To assess whether *ITGB2*, *ITGB6* and *ITGA2* are expressed in SCLC, we performed qRT-PCR-based expression analysis using total RNA extracted from retrospectively collected formalin-fixed paraffin embedded (FFPE) human lung tissues from SCLC patients (*n* = 5) and control donors (Ctrl, *n* = 4; Table S2 and Figure 1B). We observed increased *ITGB2* (*P* = 0.02) and *ITGA2* (*P* = 9 E-03) expression in SCLC as compared to Ctrl FFPE human lung tissue, whereas *ITGB6* levels were reduced (*P* = 0.01). To perform an equivalent analysis in NSCLC, we retrieved RNA-sequencing (RNA-seq) data of lung adenocarcinoma patients (LUAD, *n* = 11) and control donors (Ctrl, *n* = 9) from The Cancer Genome Atlas (TCGA) (Table S3). In contrast to SCLC, we observed decreased *ITGB2* (*P* = 0.05) in LUAD as compared to Ctrl human lung tissue (Figure 1C). Similarly to SCLC, *ITGA2* expression also increased (*P* = 0.01) in LUAD compared to Ctrl. Consistent with these results, we found a positive correlation between the expression of *ITGB2* and *ITGA2* in SCLC (R^2^ = 0.84, *P* < 0.05, Figure S1B, top) and a positive correlation between the expression of *ITGB6* and *ITGA2* in LUAD (R^2^ = 0.94, *P* < 1 E-04, Figure S1B, bottom) by linear regression analysis. Our results demonstrated the complementary expression of *ITGB2* and *ITGB6* in SCLC and LUAD suggesting their use as markers for these cancer subtypes and supporting the formation of different integrin heterodimer receptors in SCLC and LUAD.

**Figure 2:**
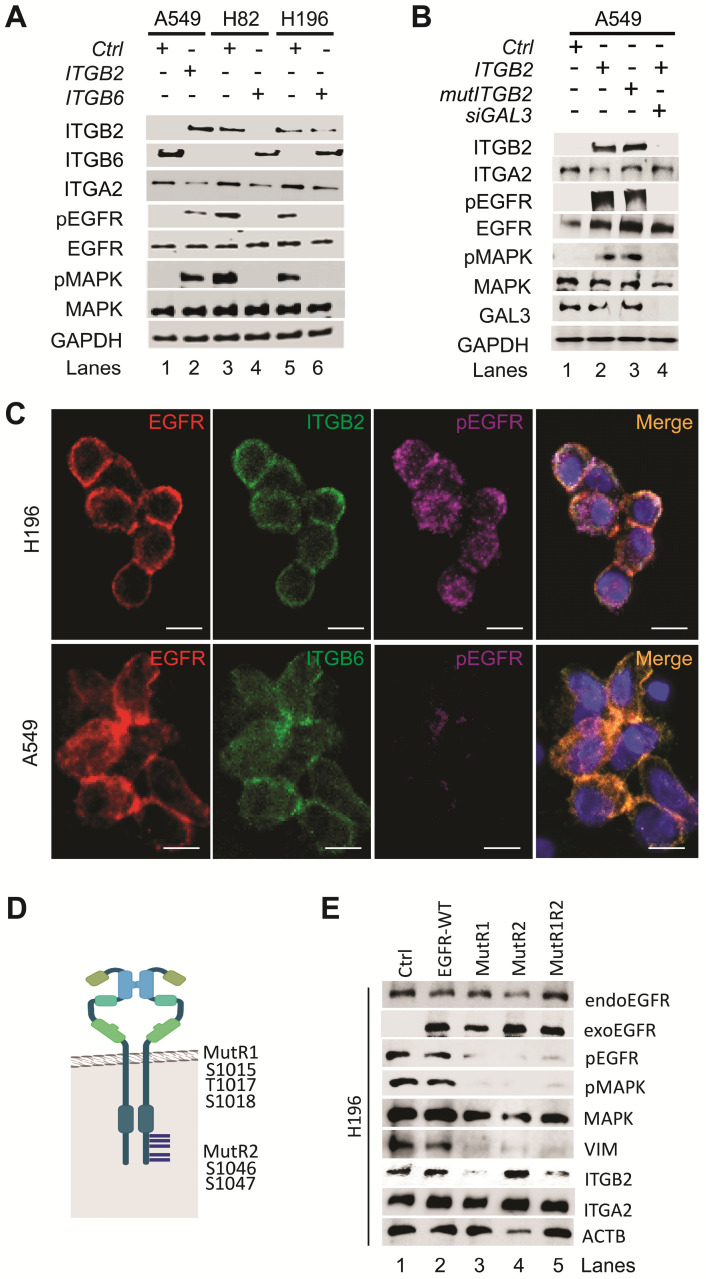
** Non-canonical ITGB2 signaling activates EGFR in SCLC.** (**A**) Total protein extracts of A549, NCI-H82 and NCI-H196 cell lines transfected with *ITGB2* or *ITGB6* were analyzed by WB using the indicated antibodies. (**B**) Total protein extracts of A549 cells transfected with *ITGB2,* ligand-binding-deficient D134A ITGB2 mutant (*mutITGB2*) or Galectin-3-specific small interfering RNA (*siGAL3*) were analyzed by WB using the indicated antibodies. (**C**) Confocal microscopy after immunostaining with specific antibodies against EGFR, pEGFR, ITGB2 and ITGB6 in NCI-H196 and A549 cells. DAPI, nucleus. Scale bars, 10 μm. (**D**) Schematic representation of a EGFR-dimer highlighting specific key amino acid residues, including phosphorylation sites in two p38 target regions (R1 and R2; 39) that were mutated in two expression constructs MutR1 and MutR2. (**E**) Total protein extracts of NCI-H196 cell lines were analyzed by WB using the indicated antibodies. NCI-H196 cells were transiently transfected with control empty plasmid (Ctrl), or constructs for expression of MYC-tagged wild-type EGFR (*EGFR-WT*) or EGFR mutants, in which either S1015, T1017 and S1018 were mutated to alanine (*MutR1*); or S1046 and S1047 were mutated to alanine (*MutR2*) alone or in combination (*MutR1R2*). Exogenous EGFR (exoEGFR) was differentiated from endogenous EGFR (endoEGFR) using MYC-tag-specific antibodies. See also Figure S1-S5 and Source Data S2.

To further investigate the differential expression of *ITGB6* and *ITGB2* in lung cancer subtypes, we analyzed publicly available RNA-seq data of NSCLC and SCLC cell lines (Figure S2) 37. Consistent with our results in human lung tissue (Figure 1B-C), we detected high levels of *ITGB2* in SCLC cell lines (Figure 1D, top), whereas *ITGB6* levels were high in NSCLC cell lines (Figure 1D, middle). In addition, linear regression analysis confirmed the positive correlation between *ITGB2* and *ITGA2* levels in SCLC cell lines (R^2^ = 0.82; *P* = 4 E-03; Figure S1C, top) and the positive correlation between *ITGB6* and *ITGA2* levels in NSCLC cell lines (R^2^ = 0.72; *P* = 9 E-04; Figure S1C, bottom). To further investigate these findings, we selected the human adenocarcinoma cell line A549 as a NSCLC-representative cell line, whereas the human cell lines NCI-H82 and NCI-H196 were selected as representative cells for SCLC. The selected cell lines lack somatic mutations in the *EGFR* locus (Figure S3) and are experimental systems commonly used in LC subtype-specific studies. Expression analysis by qRT-PCR (Figure 1E-F) confirmed the results obtained by RNA-seq (Figure 1D), high *ITGB2* levels were detected in SCLC cell lines (*P* = 0.005 and *P* = 0.001), whereas A549 cells showed high *ITGB6* levels (*P* = 0.023 and *P* = 0.019). Interestingly, transient transfection of *ITGB2* in A549 cells significantly reduced *ITGB6* expression (*P* = 0.0171; Figure 1F, left), whereas *ITGB6* transfection in NCI-H82 and NCI-H196 cells significantly reduced *ITGB2* expression (*P* = 0.0002 and *P* = 0.0098, respectively; Figure 1F, right). Moreover, our qRT-PCR-based expression analyses were also confirmed in FFPE of lung tissue obtained from NSCLC and SCLC patients stained with ITGB6- or ITGB2-specific antibodies (Figure 1G-H) and by Western Blot analysis (WB) of protein extracts from transfected A549, NCI-H82 and NCI-H196 cells (Figure 2A, top). Our results support a mutual negative regulation of *ITGB6* and *ITGB2* expression in NSCLC and SCLC.

Additional analysis of the RNA-seq data from NCI-H82 and NCI-H196 cells showed enrichment of pathways related to EGFR signaling (Figure S4A-B). We further observed increased levels of key downstream genes of EGFR signaling, such as *VIM*, *NFKB2* and *HIF1A*, in RNA-seq data from SCLC cell lines as compared to NSCLC cell lines (Figure S4C-D) 37, which were confirmed by qRT-PCR-based expression analysis in FFPE human lung tissue (Figure S4E). Our results indicate that EGFR signaling is active in SCLC and correlates with increased *ITGB2* expression. To further investigate this finding, we analyzed protein extracts from transiently transfected A549, NCI-H82 and NCI-H196 cells by WB (Figure 2A, middle). Overexpression of *ITGB2* in A549 cells induced phosphorylation of EGFR (pEGFR) and the mitogen-activated protein kinase (pMAPK) as compared to *Ctrl* transfected cells. On the other hand, the levels of pEGFR and pMAPK in NCI-H82 and NCI-H196 cells were reduced after *ITGB6* transfection. Moreover, the changes in pEGFR and pMAPK in all three cell lines did not affect total levels of EGFR and MAPK, thereby indicating that the observed effects were related to the post-translational phosphorylation of these proteins. To gain further insight into the mechanism of ITGB2-induced, phosphorylation-dependent activation of EGFR and MAPK, we investigated the involvement of non-canonical, ligand-independent integrin signaling 33, 38. We generated a ITGB2 mutant (*mutITGB2*) that is ligand-binding-deficient, as aspartic acid 134 in the ligand-binding domain was substituted by alanine (Figure S5A). Overexpression of *mutITGB2* in A549 cells (Figure 2B and S5B) induced pEGFR and pMAPK, as well as increased the levels of VIM and ACTA2 in Galectin-3 (GAL3) -dependent manner, demonstrating the involvement of non-canonical, ligand-independent integrin signaling during the phosphorylation-dependent activation of EGFR and MAPK. In addition, we observed co-localization of ITGB2 and pEGFR in NCI-H196 cells (Figure 2C, top), whereas ITGB6 and EGFR co-localized in A549 cells (Figure 2C, bottom). To further characterize the phosphorylation-dependent activation of EGFR during non-canonical, ligand-independent integrin signaling in SCLC, we analyzed protein extracts from transiently transfected NCI-H196 cells by WB (Figure 2D-E). Overexpression of an EGFR mutant (MutR1, in which serine 1015, threonine 1017 and serine 1018 were substituted by alanine) 39 acted as a dominant-negative EGFR abolishing pEGFR and pMAPK and reducing VIM and ITGB2 levels. Interestingly, overexpression of a second EGFR mutant (MutR2, in which serines 1046 and 1047 were mutated to alanine) also interfered with the phosphorylation-dependent activation of EGFR and MAPK and reduced VIM levels without affecting ITGB2 levels. In summary, our results demonstrate that non-canonical ITGB2 signaling activates EGFR in SCLC (Figure 2B). In addition, whereas both EGFR mutants (MutR1 and MutR2) acted as dominant-negative forms interfering with phosphorylation-dependent activation of EGFR and MAPK (Figure 2D-E), only MutR1 reduced ITGB2 levels suggesting specificity in the role of the phosphorylation of the mutated amino acids during non-canonical, ligand-independent integrin signaling in SCLC.

### ITGB2 induces a novel SCLC gene expression signature

**Figure 3:**
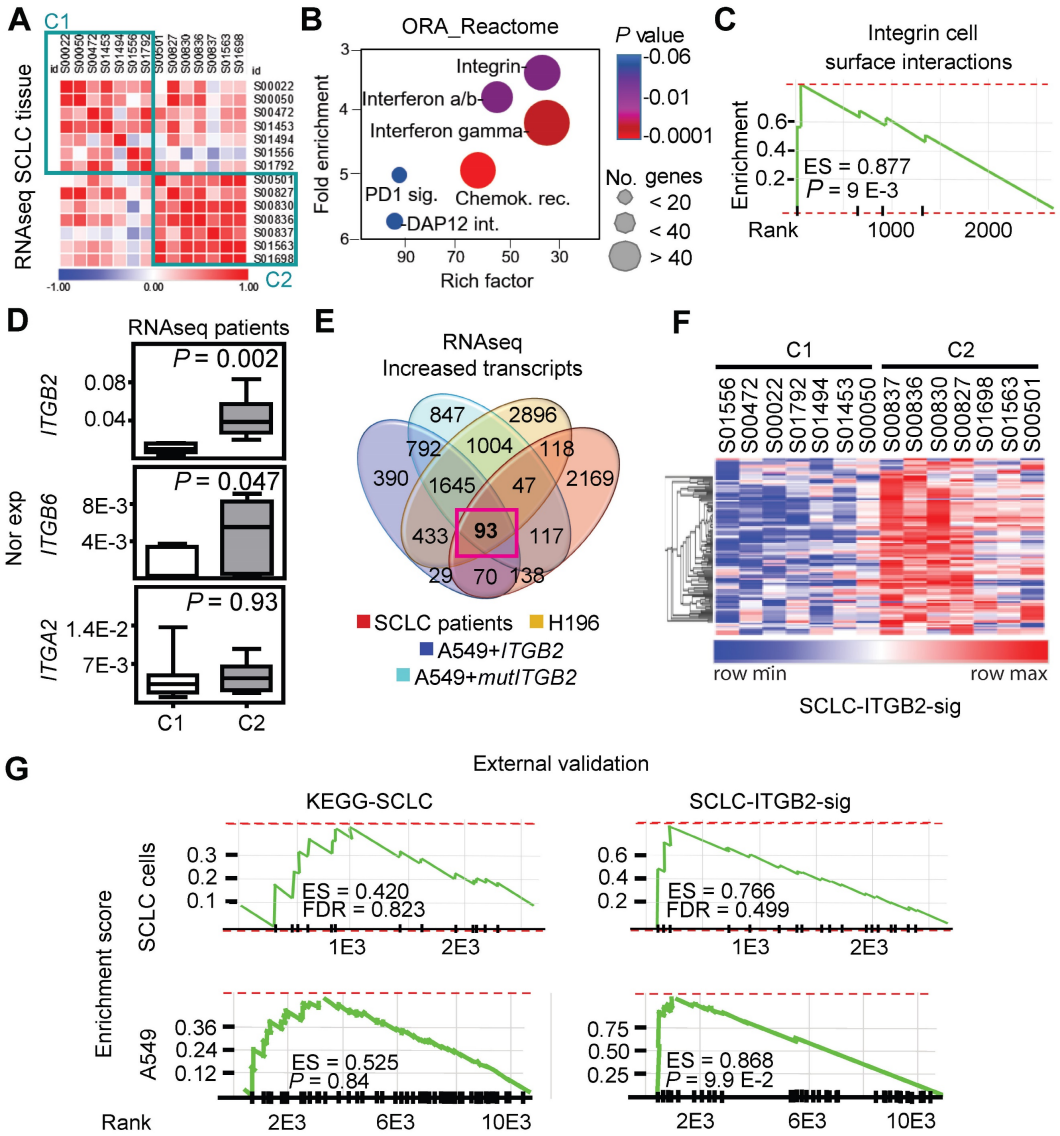
** ITGB2 induces a novel SCLC gene expression signature.** (**A**) Correlogram showing the correlation score matrix (RPKM values, Spearman correlation coefficient) across RNA-seq data of lung tissue of SCLC patients from the European Genome Archive (EGAS00001000299). SCLC patients were grouped in cluster 1 (C1) and cluster 2 (C2). (**B**) Bubble plot of top six enrichment of Reactome pathways in C2 by Overrepresentation Analysis (ORA). *P*-values after two-tailed t-test are shown by different color, the size of bubble indicate the gene count of each pathway. Sig., signaling; int., interactions; chemok., chemokine; rec., receptor. (**C**) Gene Set Enrichment Analysis (GSEA) using the fold change of genes inside the integrin pathway in B. ES, enrichment score; *P-*value after two-tailed t-test. (**D**) Box plots of RNA-seq-based expression analysis of indicated transcripts in SCLC patients C1 (*n* = 7) and C2 (*n* = 7). Values are represented as log2 RPKM. Nor exp, normalized expression to *GAPDH*. Box plots indicate median (middle line), 25th, 75th percentile (box) and 5th and 95th percentile (whiskers); *P*-values after two-tailed t-test. Source data are provided as Source Data S1. (**E**) Venn diagram comparing transcripts that were significantly increased in SCLC patients C2 compared to C1 (FC ≥ 3; *P* ≤ 0.05 after two-tailed t-test), NCI-H196 cells, A549 cells transfected either with *ITGB2* or *mutITGB2* (for all 3 cell lines, coding transcripts; FC ≥ 1.15; *P* ≤ 0.05 after two-tailed t-test) highlights a group of 93 transcripts that are common in all four groups, the SCLC-ITGB2 gene expression signature (SCLC-ITGB2-sig). See also Table S4. (**F**) Hierarchical heatmap using RPKM of all 93 IDs of the SCLC-ITGB2-sig comparing SCLC patients in C1 to C2. Hierarchical clustering was performed using Person's correlation based distance and average linkage. (**G**) External validation of the SCLC-ITGB2-sig. GSEA using independent RNA-seq data from SCLC cell lines 42 comparing the conventional SCLC signature in KEGG (left) versus the SCLC-ITGB2-sig (right) identified in E. ES, enrichment score; FDR, false discovery rate. See also Figure S6 and S7.

We retrieved and analyzed RNA-seq data of a previously published cohort of SCLC patients from the European Genome-Phenome Archive (Figure S6) 40. Correlation analysis of these RNA-seq based transcriptomes allowed us to group the SCLC patients into two clusters (C1 and C2; Figure 3A). Over-representation analysis (ORA) based on the Reactome database 41 for genes with increased expression in C2 (Figure 3B; 5,149 transcripts with fold change (FC) ≥ 3; Source Data S1) revealed a significant enrichment of genes related to the category “integrin cell surface interactions” (*P* = 9.1 E-03) as one of the top items of the ranked list. In addition, gene set enrichment analysis (GSEA) of the up-regulated transcripts in C2 (Figure 3C) showed a high enrichment score (ES) of 0.88 for the category “integrin cell surface interactions” (*P* = 9 E-03). Interestingly, lung tissue from SCLC patients in C2 showed significantly higher *ITGB2* expression (M = 0.04; IQR = 0.03) than the lung tissue from SCLC patients in C1 (M = 0.01; IQR = 0.01; *P* = 2 E-03; Figure 3D; Source Data S1). Moreover, we identified 93 transcripts that were jointly upregulated in C2, in the SCLC cell line NCI-H196, as well as in A549 cells transiently transfected with *ITGB2* or *mutITGB2* (Figure 3E; Table S4). We named the set of 93 genes coding for these common upregulated transcripts as SCLC-ITGB2 gene expression signature (SCLC-ITGB2-sig). As shown in the heatmap in Figure 3F, the expression levels of the 93 genes, constituting the SCLC-ITGB2-sig, differentiate the two clusters of SCLC patients. Comparing the enrichment of the genes grouped into the Kyoto Encyclopedia of Genes and Genomes (KEGG) category “SCLC” and the SCLC-ITGB2-sig (Figure S7), we observed ES of 0.52 (*P* = 0.37) and 0.52 (*P* = 0.36) for the KEGG-SCLC set in SCLC patients of C2 and C1, respectively, whereas the ES of the SCLC-ITGB2-sig significantly improved to 0.97 (*P* = 8 E-03) and 0.98 (*P* = 8 E-03) in SCLC patients of C2 and C1, respectively. Moreover, an external validation (Figure 3G) using independent RNA-seq data from SCLC cell lines 42 confirmed the improvement of the SCLC-ITGB2-sig (ES = 0.77; FDR = 0.50) as compared to the KEGG-SCLC category (ES = 0.42; FDR = 0.82). Thus, we propose the use of SCLC-ITGB2-sig for the identification of gene expression signatures related to SCLC.

**Figure 4:**
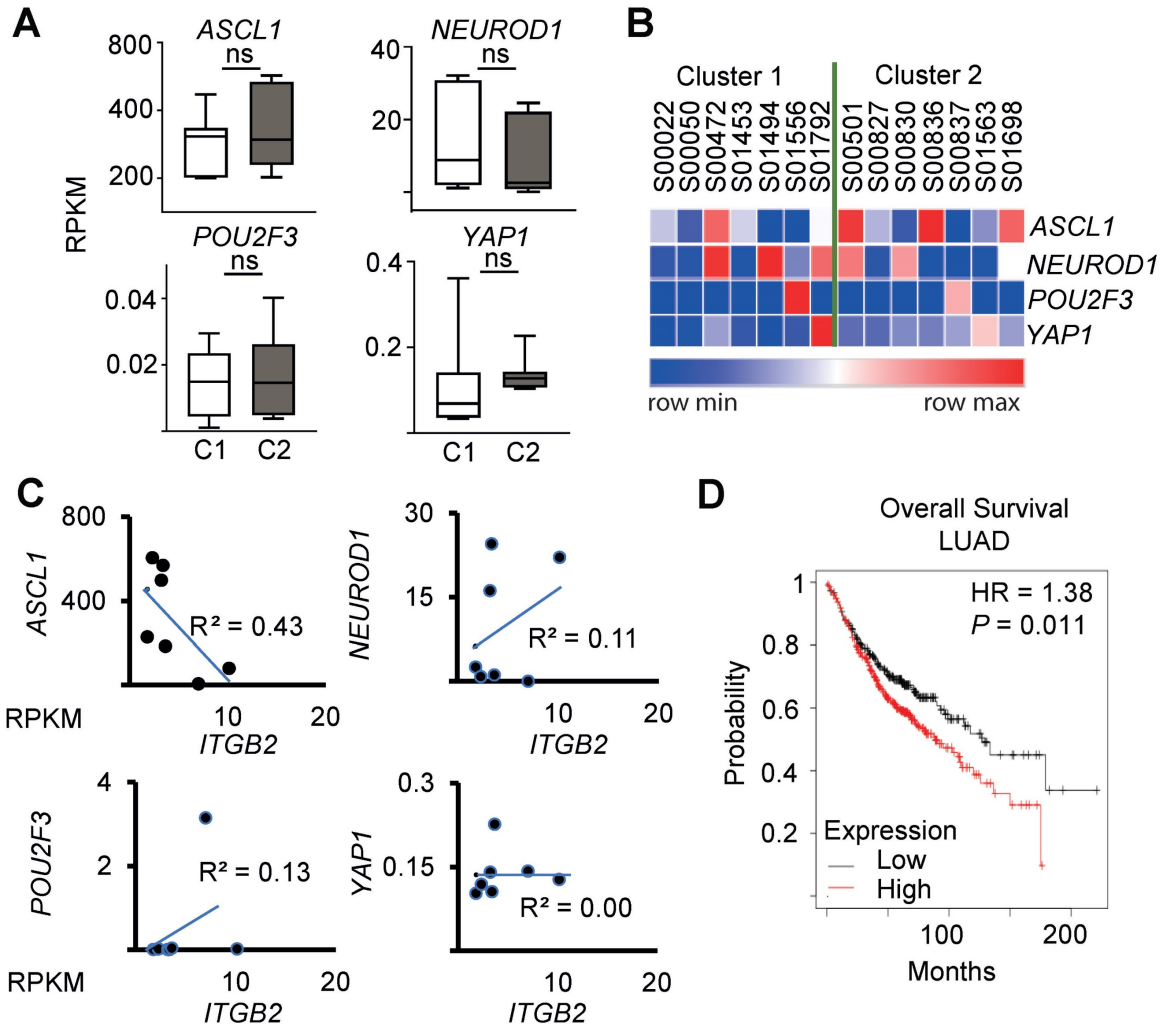
** SCLC-ITGB2 gene expression signature occurs in all SCLC subtypes.** (**A**) Box plots of RNA-seq-based expression analysis of the transcripts used for SCLC subtype classification (*ASCL1*, *NEUROD1*, *POU2F3* and *YAP1*) 43, 44 in SCLC patients C1 (*n* = 7) and C2 (*n* = 7). Values are represented as log2 RPKM. Nor exp, normalized expression to *GAPDH*. Box plots indicate median (middle line), 25th, 75th percentile (box) and 5th and 95th percentile (whiskers); *P*-values after two-tailed t-test. Source data are provided as Source Data S1. (**B**) Heat map representing the RNA-seq-based expression levels of the indicated transcripts in SCLC patients C1 (*n* = 7) and C2 (*n* = 7). Blue, low expression; red, high expression. (**C**) Correlation analysis between *ITGB2* and *ASCL1*, *NEUROD1*, *POU2F3* and *YAP1* by linear regression of relative normalized expression from RNA-seq-based expression analysis of indicated transcripts in SCLC patients C1 (*n* = 7) and C2 (*n* = 7). (**D**) Overall survival rates by Kaplan-Meier plotter of LUAD patients expressing low (*n* = 300) or high (*n* = 372) SCLC-ITGB2-sig (127 vs 88.7 months, respectively, *P* = 0.011 after after Log Rank test). HR, hazard ratio. See also Figure S6 and S7.

Recent reports suggest a SCLC classification in subtypes based on the expression of *ASCL1*, *NEUROD1*, *POU2F3* and *YAP1* 43, 44. Interestingly, we did not detect significant differences in the expression levels of these markers between the clusters C1 and C2 of SCLC patients (Figure 4A-B), nor a significant linear correlation between the expression of *ITGB2* and *ASCL1* (R^2^ = 0.43), *NEUROD1* (R^2^ = 0.11), *POU2F3* (R^2^ = 0.13) and *YAP1* (R^2^ = 0.00) by linear regression analysis (Figure 4C). These results suggest that the SCLC-ITGB2-sig arises in all four SCLC subtypes. Further, a survival analysis of 673 LC patients from Kaplan-Meier plotter 45 (Figure 4D) showed a significantly shorter survival of patients with increased levels of these 93 transcripts (*n =* 372) compared to patients with low expression levels (*n* = 300). The median survival in the two groups were 88.7 and 127 months, respectively (hazard ratio = 1.38, *P* = 0.01, Cox regression). These findings support the clinical relevance of the SCLC-ITGB2-sig not only for stratification of SCLC patients, which might help to develop patient-tailored therapies, but also for prognosis prediction of LC patients.

### Non-canonical ITGB2 signaling activates RAS/MAPK/ERK signaling

**Figure 5:**
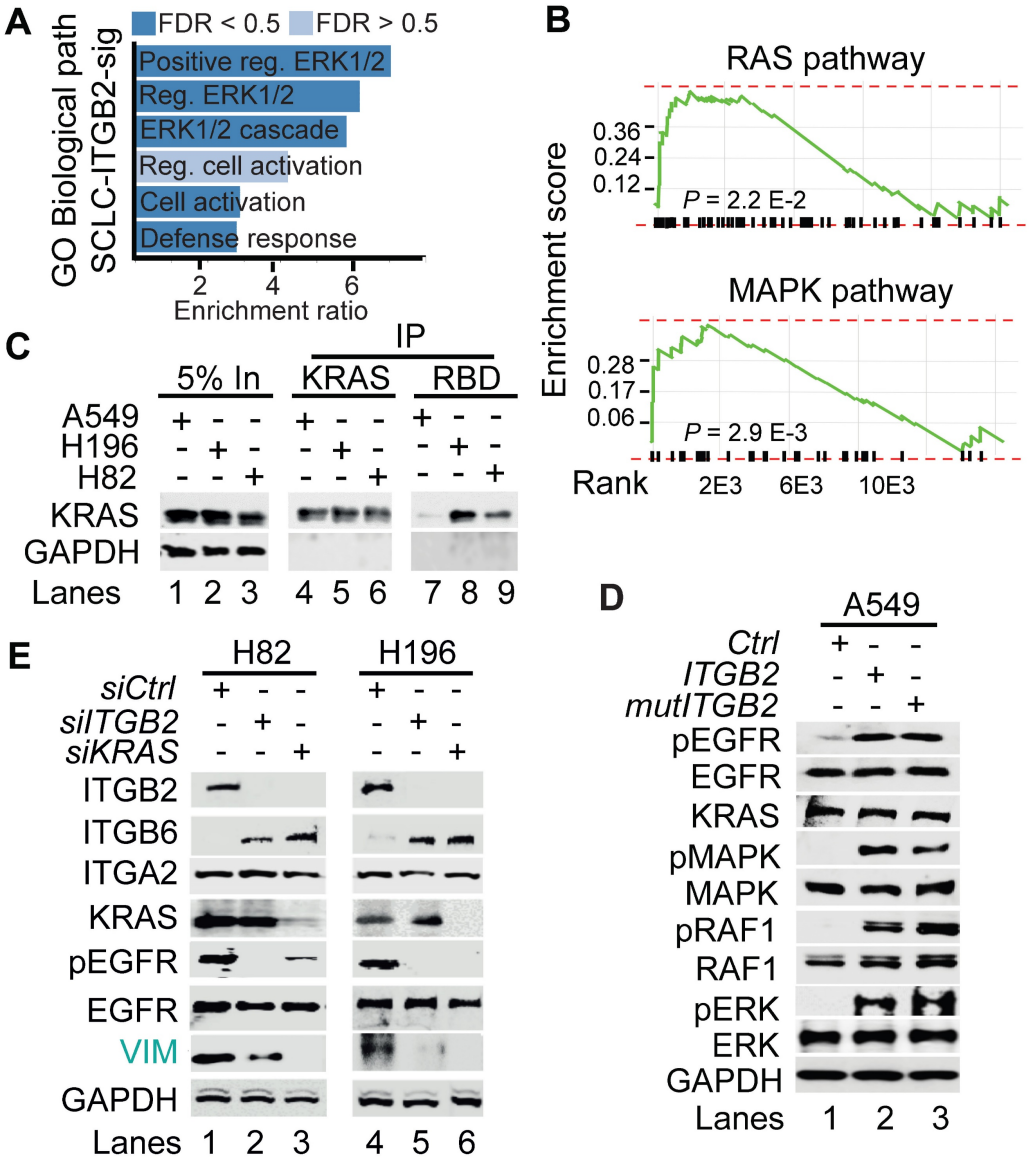
** Non-canonical ITGB2 signaling activates RAS/MAPK/ERK signaling.** (**A**) Gene Ontology (GO)-based enrichment analysis of biological pathways in the 93 IDs of the SCLC-ITGB2 gene expression signature (SCLC-ITGB2-sig) from Figure 3E using Webgestalt bioinformatics tool and plotted by highest enrichment ratio. Reg., regulation. (**B**) GSEA line profiles of SCLC-ITGB2-sig in RAS (Panther) and MAPK signaling pathways (KEGG). *P*-values after two-tailed t-test. (**C**) RAS activation assay. Protein extracts of A549, NCI-H196 and NCI-H82 were immunoprecipitated (IP) using a KRAS-specific antibody (KRAS) or RAF-RBD (RBD, active KRAS) coated beads. Co-IP proteins were analyzed by WB using the indicated antibodies. In, input, 5% of material used for the IP. (**D**) Total protein extracts of A549 cells transfected with *ITGB2* or ligand-binding-deficient D134A ITGB2 mutant (*mutITGB2*) were analyzed by WB using the indicated antibodies. pEGFR, active phosphorylated epidermal growth factor receptor; pMAPK, phosphorylated mitogen-activated protein kinase; pRAF1, phosphorylated proto-oncogene serine/threonine-protein kinase; pERK, phosphorylated extracellular signal-regulated kinase. (**E**) Total protein extracts of NCI-H82 and NCI-H196 cells transfected with small interfering RNA specific for *ITGB2* (*siITGB2*) or *KRAS* (*siKRAS*) were analyzed by WB using the indicated antibodies. Vimentin (VIM) as product of a downstream gene target of EGF signaling is highlighted in green. See also Source Data S2.

To further characterize the SCLC-ITGB2-sig, we performed a gene ontology (GO) enrichment analysis based on Biological Processes and found significant enrichment of genes related to GO terms involved in extracellular signal-regulated kinase 1/2 (ERK1/2) signaling pathway (Figure 5A). In addition, we also detected significant enrichment of genes related to RAS pathway (*P* = 2.2 E-02; FDR = 0.26) and MAPK pathway (*P* = 2.9 E-03; FDR = 0.1) (Figure 5B). These findings corroborated our previous results (Figure 2A-C and S4-S5), as Ras family proteins are known to be activated by signaling through EGFR 46.

To further investigate the involvement of active RAS/MAPK/ERK signaling in SCLC, we performed RAS activation assay using whole cell protein extracts from A549, NCI-H196 and NCI-H82 cells (Figure 5C). This assay is based on the principle that the RAS binding domain (RBD) of the RAF kinase, one of the downstream RAS effector proteins, binds specifically to the GTP-bound form of RAS 47. Using RAF-RBD coated beads, we pulled down active, GTP-bound KRAS from protein extracts of NCI-H196 and NCI-H82 cells, supporting that RAS/MAPK/ERK signaling is active in SCLC. Interestingly, transfection of *ITGB2* or *mutITGB2* into A549 cells (Figure 5D) induced phosphorylation of EGFR, MAPK, RAF1 and ERK without significantly affecting the total levels of these proteins, thereby showing that non-canonical ITGB2 activates the EGFR and the RAS/MAPK/ERK signaling pathway. Further, we determined whether ITGB2 and KRAS are required for the intrinsic levels of pEGFR observed in both SCLC cell lines NCI-H82 and NCI-H196 (Figure 2A). We analyzed by WB protein extracts from NCI-H82 and NCI-H196 cells that were transfected with Ctrl, *ITGB2*- or *KRAS*-specific small interfering RNAs (siRNA,* siCtrl*, *siITGB2* or *siKRAS*; Figure 5E). Specific and efficient siRNA-mediated *ITGB2* or *KRAS* loss-of-function (LOF) reduced the levels of pEGFR and the downstream target of EGFR signaling VIM, demonstrating the requirement of ITGB2 and KRAS for the activation of EGFR and the RAS/MAPK/ERK signaling in SCLC. Interestingly, *siITGB2* or *siKRAS* transfection increased ITGB6 levels in both SCLC cell lines, supporting the mutual exclusive function of ITGB2 and ITGB6 shown in Figure 1F, 2A and S5B-C.

**Figure 6:**
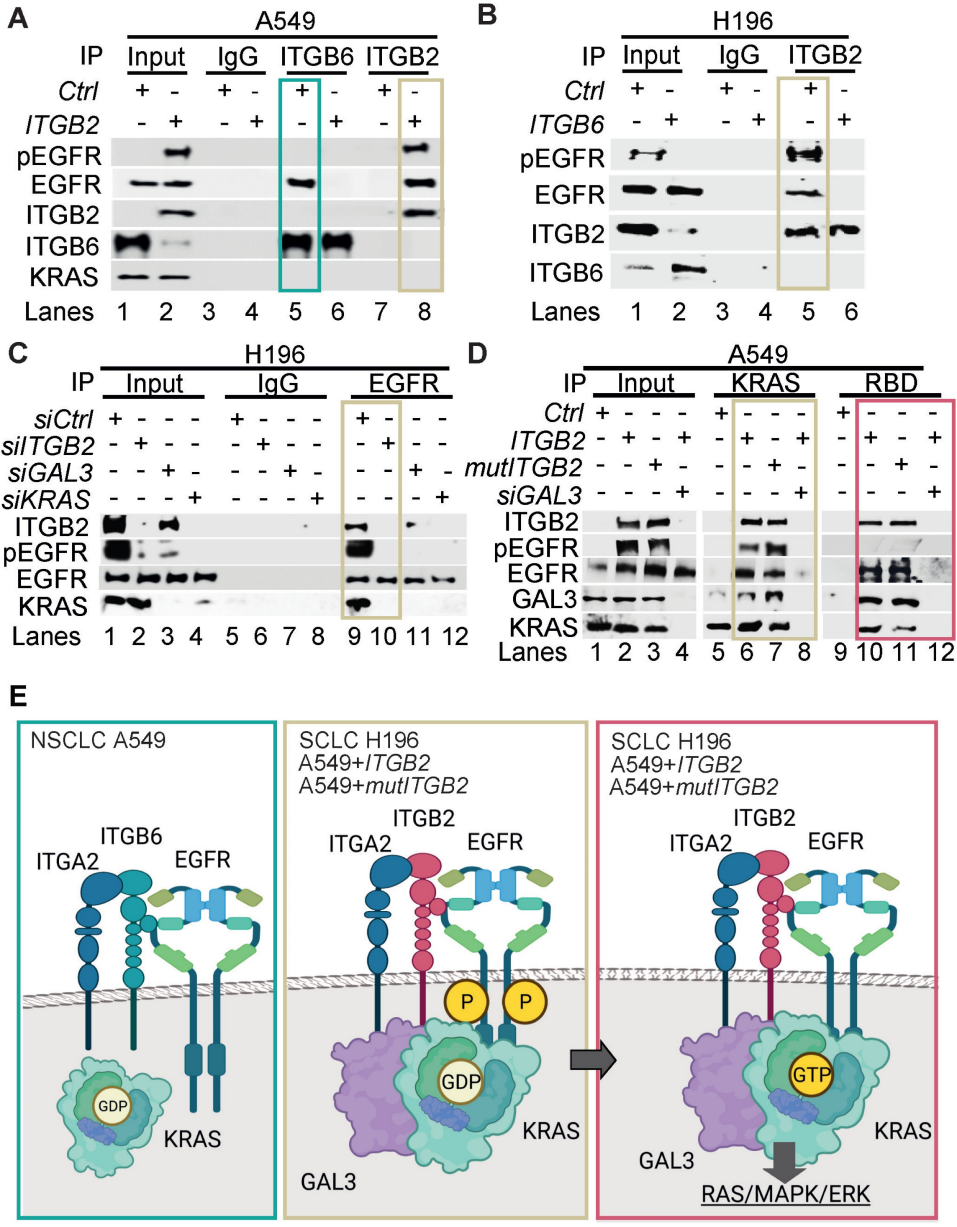
** Different multimeric protein complexes sequentially occur during non-canonical ITGB2-mediated activation of KRAS/MAPK/ERK signaling in SCLC.** (**A**) Total protein extracts of A549 cells transfected with empty vector (*Ctrl*) or *ITGB2* were immunoprecipitated (IP) using either immunoglobulin G (IgG, as control) or ITGB6 and ITGB2-specific antibodies. Co-IP proteins were analyzed by WB using the indicated antibodies. Input, 5% of material used for the IP. Squares indicate conditions in which endogenous ITGB6 interacts with inactive EGFR (green) and overexpressed ITGB2 interacts with active pEGFR (gold). (**B**) Total protein extracts of NCI-H196 cells transfected with empty vector (*Ctrl*) or *ITGB6* were immunoprecipitated (IP) using either immunoglobulin G (IgG, as control) or ITGB2-specific antibodies. Co-IP proteins were analyzed by WB using the indicated antibodies. Input, 5% of material used for the IP. Gold square indicates conditions in which endogenous ITGB2 interacts with endogenous, active pEGFR. (**C**) Protein extracts of NCI-H196 cells transfected with small interfering RNA specific for *ITGB2* (*siITGB2*), *GAL3* (*siGAL3*) or *KRAS* (*siKRAS*) were immunoprecipitated (IP) using either immunoglobulin G (IgG, as control) or EGFR-specific antibodies. Co-IP proteins were analyzed by WB using the indicated antibodies. In, input, 5% of material used for the IP. Gold square indicates conditions showing the ITGB2-pEGFR-interaction is specific and GAL3- and KRAS-dependent. (**D**) RAS activation assay. Protein extracts of A549 cells transfected with *ITGB2* or ligand-binding-deficient D134A ITGB2 mutant (*mutITGB2*) and *siGAL3* were immunoprecipitated (IP) using KRAS-specific antibody (KRAS) or RAF-RBD (RBD, active KRAS) coated beads. Co-IP proteins were analyzed by WB using the indicated antibodies. In, input, 5% of material used for the IP. Gold square highlights conditions in which KRAS interacts with ITGB2, mutITGB2, GAL3, EGFR and pEGFR in GAL3-dependent manner. Magenta square highlights conditions in which active, GTP-bound KRAS interacts with ITGB2, mutITGB2, GAL3 and EGFR, but not with pEGFR. (**E**) Model. Left, endogenous ITGB6 interacts with EGFR in the NSCLC cell line A549. Middle, endogenous ITGB2 interacts with endogenous pEGFR in the SCLC cell line NCI-H196, or in A549 cells after *ITGB2* or *mutITGB2* transfection. Further, results from RAS activation assays indicate the formation of a multimeric protein complex in two different forms, one form containing ITGB2, pEGFR, GAL3 and inactive, GDP-bound KRAS (middle) and the other form containing ITGB2, EGFR, GAL3 and active, GTP-bound KRAS (right), both forms occurring in sequential order during non-canonical ITGB2-mediated activation of KRAS/MAPK/ERK signaling in SCLC. See also Source Data S2.

To gain further insight into the mechanism of ITGB2-induced activation of EGFR, we performed Co-IP using ITGB6- or ITGB2-specific antibodies in protein extracts from A549 cells transiently transfected with *Ctrl* (empty vector), *ITGB6* or *ITGB2* (Figure 6A). We found that endogenous ITGB6 interacts with non-phosphorylated EGFR and *ITGB2* overexpression abolished this interaction. Further, *ITGB2* transfection induced pEGFR and reduced ITGB6 levels, confirming our previous results (Figure 1F, 2A and S5B-C). Moreover, over-expressed ITGB2 co-precipitated pEGFR demonstrating the interaction of ITGB2 with the active form of this receptor. These results were complemented by Co-IP experiments using protein extracts from NCI-H196 cells transiently transfected with *Ctrl* or *ITGB6* (Figure 6B). We detected interaction of endogenous ITGB2 with pEGFR, confirming the results in A549 cells after overexpression of *ITGB2*. Further, *ITGB6* overexpression abolished the ITGB2-pEGFR-interaction. Our results demonstrate the interaction between endogenous ITGB6 and EGFR in the NSCLC cell line A549, whereas endogenous ITGB2 interacts with pEGFR in the SCLC cell line NCI-H196. Moreover, these interactions appear mutually exclusive, since overexpression of *ITGB2* or *ITGB6* abolished the interaction of the complementary integrin receptor beta subunit with EGFR. Remarkably, precipitation of endogenous EGFR in NCI-H196 cells confirmed the pEGFR-ITGB2 interaction (Figure 6C). Moreover, siRNA-mediated LOF of *ITGB2*, *GAL3* or *KRAS* abolished the pEGFR-ITGB2 interaction, thereby demonstrating the specificity of the pEGFR-ITGB2 interaction and the requirement of GAL3 and KRAS for this interaction. Further Co-IP experiments in A549 cells (Figure 6D) showed that endogenous KRAS interacted with overexpressed ITGB2, mutITGB2, endogenous EGFR, pEGFR and GAL3 in a GAL3-dependent manner (lanes 5 to 8). Interestingly, pull down of active, GTP-bound KRAS using RAF-RBD coated beads (lanes 9 to 12) co-precipitated overexpressed ITGB2, mutITGB2, GAL3 and endogenous EGFR, but not pEGFR, in a GAL3-dependent manner. Our results support that the KRAS fraction interacting with pEGFR is inactive (compare lanes 5 to 8 with 9 to 12). The model in Figure 6E summarizes our results. Endogenous ITGB6 interacts with EGFR in the NSCLC cell line A549 (Figure 6E, left). The ITGB6-EGFR-interaction is abolished upon *ITGB2* or *mutITGB2* transfection, suggesting a functional switching of a complex containing ITGB6-EGFR in NSCLC to a complex containing ITGB2-EGFR in SCLC. Supporting this line of ideas, over-expressed *ITGB2* or *mutITGB2* in A549 cells, or endogenous ITGB2 in the SCLC cell line NCI-H196 interact with endogenous pEGFR (Figure 6E, middle). However, our results indicate the formation of a multimeric protein complex in at least two different forms. One form contains ITGB2, pEGFR, GAL3 and inactive, GDP-bound KRAS (Figure 6E, middle). The other form contains ITGB2, EGFR, GAL3 and active, GTP-bound KRAS (Figure 6E, right). Considering the results presented in Figure 1, 2 and 5, we propose that these forms of the multimeric protein complex occur in sequential order during non-canonical ITGB2-mediated activation of KRAS/MAPK/ERK signaling in SCLC.

### Extracellular vesicles containing ITGB2 activate RAS/MAPK/ERK signaling and induce SCLC proteins

**Figure 7:**
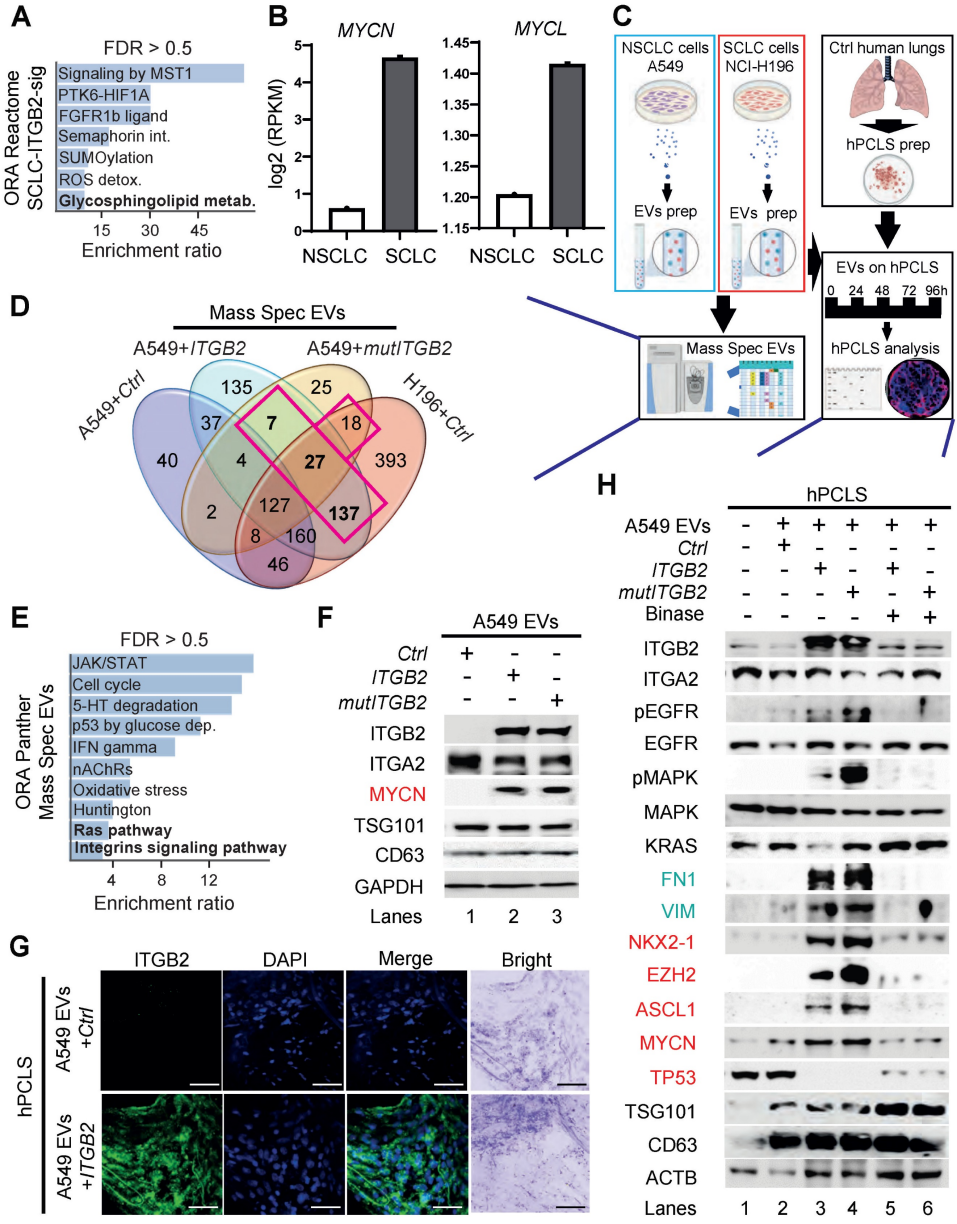
** Extracellular vesicles containing ITGB2 activate RAS/MAPK/ERK signaling and induce SCLC proteins.** (**A**) Reactome-based enrichment analysis of significant pathways in the 93 IDs of the SCLC-ITGB2 gene expression signature (SCLC-ITGB2-sig) from Figure 3E using Webgestalt bioinformatics tool and plotted by highest enrichment ratio. Int., interactions; metab., metabolism. (**B**) RNA-seq-based expression analysis of indicated transcripts in non-small cell lung cancer (NSCLC; *n* = 33) and small cell lung cancer (SCLC; *n* = 17) cell lines. Values were normalized to *GAPDH* and represented as log2 of reads per kilobase per million (RPKM). Bar plots show data as means; error bars, SD. (**C**) Scheme of experiments with EVs isolated from the cell culture medium of NSCLC and SCLC cell lines. Characterization of the protein cargo of the isolated EVs by high-resolution mass spectrometry (HRMS) analysis. Characterization of human precision-cut lung slices (hPCLS) that were treated with isolated EVs. (**D**) Venn diagram comparing proteins that were detected by HRMS in EVs from control transfected A549 cells, NCI-H196 cells, as well as from A549 cells transfected either with *ITGB2* or *mutITGB2* highlights a group of 189 proteins that are common for the last 3 conditions. See also Table S5. (**E**) Panther-based enrichment analysis of significant pathways in the 189 proteins highlighted in D, using Webgestalt bioinformatics tool and plotted by highest significance enrichment ratio. (**F**) Total protein extracts of EVs from A549 cells transfected with *ITGB2* or *mutITGB2* were analyzed by WB using the indicated antibodies. (**G**) Confocal microscopy after immunostaining with specific antibodies against ITGB2 in hPCLS incubated with EVs from A549 cells previously transfected with *Ctrl* or *ITGB2*. DAPI, nucleus. Scale bars, 500 μm. (**H**) Total protein extracts of hPCLS incubated with EVs from A549 cells previously transfected with *ITGB2* or *mutITGB2* alone or in combination with binase were analyzed by WB using the indicated antibodies. Products of downstream gene targets of EGF signaling (green) and SCLC proteins (red) are highlighted. See also Figure S8, S9 and Data Source S2.

ORA of the SCLC-ITGB2-sig based on the Reactome database revealed significant enrichment of genes related to glycosphingolipid metabolism (Figure 7A). In addition, we found elevated expression of the proto-oncogenes *MYCN* and *MYCL* in SCLC cell lines (Figure 7B). Furthermore, GO enrichment analysis based on Biological Processes of transcripts with high levels in SCLC cell lines revealed significant enrichment of genes related to the GO term “Vesicle transport” (Figure S8A-B). Since all these events are related to the secretion of EVs 48, we isolated EVs from the cell culture medium, in which A549, NCI-H82 and NCI-H196 cells were grown, and characterized them by various downstream analyses (Figure 7C and S8C). EVs produced by these three cell lines showed similar size with a radius of approximately 45 nm (Figure S8C). Further, high-resolution mass spectrometry analysis of the protein cargo of the isolated EVs revealed 189 proteins that were common in EVs from NCI-H196 cells transfected with control plasmid (*Ctrl*) and from A549 cells transfected either with *ITGB2* or *mutITGB2* (Figure 7D; Table S5 and Figure S8D). Panther-based ORA of these common 189 proteins (Figure 7E) showed significant enrichment of proteins involved in “Ras pathway” and “Integrins signaling pathway”, correlating with our previous results (Figure 1-6). In addition, ORA using the KEGG database for pathways enriched in the common 189 proteins (Figure S8E) revealed significant enrichment of the oxidative phosphorylation pathway correlating with a previously reported metabolic switch in cancer associated fibroblasts mediated by ITGB2 49. Further, WB of the protein cargo of EVs produced by A549 cells that were transiently transfected with *Ctrl*, *ITGB2* or *mutITGB2* (Figure 7F) showed similar levels of ITGA2, as well as of the EVs markers TSG101 (tumor susceptibility gene 101 protein) and the CD63 antigen 50, 51, whereas ITGB2 and MYCN were specifically detected after *ITGB2* or *mutITGB2* transfection, confirming the mass spectrometry results (Table S5) and suggesting that ITGA2-ITGB2 heterodimers and MYCN are transferred from the EVs producing cells to hPCLS. Interestingly, the mass spectrometry results showed not significant differences in the levels of cell adhesion proteins in the EVs produced by A549 and NCI-H196 cells transfected as described above (Figure S8F).

In addition, confocal microscopy in sections of hPCLS that were incubated with EVs produced by A549 cells revealed ITGB2 staining when the A549 cells were transfected with *ITGB2* prior EVs isolation (Figure 7G). These results together support that EVs can influence the gene expression signature of the treated hPCLS and argue against an increased uptake of EVs by hPCLS under the conditions analyzed here. This interpretation was confirmed by WB of protein extracts from hPCLS incubated with A549 EVs (Figure 7H). Protein extracts of hPCLS showed increased ITGB2 levels when they were incubated with EVs produced by A549 cells that were transfected with *ITGB2* or *mutITGB2*. Interestingly, EVs produced by A549 cells transfected with *ITGB2* or *mutITGB2* induced phosphorylation-dependent activation of EGFR and MAPK, as well as increased levels of the downstream targets of EGFR signaling VIM and FN1, demonstrating activation of the RAS/MAPK/ERK signaling pathway in the treated hPCLS. Moreover, EVs isolated from *ITGB2*- or *mutITGB2*-transfected A549 cells induced NKX2-1, EZH2, ASH-1 and MYCN in treated hPCLS, whereas TP53 levels were reduced, thereby mimicking gene expression patterns that are characteristic of SCLC 52, 53. As we have previously shown that the ribonuclease (RNase) binase inhibits oncogenic KRAS 47, we included binase treatment in our experimental setting. Remarkably, binase treatment counteracted the effects caused by EVs isolated from *ITGB2*- or *mutITGB2*-transfected A549 cells, thereby demonstrating the causal involvement of KRAS activation.

Since EVs produced by *ITGB2*- or *mutITGB2*-transfected A549 cells contained the transcription factor MYCN (Figure 7F and Table S5) and EVs seem to influence gene expression of hPCLS (Figure 7G-H), we tested whether the *ITGB2* gene is a direct target of MYCN. Published data of next generation sequencing (NGS) after chromatin immunoprecipitation (ChIP-seq) using MYC-specific antibodies 54 and assay for transposase-accessible chromatin with sequencing (ATAC-Seq) 55 revealed enrichment of MYC at the *ITGB2* promoter in an area of the genome with accessible chromatin and with predicted binding elements for MYC (Figure S9A). Since MYC proteins dimerize with MAX to regulate transcription of a broad number of genes 56, 57, we also retrieved published CUT&RUN sequencing data, in which MAX-specific antibodies and controlled cleavage by micrococcal nuclease were combined 58 and found also MAX enrichment in the same area of the at the *ITGB2* promoter as MYC (Figure S9A). These results suggest that the *ITGB2* gene is directly regulated by MYC-MAX heterodimers. Supporting this interpretation, lung tissue from SCLC patients in C2 showed significantly higher *MYC* expression (median = 1.82; IQR = 1.10; *n* = 6) than the lung tissue from SCLC patients in C1 (median = 0.78; IQR = 0.84; *n* = 6, *P* = 0.006; Figure S9B left; Source Data S1). Similar results were observed for the expression of *MAX* and *VIM* (Figure S9B middle and right; Source Data S1). Moreover, qRT-PCR-based expression analysis using total RNA extracted from A549 cells (Figure S9C; Source Data S1) showed significantly increased *ITGB2* levels after treatment with EVs isolated from NCI-H196 cells (mean = 1.38; SD = 0.09; *n* = 3) as compared to non-treated cells (mean = 0.75, SD = 0.25; *n* = 3, *P* = 0.01). We also observed increased expression of the of the downstream targets of EGFR signaling *VIM*, *HIF1A* and *FN1* in A549 cells treated with EVs isolated from NCI-H196 cells as compared to non-treated cells (Figure S9C; Source Data S1). These results confirmed that EVs from NCI-H196 cells activated the RAS/MAPK/ERK signaling pathway and influenced gene expression in the treated A549 cells.

### ITGB2 loss-of-function and binase inhibit SCLC-associated proteins

**Figure 8:**
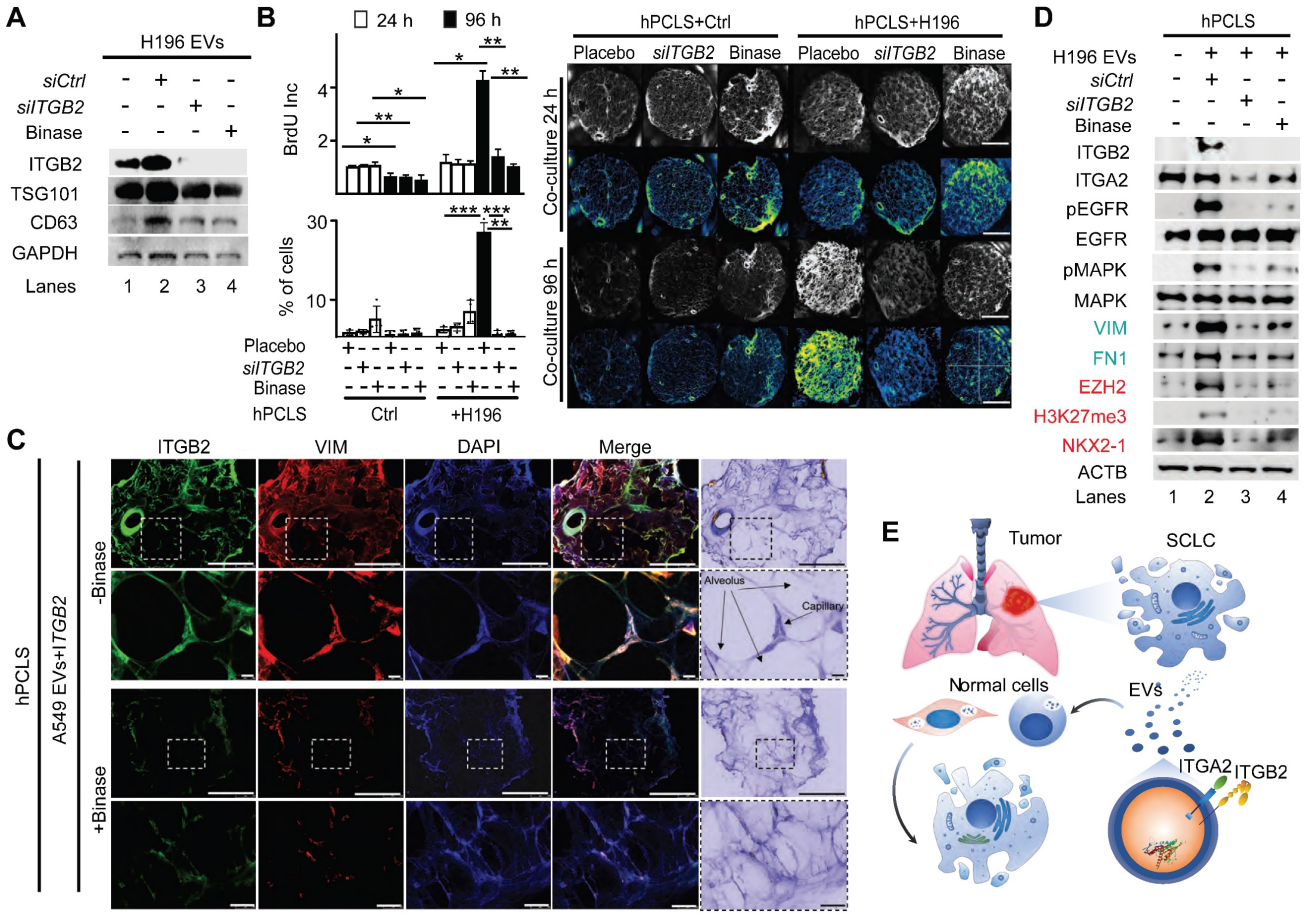
** ITGB2 loss-of-function and binase inhibit SCLC.** (**A**) Total protein extracts of EVs from NCI-H196 cells transfected with control or *ITGB2*-specific small interfering RNA (*siCtrl* or *siITGB2*), or treated with binase, were analyzed by WB using the indicated antibodies. (**B**) Left, cell proliferation of hPCLS co-cultured without or with NCI-H196 cells previously transfected with *siITGB2* or treated with binase was measured by the colorimetric method using BrdU incorporation (top) cell number quantification (bottom). Data are shown as means ± SD (*n* = 3 independent experiments); asterisks, *P*-values after two tailed t-test, *** *P* ˂ 0.001; ** *P* ˂ 0.01; * *P* ˂ 0.05. Right, representative live microscopy images to the bar plots. Quadrants used for quantification are indicated. Scale bars, 500 μm. (**C**) Confocal microscopy after immunostaining with specific antibodies against ITGB2 and VIM in hPCLS incubated with EVs from A549 cells previously transfected with *ITGB2* and non-treated or treated with binase. DAPI, nucleus. Scale bars, 500 μm. Squares are respectively shown below at higher magnification. (**D**) Total protein extracts of hPCLS incubated with EVs from NCI-H196 cells previously transfected with *siCtrl* or *siITGB2* alone or in combination with binase were analyzed by WB using the indicated antibodies. Products of downstream gene targets of EGF signaling (green) and SCLC proteins (red) are highlighted. (**E**) Model. In SCLC, high ITGB2 induces a KRAS-driven secretory phenotype of ITGB2/ITGA2 loaded EVs, which total protein cargo induces a SCLC-like phenotypic transformation in normal cells. See also Figure S10, S11 and Source Data S1 and S2.

Since EVs produced by *ITGB2*- or *mutITGB2*-transfected A549 cells were able to induce RAS/MAPK/ERK signaling and SLCL markers in hPCLS (Figure 7H), we decided to investigate the EVs produced by the SCLC cell line NCI-H196 either non-transfected or transfected with *siCtrl* or *siITGB2* or treated with binase (Figure 8). WB of the protein cargo of EVs produced by non-transfected or *siCtrl*-transfected NCI-H196 cells showed similar levels of ITGB2, MYCN, TSG101 and CD63 (Figure 8A), whereas *siITGB2* transfection or binase treatment of the NCI-H196 cells prior EVs isolation reduced the ITGB2 and MYCN levels in the isolated EVs. Further, we analyzed the effects caused on hPCLS by EVs produced by NCI-H196 cells under the four conditions specified above (Figure 8B-D). EVs produced by *siCtrl*-transfected NCI-H196 cells increased cell proliferation and cell number of hPCLS after 96h treatment (Figure 8B).

We also detected ITGB2 and VIM by confocal microscopy in sections of hPCLS that were incubated with EVs produced by A549 cells transfected wit *ITGB2* (Figure 8C), supporting that such EVs induced in the treated hPCLS gene expression patterns that are similar to SCLC. These results were complemented by WB of protein extracts from hPCLS incubated with NCI-H196 EVs (Figure 8D). EVs produced by NCI-H196 cells induced in hPCLS ITGB2, phosphorylation-dependent activation of EGFR and MAPK, as well as increased levels of the downstream targets of EGFR signaling VIM and FN1, demonstrating activation of the RAS/MAPK/ERK signaling pathway in the treated hPCLS. Moreover, EVs produced by NCI-H196 cells induced EZH2, H3K27me3 (Histone 3 tri-methylated at lysine 27) and NKX2-1 in treated hPCLS, thereby mimicking gene expression patterns that are characteristic of SCLC 52, 53. Remarkably, the effects induced by NCI-H196 EVs on hPCLS were counteracted by *siITGB2* transfection or binase treatment of NCI-H196 cells prior EVs isolation (Figure 8B-D), suggesting both, ITGB2-LOF as well as binase treatment, for the development of therapies against SCLC. Supporting this interpretation, Table S6 summarizes pre-clinical and clinical studies, in which binase and other RNases have been tested in the context of different cancer types.

We decided to determine the effects of binase treatment and ITGB2-LOF on cancer hallmarks in SCLC cells (Figure S10-S11). Binase treatment of NCI-H82 and NCI-H196 cells reduced the levels of KRAS, pEGFR and VIM (Figure S10A), as well as reduced cell viability and colony formation (Figure S10B-C). On the other hand, *ITGB2* knockout using CRISPR-Cas9 gene-editing technology 59 in NCI-H196 cells was lethal with all five guide RNAs used, supporting the requirement of the *ITGB2* gene for this SCLC cell line. Further, we targeted the *ITGB2* gene using CRISPR-Cas9 technology in A549 cells and in the human hepatocellular carcinoma cells HepG2, a liver cell line exhibiting epithelial-like morphology, being the liver an organ that arises from the embryonic endoderm as the lung. CRISPR-Cas9-mediated *ITGB2* knockout in A549 and HepG2 cells (Figure S10D) reduced colony formation and cell viability in both cell lines as compared to the control cells (Figure S10E-G). A literature search summarized in Table S7 shows that resistance to EGFR-Tyrosine kinase-inhibitors (EGFR-TKI) in SCLC occurs without any of the known somatic mutations in EGFR responsible for acquired EGFR-TKI resistance observed in NSCLC. On the other hand, our results showed that ITGB2 is sufficient and required for EGFR activation in SCLC (Figures 2, 5, 6, 7 and 8), suggesting that targeting *ITGB2* may sensitize SCLC cells to EGFR-TKI treatment. To test this hypothesis, we analyzed by WB protein extracts from both SCLC cell lines, NCI-H82 and NCI-H196, that were treated with the EGFR-TKI Erlotinib 60 after *siCtrl* or *siITGB2* transfection (Figure S11A). Erlotinib treatment after *siCtrl* did not reduce the levels of the EGF signaling marker VIM in either SCLC cell line, showing the resistance of these cells to the EGFR-TKI. Interestingly, in NCI-H196 cells *siITGB2* was sufficient to reduce VIM levels, which were not further reduced after Erlotinib treatment. However, in NCI-H82 cells *siITGB2* alone was not sufficient to reduce VIM levels, but combining *siITGB2* and Erlotinib did. Thus, we decided to determine the effect of *siITGB2* on cell proliferation, one of the cancer hallmarks, by BrdU (5-Bromo-2´-Deoxyuridine) incorporation assay (Figure S11B). Erlotinib treatment did not affect cell proliferation in either SCLC cell line. However, Erlotinib in combination with *siITGB2* reduced cell proliferation. Our results indicate that *ITGB2*-LOF may be beneficial against SCLC, since it sensitizes SCLC cells to EGFR-TKI treatment.

## Discussion

In the present study, we showed that ITGB6 interacts with the inactive EGFR in NSCLC, whereas in SCLC, ITGB2 reduces the levels of ITGB6, and interacts with and activates EGFR. In addition, we demonstrated that the EVs produced by a SCLC cell line induced in hPCLS both, non-canonical ITGB2-mediated activation of KRAS/MAPK/ERK signaling and SCLC proteins, supporting the hypothesis that the cargo of EVs may influence the gene expression signature of hPCLS (Figure 8D). Following this line of ideas, siRNA-mediated *ITGB2*-LOF or binase treatment of the SCLC cell line led to the production of EVs that lost the capacity to induce KRAS/MAPK/ERK signaling and SCLC proteins in hPCLS. On the one hand, our results provide a model (Figure 8E) to study the effects of EVs on specific tissues by targeted cargo modification through manipulation of the gene expression in EV-producing cells. On the other hand, they also provide a plausible explanation for the resistance of SCLC to tyrosine EGFR-TKIs (including Erlotinib, Gefitinib and Afatinib, Table S7), and suggest the development of novel therapeutic strategies for SCLC combining EGFR-TKIs and ITGB2-LOF. Our results showing that ITGB2-LOF sensitized SCLC cell lines to Erlotinib (Figure S11A-S11B) support this line of ideas. Alternatively, treating the lung of SCLC patients with EVs produced by NSCLC cell lines to achieve lung tissue sensitivity to EGFR-TKIs may be one treatment strategy worthy of exploration. Furthermore, determining high levels of ITGB2 in cases of NSCLC showing enhanced EGFR signaling in the absence of somatic mutations may validate ITGB2 as a promising therapeutic target in NSCLC. This reasoning is supported by the current use of EGFR-TKIs in lung adenocarcinoma patients with EGFR mutations 61, 62. The majority of these hyperactive EGFR mutants harbor either a point mutation, in which leucine 858 is substituted by arginine (L858R), or a deletion involving 5 codons coding for the amino acids at the positions 746-750 (ΔEx19) 63. However, a secondary point mutation in the EGFR kinase domain that substitutes threonine 790 by methionine (T790M) produces drug-resistant EGFR variants, which are present in 50% of patients that developed resistance to EGFR-TKIs treatment 64. Surprisingly, studies of biopsies have shown that 5-15% NSCLC patients undergo histological transformation to SCLC upon acquisition of therapy resistance 65. It will be extremely interesting to determine whether this histological transformation from NSCLC to SCLC upon acquired EGFR-inhibitor resistance is the result of a functional switch from ITGA2-ITGB6 to ITGA2-ITGB2 during EGFR complex formation.

Various aspects of the signaling model proposed here may be applicable in a broader context. Specific integrin receptor subunits have been identified as biological markers and potential therapy targets to tumor progression and metastasis in a wide range of cancers including glioblastoma, pancreatic carcinomas, breast cancer, and leukemia 33, 66. In addition, recent reports demonstrated functional correlation between the switch of specific integrin subunits and an aggressive phenotype of cancer cells. For instance, ITGA2-ITGB1 promotes chemotherapy resistance of T-cell acute lymphoblastic leukemia 67. Further, ITGB1 has been reported to trigger EGFR ligand-independent proliferation signaling in pancreatic ductal adenocarcinoma, bypassing the EGFR-blocking effect of the anti-EGFR monoclonal antibody Cetuximab 68. These reports suggest that targeting specific integrin subunits may be beneficial against a wider spectrum of cancer types. Supporting this hypothesis, depletion of ITGB3, ITGB4 and ITGB5 reduced angiogenesis and tumor growth in breast cancer 31.

Another interesting aspect of our model is the functional competition between ITGA2-ITGB2 in SCLC and ITGA2-ITGB6 in NSCLC. Similar competitions have been observed between ITGAM-ITGB2 and ITGA5-ITGB1 or ITGAv-ITGB3 and ITGA5-ITGB1 heterodimers regulating migration or trafficking of leukocytes 69, 70. Recently, a mutual competition between ITGA5-ITGB1 blocking EGF signaling and ITGA6-ITGB4 enhancing EGF signaling has been reported 71, highlighting the specific interaction between integrin subunits mediating different functions in EGF signaling. Interestingly, EGFR interacts in the cell membrane with glycosylated regulatory partners including proteoglycans like syndecans 72, 73. Remarkably, *N*-glycosylation of specific domains in the ITGA5 subunit appears critical to different processes mediating its biological function, such as ITGA5-ITGB1 heterodimer formation, its expression on the cell surface, ligand binding, EGFR-ITGA5-ITGB1 complex formation and its inhibitory effect on EGFR 71, 74, 75. Similar to ITGA5, it has been also reported that *N*-glycosylation of ITGB4 is essential for EGFR-ITGB4-ITGA6 complex formation on the cell surface 76. Future work will determine whether a similar mechanism of *N*-glycosylation participates in switching the EGFR complex formation from ITGA2-ITGB6 to ITGA2-ITGB2 in the course of the histological transformation from NSCLC to SCLC observed upon acquired EGFR-TKIs resistance 65.

We uncovered a mechanism of non-canonical ITGB2-mediated EGFR activation that explains EGFR-TKIs resistance in SCLC cases lacking EGFR mutations. Our results were obtained implementing *in vitro* and *ex vivo* experimental systems including human cells and tissue, and they may be confirmed and further investigated using *in vivo* animal models in future projects. Nevertheless, our results not only support the use of ITGB2 and the newly identified SCLC-ITGB2-sig as diagnostic markers for SCLC, but also as targets to develop therapeutic strategies against this extremely aggressive type of LC.

## Methods

### Study population

The study was performed according to the principles set out in the WMA Declaration of Helsinki and the protocols approved by the institutional review board and ethical committee of Regional Hospital of High Specialties of Oaxaca (HRAEO), which belongs to the Ministry of Health in Mexico (HRAEO-CIC-CEI 006/13), the Medicine Faculty of the Justus Liebig University in Giessen, Germany (Ethical Votum 68/13) and the Hannover Medical School (no. 2701-2015). In this line, all patient and control materials were obtained through the HRAEO in Mexico, the Institute for Pathology of Justus Liebig University in Giessen and the Biobank from the Institute for Pathology of the Hannover Medical School as part of the BREATH Research Network. We used anonymized patient material.

Formalin-fixed paraffin-embedded (FFPE) human lung tissue samples of either diagnostic transbronchial or bronchial biopsies or oncologic resections were retrospectively collected. All cases were reviewed and staged by an expert panel of pulmonologist and oncologist. FFPE tissue samples of LC patients comprised approximately 80% tumor cells. The control population for the analysis included lung tissue that was taken from macroscopically healthy adjacent regions of the lung of LC patients. Corresponding clinical data for matched patients with LUAD (*n* = 11) were obtained from The Genome Cancer Atlas (TCGA, tcga‑data.nci.nih.gov/doc/publications/tcga/). Data are publicly available and open‑access. Clinical characteristics of LUAD patients are presented in Table S3.

### Cell culture, transfection, treatment and siRNA-mediated knockdown

Mouse lung epithelial cells MLE-12 (ATCC CRL-2110) were cultured in complete DMEM/F12 (5% FCS, 1% Penn-strep) at 37 ^°^C in 5% CO_2_. Human SCLC cells NCI-H82 (ATCC HTB-175) and NCI-H196 (ATCC CRL-5823), and NSCLC cells A549 (ATCC CCL-185) were cultured in complete RPMI (10% FCS, 1% Penn-strep) at 37 °C in 5% CO_2_. During subculturing, cells were 1x PBS washed, trypsinized with 0.25% (w/v) Trypsin and subcultivated at the ratio of 1:5 to 1:10. The cell lines used in this paper were mycoplasma free. They were regularly tested for mycoplasma contamination. In addition, they are not listed in the database of commonly misidentified cell lines maintained by ICLAC. Cells were transfected with plasmid DNA or siRNA using Lipofectamine 2000 (Invitrogen) following the manufacturer's instructions, and harvested 48 h later for further analysis. *ITGA2-HIS* (Addgene, #51910), *ITGB2-YFP* (Addgene, #8638) and *ITGB6-GFP* (Addgene, #13293) mammalian expression constructs were used for respective gene overexpression in cell lines. *siITGB2* (EHU133911) was purchased from Sigma. Empty vectors and *siCtrl* were used as a negative control.

### Bacterial culture and cloning

For cloning experiments, chemically competent *E. coli* TOP10 (ThermoFisher Scientific) were used for plasmid transformation. TOP10 strains were grown in Luria broth (LB) at 37 °C with shaking at 180 rpm for 16 h or on LB agar at 37 °C overnight.

### RNA isolation, reverse transcription, quantitative PCR and TaqMan assay

Expression analysis by qRT-PCR were performed as previously described 77. Briefly, total RNA from cell lines was isolated using the RNeasy Mini kit (Qiagen) and quantified using a Nanodrop Spectrophotometer (ThermoFisher Scientific, Germany). Human lung tissue samples were obtained as FFPE tissues, and eight sections of 10 μm thickness were used for total RNA isolation using the RecoverAll Total Nucleic Acid Isolation Kit for FFPE (Ambion). Clinical characteristics of SCLC patients are presented in Table S2. Synthesis of cDNA was performed using 0.2-1 µg total RNA and the High-Capacity cDNA Reverse Transcription kit (Applied Biosystems). Quantitative real-time PCR reactions were performed using SYBR^®^ Green on the Step One plus Real-time PCR system (Applied Biosystems). Housekeeping genes *HPRT* and *GAPDH* were used to normalize gene expression. Primer pairs used for gene expression analysis are described in Table 1.

### Immunofluorescence and confocal microscopy

Immunostaining was performed as previously described 78. Briefly, cells were grown on coverslips, fixed with 4% PFA for 10min at RT and permeabilized with 0.4% Triton-X100/1xPBS for 10 min at RT. For non-adherent cells, slides were previously coated with poly-L-lysine. During immunostaining procedure, all incubations and washes were performed with histobuffer containing 3% bovine serum albumin and 0.2% Triton X-100 in 1xPBS, pH 7.4. Non-specific binding was blocked by incubating with donkey serum and histobuffer (1:1 (v/v) ratio) for 1 h. Cells were then incubated with primary and then secondary antibodies for 1 h followed by nuclear staining. Immunostaining of cells were examined with a confocal microscope (Zeiss). Antibodies used were specific anti-ITGB2 (R&D Systems), anti-ITGB6 (R&D Systems), anti-GFP (Santa Cruz), anti-pEGFR (Cell Signaling, Antibody #2237, for phospho Tyr1045), and anti-EGFR (Cell Signaling, Antibody #2232), were used. Alexa 488, Alexa 555 or Alexa 594 tagged secondary antibodies (Invitrogen, Germany, dilution 1:2000) were used. DAPI (Sigma, Germany) used as nuclear dye.

Paraffin-embedded lung tissue sections (3-μm thick) were deparaffinized in xylene and rehydrated in a graded ethanol series to PBS (pH 7.2). Antigen retrieval was performed by pressure cooking in citrate buffer (pH 6.0) for 15 min. Double immunofluorescence staining was performed with primary antibodies anti-ITGB2 (R&D Systems), anti-ITGB6 (R&D Systems), anti-GFP (Santa Cruz), anti-VIM (Cell Signaling) and anti-ASH1 (Chemicon) were used. After overnight incubation with specific primary antibodies, slides were washed and incubated with the respective secondary antibodies, Alexa 488-, Alexa 555- and Alexa 594-conjugated goat anti-rabbit IgG (dilution 1:1000, Molecular Probes, Eugene, OR) for 1 h. All sections were counterstained with nuclear DAPI (1:1000) and mounted with fluorescent mounting medium (Dako).

### Co-immunoprecipitation (Co-IP) and Western blot

Total protein extracts from different cell lines were prepared in 1 mL ice cold RIPA buffer [(50 mM (pH7.5) Tris-HCl, 150 mM NaCl, 1% Triton X-100 (Sigma), 0.5% Na-deoxycholate (Sigma), 0.1% SDS, 0.2 M imidazole (Sigma), 10 mM NaF (Sigma), 2 mM Na3VO4 (Sigma), 1 mM phenylmethylsulfonyl fluoride (PMSF) and protease inhibitor cocktail (Calbiochem)]. Detergent-insoluble material was precipitated by centrifugation at 14,000 rpm for 30 min at 4 °C. The supernatant was transferred to a fresh tube and stored at -20 °C. Protein concentration was estimated using Bradford assay, using serum albumin as standard. 5 μL of serial dilutions of standard protein and samples were mixed with 250 μL of Bradford reagent (500-0205, BIO-RAD Quick Start™). Samples were incubated 10 min at room temperature and measured at OD595 using an ELISA plate reader (TECAN Infinite M200 Pro). Co-IP was performed as described 79 with minor adaptations. For immunoprecipitation of membrane proteins, a total of 5 x 10^7^ cells were collected and washed three times in cold PBS, spun down at 270g for 10 min at 4 °C. Protein extracts were obtained as described above using cell immunoprecipitation (IP) buffer [(50 mM, pH 7.5) Tris-HCl, 1 mM MgCl2, 1 mM CaCl2, 150 mM NaCl, 1% NP40, 1 mM phenylmethylsulfonyl fluoride (PMSF) and protease inhibitor (Calbiochem)] Protein concentrations were determined as described above. Precleared protein lysates (500 μg per immunoprecipitation) were resuspended in 500 μL IP buffer and incubated with the appropriate antibodies on ice for 2 h and then 30 μL protein-G-sepharose beads (GE Healthcare; equilibrated once in 10 mL water and three times in washing buffer) were added and incubated overnight at 4 °C. Beads were collected and washed 5 times with 500 μL ice-cold washing buffer. 30 μL 2x SDS sample loading buffer was added to beads, boiled at 95 °C for 5 min, spun down and loaded on SDS-PAGE for western blot analysis. Western blotting was performed using standard methods and antibodies specific for 6x-HIS-Tag (Abcam), GFP (Santa Cruz), ITGB2 (R&D Systems), ITGB6 (R&D Systems), ITGA2 (R&D Systems), pMAPK (Cell Signaling), MAPK (Cell Signaling), pEGFR (Cell Signaling), EGFR (Cell Signaling), GAL3 (Santa Cruz), KRAS (Santa Cruz), pRAF1 (Cell signaling), RAF1 (Cell signaling), pERK (Cell signaling), ERK (Cell signaling), MYCN (Santa Cruz), TSG101 (Santa Cruz), CD63 (Santa Cruz), FN1 (Millipore), ACTA2 (Sigma), VIM (Cell Signaling), NKX2-1 (Santa Cruz), EZH2 (Abcam), H3K27me3 (Millipore), ASH-1 (Chemicon), TP53 (Cell signaling) and GAPDH (Sigma) were used. Immunoreactive proteins were visualized with the corresponding HRP-conjugated secondary antibodies (Santa Cruz) using the Super Signal West Femto detection solutions (ThermoFisher Scientific). Signals were detected and analyzed with Luminescent Image Analyzer (Las 4000, Fujifilm).

### KRAS activation assay

RAS Activation Assay Biochem Kit™ (BK008; Cytoskeleton, Inc) was used to assess KRAS activity following manufacturer's instructions. Briefly, A549, NCI-H196 and NCI-H82 cell protein lysates were produced in cell lysis buffer containing 50 mM Tris-HCl pH 7.5, 10 mM MgCl2, 500 mM NaCl, 2% Igepal, 0-5% BSA, 20 mM Imidazole, 20 mM NaF, 0.5 mM Na3VO4, 40 μg/mL PMSF and protease inhibitor) for 10 min at 37 °C on rotator with 200 rpm. RAF-RBD beads (100 μg) were added to the reactions and incubated at 4 °C on a rotator for 1 h. The beads were washed once with 500 μL wash buffer (25 mM Tris pH 7.5, 30 mM MgCl2, 40 mM NaCl, 20 mM Imidazole, 20 mM NaF, 0.5 mM Na3VO4, 40 μg/mL PMSF and protease inhibitor). Precipitated proteins were analyzed by Western Blotting as described above.

### Proliferation Assay

NCI-H196 cells were treated with Placebo, with binase or transfected with *siITGB2* and grown until 90-95% confluence in a 6-well plate. After 24 h or 96 h, cells were re-plated in 96-well plate in a density of 10^4^/well in a final volume of 100 μL and cultured in a humidified atmosphere at 37 °C. 10 μM BrdU (Cell proliferation, colorimetric kit, Sigma #11647229001) were added and the cells were further incubated for additional 6 h, fixed and washed. Subsequently the immunoassay was done by measuring the absorbance of the samples in an ELISA reader at 370 nm (reference wavelength: 492 nm).

### Generation of *ITGB2* knockout cell lines

Guide RNAs against human *ITGB2* were designed using CRISPOR tool 80, and cloned in BbsI (New England BioLabs, Cat# R3539S) digested pX459 V2 (Addgene Cat# 62988) vector. NCI-H196, A549 and HepG2 cells were transfected with control pX459 V2 vector and vector containing *ITGB2* guide RNAs. Cells were selected using 2.5 µg/mL puromycin for 48 h and ITGB2 protein expression level was checked by WB. Table 2 shows the sequences of the *ITGB2* guide RNAs used.

### Cell proliferation assay using CCK-8

1000 cells of each A549 and HepG2 were seeded in 96 well plate and incubated in 100 µL corresponding growth medium. At time points 24 h, 48 h, 72 h and 96 h, 10 µL CCK-8 reagent (Sigma-Aldrich, Cat#96992) was added and incubated for 2 h. Absorbance was measured at 450 nm using microplate reader.

### Colony Formation Assay

NCI-H196 cells transfected with *siCtrl* or *siITGB2* alone or in combination with binase treatment were plated in a 6-well culture plate at a density of 1000 or 5000 cells/well. The plate was swirled to ensure an even distribution of cells. The cells were grown in a 37 °C incubator with 5% CO2 for 3 to 5 d with media replacement every 2 d. At 10 d, the media was removed and cells were washed twice with PBS. The colonies were analyzed using ImageJ software (https://imagej.nih.gov/ij/). A549 (1500 cells/well) and HepG2 (1000 cells/well) cells were seeded in 6 well and incubated in corresponding growth medium for 1 week. Cells were washed with PBS, fixed using methanol and stained with 0.1% crystal violet solution in PBS. Number of colonies were counted using ImageJ.

### Protein-interaction prediction

Prediction of protein-protein interaction was observed using the STRING online database 81 (Retrieval of Interacting Genes-Proteins-http://string.embl.de/) with a cut-off criterion of a combined score 0.9 (highest confidence) and including a maximum of 50 interactors on the 1^st^ shell and 20 interactors on the 2^nd^ shell. Network nodes represent proteins, while edges are the protein‑protein associations. Small nodes represent protein of unknown 3D structure and large nodes proteins with some known or predicted 3D structures. Colored nodes represent query proteins and first shell of interactors and white nodes second shell of interactors. Interactions are depicted by color as follows: known interactions were obtained from curated databases (turquoise), or experimentally determined (purple); predicted interactions were defined by neighborhood (green), gene fusions (red) and gene co-occurrence (blue), textmining (light green), co-expression (black) and protein homology (violet). Top ITGA2 interactors were processed using the functional enrichment analyses Kyoto Encyclopedia of Genes and Genomes (KEGG). KEGG is the major public pathway‑associated database, which identifies significantly enriched metabolic pathways or signal transduction pathways in target genes compared with the whole genome background.

### RNA sequencing and data analysis

RNA sequencing generated for this paper was sequenced as previously described 78, 82. Briefly, total RNA from A549 (Ctrl, *ITGB2* or *mutITGB2*) and NCI-H196 cells was isolated using the Trizol method. RNA was treated with DNase (DNase-Free DNase Set, Qiagen) and repurified using the RNeasy micro plus Kit (Qiagen). Total RNA and library integrity were verified on LabChip Gx Touch 24 (Perkin Elmer). Sequencing was performed on the NextSeq500 instrument (Illumina) using v2 chemistry with 1 x 75 bp single end setup. Raw reads were visualized by FastQC to determine the quality of the sequencing. Trimming was performed using trimmomatic with the following parameters LEADING:3 TRAILING:3 SLIDINGWINDOW:4:15 HEADCROP:4, MINLEN:4. High quality reads were mapped using with STAR with reads corresponding to the transcript with default parameters. RNA-seq reads were mapped to human genome hg19. After mapping, Tag directories were obtained with MakeTagDirectory from HOMER (default setting). Samples were quantified by using analyzeRepeats.pl with the parameters (hg19 -count genes -rpkm; reads per kilobase per millions mapped). Gene expression was quantified in reads per kilo base million (RPKM). Expression values of zero were set to the overall minimum value and all data were log2 transformed. Genes expressed (log2 transformed expression > 0.2) were included in the analysis. The correlations of genes were measured using Pearson's correlation. Overlapping genes in the cell lines RNAseq dataset were processed using the functional enrichment analyses KEGG, Gene Ontology and Reactome.

### RNA sequencing meta-analysis

RNAseq data of human lung adenocarcinoma (LUAD) (n = 16) were downloaded from the TCGA data portal. The RNAseq data of SCLC and NSCLC cell lines was obtained from GEO under the accession number GSE30611 42. 34 out of 675 samples were selected as NSCLC and 17 as SCLC for analysis based on the following sample annotations: “Organism part” is lung and “Diseases” is lung carcinoma, lung adenocarcinoma, lung anaplastic carcinoma, non-small cell lung carcinoma, squamous cell lung carcinoma, large cell lung carcinoma, lung mucoepidermoid carcinoma, lung papillary adenocarcinoma, lung adenosquamous carcinoma, bronchioloalveolar adenocarcinoma, or squamous cell carcinoma. For details on the original processing of the data, refer to the original paper 37. The transcriptome profile in NCI-H82 and NCI-H196 was measured by the mean normalized expression of the genes in the A549 cell line.

For analysis of RNA-seq data retrieved from the European Genome Archive 83, RNAseq was performed on cDNA libraries prepared from PolyA+ RNA extracted from SCLC tissues and cells. A library with an insert size of 250 bp allowed to sequence 95 bp paired-end reads without overlap. Raw reads were visualized by FastQC to determine the quality of the sequencing. Trimming was performed using trimmomatic with the following parameters LEADING:3 TRAILING:3 SLIDINGWINDOW:4:15 HEADCROP:4, MINLEN:4. High quality reads were mapped using with STAR with reads corresponding to the transcript with default parameters. RNA-seq reads were mapped to human genome hg19. After mapping, Tag directories were obtained with MakeTagDirectory from HOMER (default setting). Samples were quantified by using analyzeRepeats.pl with the parameters (hg19 -count genes -rpkm; reads per kilobase per millions mapped). Gene expression was quantified in reads per kilo base million (RPKM). RPKM values were used to compare gene expression between clusters and calculate linear regression of expression.

### MYC-MAX binding site analysis

MYC ChIPseq bigwig files (GSM3073949) 54, MAX CUT&RUN bigwig files (GSM6222857) 58 and ATACseq bed files (GSM4729164) 55 were downloaded from the GEO repository. Integrative Genomics Viewer was used for data visualization 84 and ConTra v3 was used to calculate the predicted MYC-MAX binging sites 85.

### EVs purification, characterization and co-culture assays

For the collection of EVs, cells were cultured in media supplemented with 10% exosome-depleted FBS, in which EVs were depleted by overnight centrifugation at 100,000 g. Supernatants were then collected 72 h later for EV purification. Cell culture supernatants were centrifuged at 500 g for 5 min to pellet and discard cells, followed by 2,000 g for 30 min to remove cell debris and apoptotic bodies. A 1:1 volume of 2X PEG solution (16% w/v, polyethylene glycol, 1 M NaCl) was added. Samples were inverted to mix, then incubated overnight. Next day, medium/PEG mixture was centrifuged at 3,300 g for 1 h. Crude vesicle pellets were resuspended in 1 mL of exosome-depleted 1X PBS and re-pelleted by centrifugation at 100,000 g for 70 min at 4 °C (Beckman 45 Ti). Pellets at the bottom of the centrifugation tubes were resuspended in approximately 50 µL of 1X PBS. Differential Light Scattering (DLS) was used to validate the EVs size range. WB was performed as described above to characterize the protein cargo of EVs using specific antibodies to detect EVs constitutive markers. For co-culture assays, A549 cells cultured in exosome-free media were treated with the EVs isolated from NCI-H196 cells or NCI-H196 cells cultured in exosome-free media were treated with the EVs isolated from A549 cells using ExoQuick-TC Cat# EXOTC10A-1). Expression analysis by qRT-PCR was performed as previously described 77.

### Mass spectrometry: sample preparation, methods and data analysis

Extracellular vesicle samples were subjected to in gel digest as described 86. The resulting peptides were analyzed by liquid chromatography/tandem mass spectrometry (LC-MS2) utilizing in-house packed reverse phase column emitters (70 μm ID, 15 cm; ReproSil-Pur 120 C18-AQ, 1.9 μm, Dr. Maisch GmbH) and a buffer system comprising solvent A (5% acetonitrile, 0.1% formic acid) and solvent B (80% acetonitrile, 0.1% formic acid). The MaxQuant suite of algorithms (v.1.6.1.43) 87-89 was used for peptide/spectrum matching, protein group assembly as well as label free quantitation in the context of human Uniprot database (canonical and isoforms; downloaded on 2020/02/05; 210,349 entries). Relevant instrumentation parameters were extracted using MARMoSET 90 and are included in the supplementary material together with MaxQuant parametrization. Common proteins detected by LC-MS2 in the EVs were subjected to an estimated overall survival curve analysis for LUAD using KM plotter 45. KEGG pathway enrichment analysis was performed using the WebGestalt toolkit 91. Cell adhesion molecules (CAMs) retrieved from the KEGG database 92. The log-transformed label-free quantification (LFQ) intensities of the CAMs identified in the EVs were plotted.

### Experiments with human PCLS

PCLS were prepared from tumor free lung explants from patients who underwent lung resection for cancer at KRH Hospital Siloah-Oststadt-Heidehaus or the Hanover Medical School (both Hanover, Germany). Tissue was processed immediately within one day of resection as described before 93. Briefly, human lung lobes were cannulated with a flexible catheter and the selected lung segments were inflated with warm (37 °C) low melting agarose (1.5%) dissolved in Dulbecco's Modified Eagle's Medium Nutrient Mixture F-12 Ham (DMEM) supplemented with L-glutamine, 15 mM HEPES without phenol red, pH 7.2-7.4 (Sigma-Aldrich, Hamburg, Germany), 100 U/mL penicillin, and 100 µg/mL streptomycin (both from Biochrom, Berlin, Germany). After polymerization of the agarose solution on ice, tissue cores of a diameter of 8 mm were prepared using a sharp rotating metal tube. Subsequently, the cores were sliced into 300-350 µm thin slices in DMEM using a Krumdieck tissue slicer (Alabama Research and Development, Munford, AL). PCLS were washed 3× for 30 min in DMEM and used for experiments. Viability of the tissue was assessed by a LDH Cytotoxicity Detection Kit (Roche, Mannheim, Germany) according to manufacturer's instruction.

For immunofluorescence staining in human PCLS, PCLS from Ctrl patients were fixed with acetone/methanol (Roth) 50:50 by volume for 20 min, blocked for 1 h with 5% bovine serum albumin (w/v, Sigma) in 1x PBS, pH 7.4. Cells were then incubated with primary antibody overnight at 4 °C. After incubation with a secondary antibody for 1 h, nuclei were DAPI stained and PCLS were examined with a confocal microscope (Zeiss). Antibodies used were specific for ITGB2 (1:500 dilution, R&D Systems), and VIM (1:500 dilution, Cell Signaling). Alexa 488, Alexa555 or Alexa 594-tagged secondary antibodies (Invitrogen) were used. DAPI (Sigma Aldrich) used as nuclear dye.

### Statistical Analysis

The source data for all the plots presented in the article, including the values for statistical significance and the implemented statistical tests, are provided in Source Data S1. Further details of statistical analysis in different experiments are included in the Figures and Figure legends. Briefly, expression analysis of samples was analyzed by next generation sequencing in duplicates of one experiment. Three independent experiments of the mass spectrometry-based proteomic approach were performed. For the rest of the experiments presented here, samples were analyzed at least in triplicates and experiments were performed three times. Statistical analysis was performed using Excel Solver and Prism9. Data in bar plots are represented as mean ± standard deviation (mean ± SD). Two-tailed t-tests were used to determine the levels of difference between the groups and *P*-values for significance. *P*-values after two-tailed t-test, * *P* ≤ 0.05; ** *P* < 0.01, and *** *P* < 0.001.

### Data availability

The data that support this study are available from the corresponding author upon reasonable request. In addition, sequencing data of RNA have been deposited in NCBI's Gene Expression Omnibus 94 and is accessible through SRA Sequence Read Archives NCBI with accession number PRJNA835424. The mass spectrometry-based interactome data have been deposited into the PRIDE archive and assigned to the project accession px-submission #576520.

In addition, we retrieved and used a number of publicly available datasets to aid analysis of our data:

Total RNA-seq in NSCLC and SCLC cell lines: European Genome Archive: EGAS00001000610

Total RNA-seq in SCLC patients and cell lines: European Genome Archive: EGAS00001002115, EGAS00001000299

The source data are provided with this paper in the file with the name Source Data S1, Source Data S2 and in the Tables S1 to S7.

## Supplementary Material

Supplementary figures and tables 6-7, legends and explanations for data 1-2, tables 1-5.

Supplementary source data 1.

Supplementary source data 2.

Supplementary table 1.

Supplementary table 2.

Supplementary table 3.

Supplementary table 4.

Supplementary table 5.

## Figures and Tables

**Table 1:** Sequence of primers used for qRT-PCR expression analysis.

Gene	Primer sequence (5'→3')	
*hHPRT*	Forward	TTTGCTTTCCTTGGTCAGGCAGT
	Reverse	CGTGGGGTCCTTTTCACCAGCA
*hGAPDH*	Forward	GGCCCGATTTCTCCTCCGGGT
	Reverse	GGTGACCAGGCGCCCAATACG
*hITGB2*	Forward	TGCGTCCTCTCTCAGGAGTG
	Reverse	GGTCCATGATGTCGTCAGCC
*hmutITGB2*	Forward	CCTGTACTATCTGATGGCCTCTCCTACTCCATG
	Reverse	CATGGAGTAGGAGAGGCCATCAGATAGTACAGG
*hITGB6*	Forward	CCACATGGGGCCTCGCTGTG
	Reverse	CAGTCCAGCCGCTCCTGCAC
*hITGA2*	Forward	TTGGAACGGGACTTTCGCAT
	Reverse	GGTACTTCGGCTTTCTCATCA
*hVIM*	Forward	GGAAATGGCTCGTCACCTTCGT
	Reverse	GCAGAGAAATCCTGCTCTCCTCG
*hCDH1*	Forward	CCCACCACGTACAAGGGTC
	Reverse	ATGCCATCGTTGTTCACTGGA

**Table 2:** Sequence of guide RNAs used for generating *ITGB2* -/- cells.

Name	Sequence (5' → 3')
c794 sg1_ITGB2_FP	CACCGCATCCCCCGGGCCACGGGATT
c795 sg1_ITGB2_RP	AAACAATCCGTGGCCCGGGGGATGC
c796 sg2_ITGB2_FP	CACCGTCTCCCAATCCGTGGCCCG
c797 sg2_ITGB2_RP	AAACCGGGCCACGGATTGGGAGAC
c798 sg3_ITGB2_FP	CACCGTGGAGCCCGCTGCGCGCAG
c799 sg3_ITGB2_RP	AAACCTGCGCGCAGCGGGCTCCATC

## Abbreviations

ATII: alveolar type-II cells
Co-IP: co-immunoprecipitation assay
Ctrl: control
EGF: epidermal growth factor
EGFR: epidermal growth factor receptor
EGFR-TKIs: tyrosine kinase inhibitors targeting epidermal growth factor receptor
EMT: epithelial-mesenchymal transition
ERK1/2: extracellular signal-regulated kinase 1/2
ES: enrichment score
EVs: extracellular vesicles
FDR: false discovery rate
FFPE: formalin-fixed paraffin embedded
GAL3: Galectin-3
GO: gene ontology enrichment analysis
GSEA: gene set enrichment analysis
H3K27me3: histone 3 tri-methylated at lysine 27
hPCLS: human precision-cut lung slices
IQR: interquartile range
ITGA: integrin receptor subunit alpha
ITGB: integrin receptor subunit beta
KEGG: Kyoto Encyclopedia of Genes and Genomes
LC: lung cancer
LOF: loss-of-function
LUAD: lung adenocarcinoma
M: median
MLE-12: mouse lung epithelial cells
mutITGB2: mutant integrin beta 2
NSCLC: non-small cell lung cancer
ORA: over-representation analysis
pEGFR: phosphorylated epidermal growth factor receptor
pMAPK: phosphorylated mitogn-activated protein kinase
RBD: RAS binding domain
RNase: ribonuclease
RNA-seq: RNA-sequencing
RPKM: reads per kilo base million
RTK: receptor tyrosine kinase
SCLC: small cell lung cancer
SD: standard deviation
siRNA: small interfering RNAs
TCGA: the cancer genome atlas
WB: Western Blot analysis
