# Supplementary material for: Non-canonical integrin signaling activates EGFR and RAS-MAPK-ERK signaling in small cell lung cancer
Source: Theranostics. 2023 Apr 17;13(8):2384–407. doi: 10.7150/thno.79493 (PMC10196829; doi:10.7150/thno.79493)
Supplement: Supplementary file 1 — Supplementary figures and tables 6-7, legends and explanations for data 1-2, tables 1-5. [file thnov13p2384s1.pdf]

Supplementary Information for

Non-canonical integrin signaling activates EGFR and RAS-MAPK-ERK signaling in small cell lung cancer

Authors: Karla Rubio[1, 2, 3, 4, *], Addi J. Romero-Olmedo[2, 5], Pouya Sarvari[6], Guruprasadh Swaminathan[1], Vikas P. Ranvir[7], Diana G. Rogel-Ayala[1, 2], Julio Cordero[8, 9], Stefan Günther[10, 11], Aditi Mehta[2, 12], Birgit Bassaly[13], Peter Braubach[14, 15], Malgorzata Wygrecka[16], Stefan Gattenlöhner[13], Achim Tresch[17, 18, 19], Thomas Braun[11], Gergana Dobreva[8, 9], Miguel N. Rivera[3, 20], Indrabahadur Singh[7], Johannes Graumann[21, 22], and Guillermo Barreto[1, 2, 4, *, †]

Correspondence to: guillermo.barreto@univ-lorraine.fr and karla.rubio@univ-lorraine.fr
[†]Lead contact

This PDF file includes:
Figures S1 to S11
Tables S6 and S7

Other Supplementary Materials for this manuscript include the following:
Source Data S1 - This is an Excel file that contains the data for all the plots presented in the article, including the values for statistical significance and the implemented statistical tests.

Source Data S2 - This is a PDF file that contains the data for all the Western Blots presented in the article, including the molecular size markers and the cropped selection.

Table S1 – This is an Excel file that contains the , we identified 50 interaction partners of human ITGA2 with high confidence (combined score ≥ 0.9; 2 nodes), including ITGB2 (combined score = 0.96) and ITGB6 (combined score = 0.97), that we identified using the STRING database.

Table S2 – This is an Excel file that contains the clinical and pathological characteristics of SCLC and Ctrl patients, from which we obtained the formalin-fixed paraffin embedded (FFPE) human lung tissues.

Table S3 - This is an Excel file that contains the clinical and pathological characteristics of LUAD and Ctrl patients, from which we retrieved RNA-seq data from The Cancer Genome Atlas (TCGA).

Table S4 – This is an Excel file that contains the IDs of the transcripts that we suggest as as SCLC-ITGB2 gene expression signature (SCLC-ITGB2-sig).

Table S5 – This is an Excel file that contains the official symbols of the 189 proteins tha we have identified by mass spectrometry analysis of the protein cargo of the isolated EVs from NCI-H196 cells transfected with control plasmid (Ctrl) and from A549 cells transfected either with ITGB2 or mutITGB2 (Figure 5D and Figure S9D)

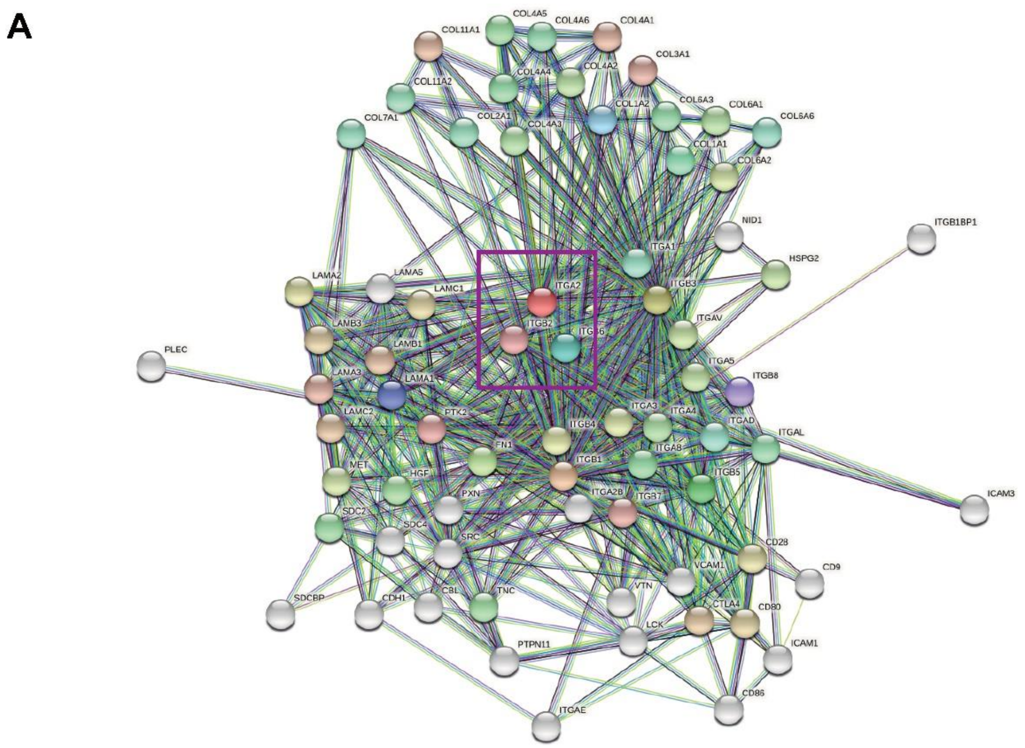

KEGG Pathways

pathway	description	count in network	strength	false discovery rate
hsa04512	ECM-receptor interaction	41 of 88	2.11	7.61e-71
hsa05222	Small cell lung cancer	21 of 92	1.8	3.73e-29
hsa05412	Arrhythmogenic right ventricular cardiomyopathy	17 of 76	1.79	2.30e-23
hsa04510	Focal adhesion	43 of 198	1.78	7.60e-63
hsa05146	Amoebiasis	20 of 100	1.74	9.80e-27

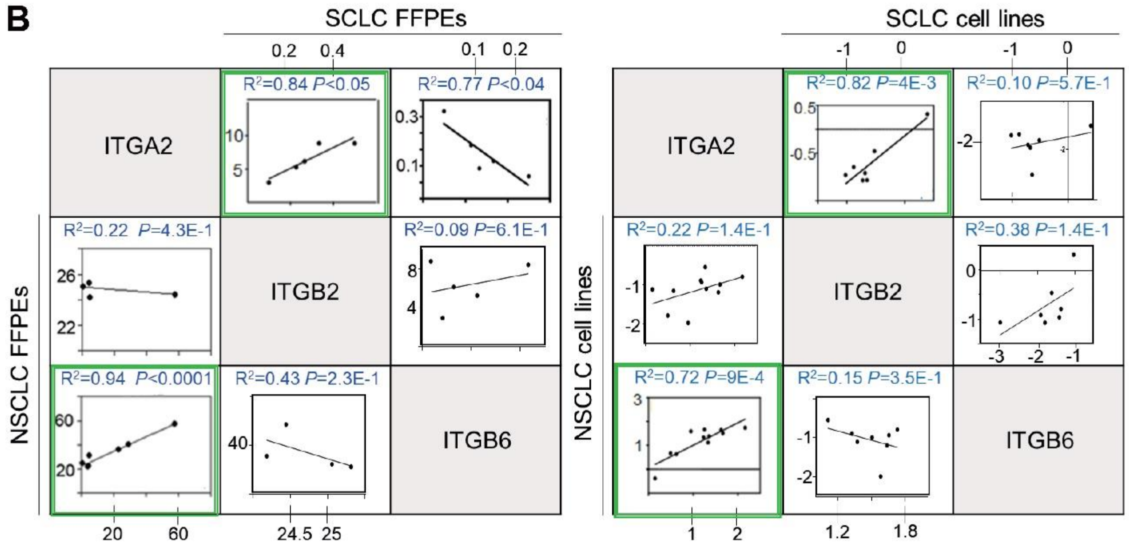

Figure S1: Human ITGA2 interactome is associated to SCLC. (**A**) In silico analysis of human ITGA2 interaction partners using the STRING 10.0 server. Pink box shows ITGA2, ITGB2, and

ITGB6. Line colors indicate the known (turquoise), predicted (green), gene fusion (red), gene co-occurrence (blue) or experimental (purple) interactions. KEGG-based enrichment analysis of significant pathways including the ITGA2 interactome shows a significant enrichment with SCLC pathways. (**B**) Correlation analysis between *ITGA2*, *ITGB2* and *ITGB6* by linear regression of relative normalized expression in FFPE lung tissue sections from NSCLC and SCLC patients. (**C**) Correlation analysis between *ITGA2*, *ITGB2* and *ITGB6* by linear regression of relative normalized expression in NSCLC and SCLC cells lines.

A

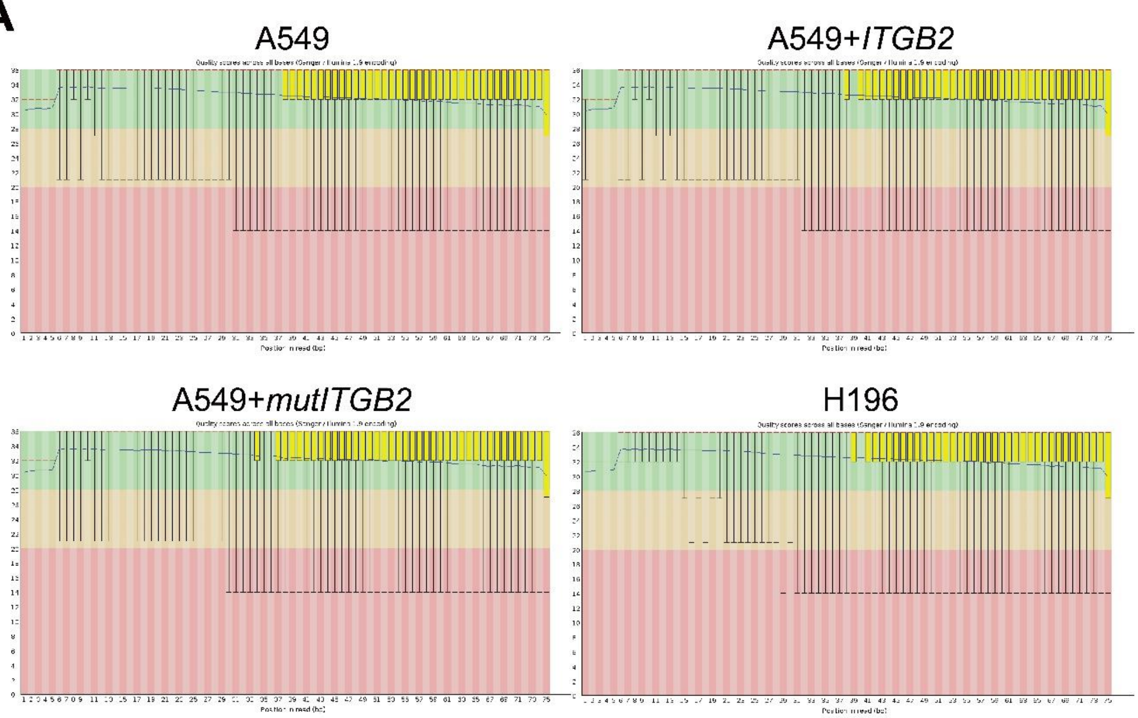

B

Sample	No. of reads	Surviving reads	Mapped reads
**A549**	28053330	28005152 (99.83%)	27373180 (97.74)
**A549+ITGB2**	33126092	33071485 (98.84%)	32321730 (97.73%)
**A549+mutITGB2**	38002078	37932995 (99.82%)	37091007 (97.78%)
**H196**	31993308	31942139 (99.84%)	31159412 (97.55%)

Figure S2: Transcriptomic analysis of SCLC and NSCLC ITGB2-overexpressing cells. (**A**) RNA-sequencing using SCLC (NCI-H196) and NSCLC (A549) cell lines without our with the overexpression of *ITGB2/mutITGB2* (accessible through the GEO BioProject ID PRJNA835424). Phred quality score distribution over all reads in each base. The score is divided into very good quality calls (green), calls of reasonable quality (orange), and calls of poor quality (red). (**B**) Description of the RNA-seq data sets supports the quality of the experiment.

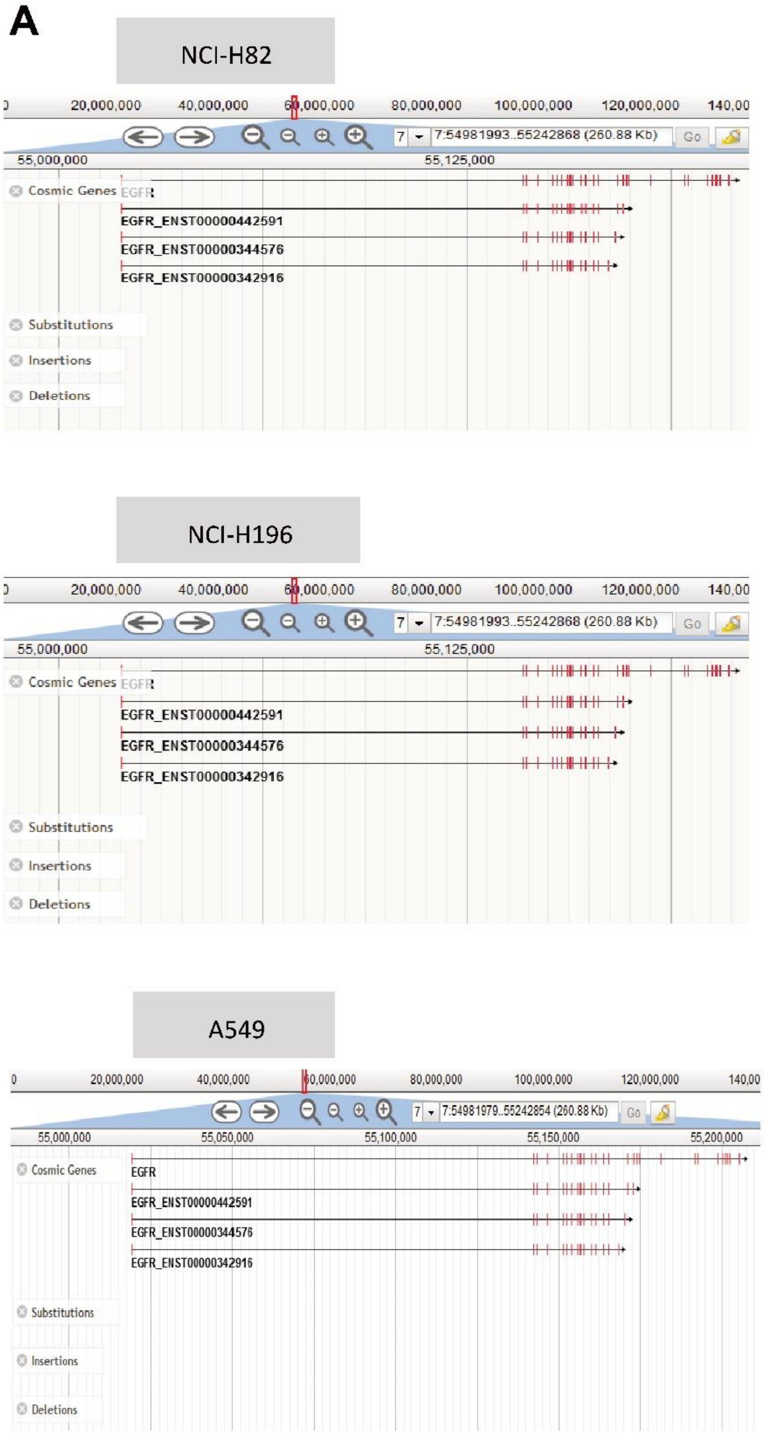

Figure S3: EGFR locus in NSCLC and SCLC cells. (**A**) Somatic mutations absence in the EGFR locus in NCI-H82, NCI-H196 and A549 cell lines from the Catalogue of Somatic Mutations in Cancer (COSMIC) is depicted. Mutation data were obtained from COSMIC v77 at the Welcome

Trust Sanger Institute (Cambridge, UK). Only the frequency of somatic mutations (single nucleotides, or small insertions or deletions (indels)), but not larger deletions, amplifications or rearrangements, are considered.

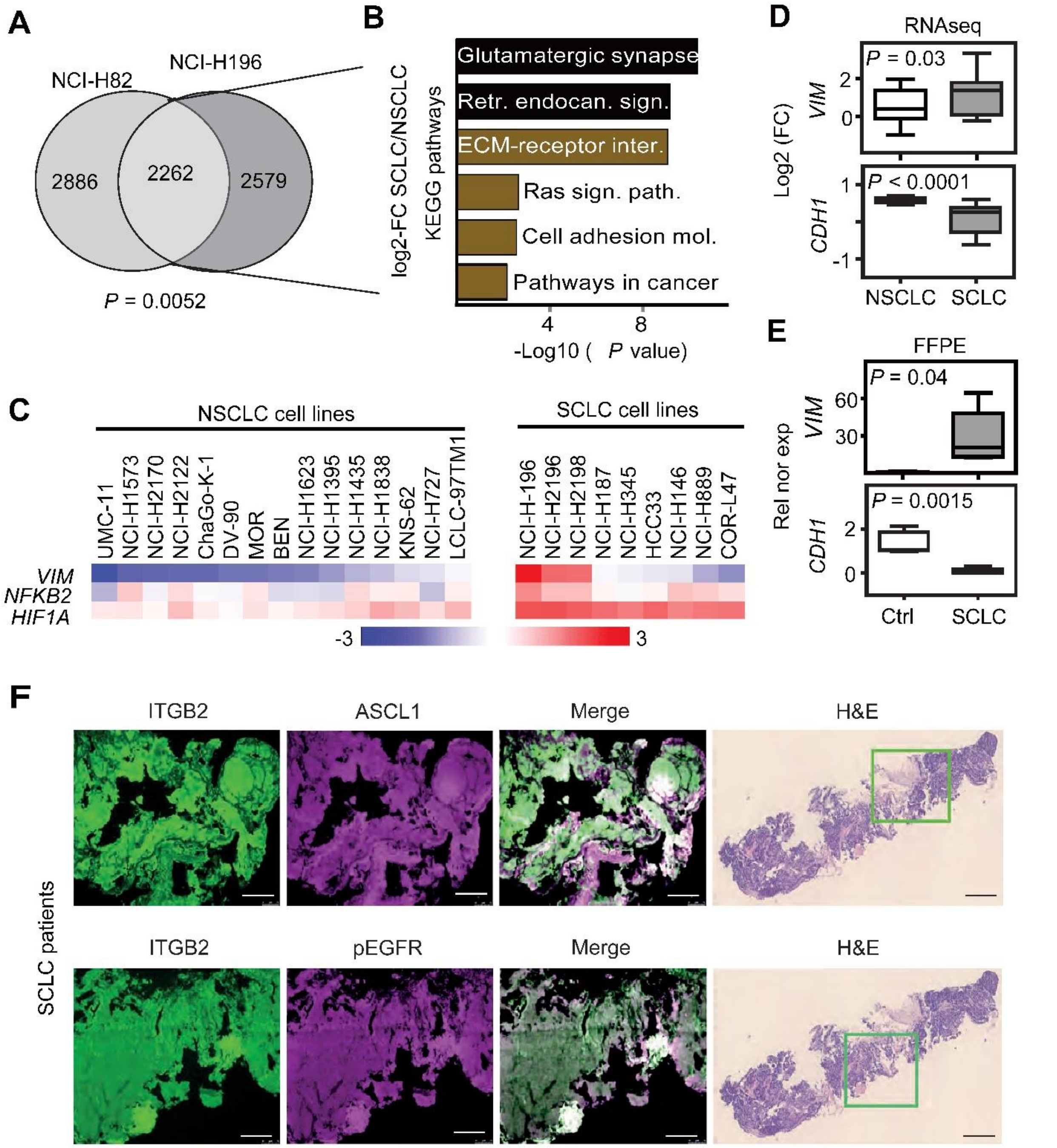

Figure S4: Active EGF signaling and pro-oncogenic pathways are predominant in ITGB2-expressing SCLC cell lines and lung tissue from SCLC patients. (**A**) Venn diagram displaying overlap of common, highly expressed genes in NCI-H82 and NCI-H196 cells when compared to

A549 cells. *P*-value after Fisher´s Exact test. (**B**) KEGG-based enrichment analysis of the common, highly expressed genes in NCI-H82 and NCI-H196 cells using DAVID bioinformatics tool and plotted by highest significance (-log10 of the modified Fisher exact *P*-value). Retr., retrograde; endocan., endocannabinoid; sign., signaling; path., pathway; mol. molecules. (**C**) Heatmaps of *VIM*, *NFKB2* and *HIF1A* expression in NSCLC and SCLC cancer cell lines. Hierarchical clustering was performed using Person's correlation-based distance and average linkage. (**D**) Box plots of RNA-seq-based expression analysis of indicated transcripts in non-small cell lung cancer (NSCLC; $n = 33$) and small cell lung cancer (SCLC; $n = 17$) cell lines. Values are represented as log2 fold change (FC). (**E**) Box plots of qRT-PCR-based expression analysis of indicated transcripts using RNA isolated from FFPE lung tissue sections from Ctrl ($n = 4$) and small cell-lung cancer (SCLC, $n = 5$) patients. Rel nor exp, relative normalized expression to *GAPDH*. All box plots (D-E) indicate median (middle line), 25th, 75th percentile (box) and 5th and 95th percentile (whiskers); P-values after two-tailed t-test. Source data are provided as Source Data S1. (**F**) Left, fluorescence microscopy after immunostaining using ITGB2, ASH1 or pEGFR-specific antibodies in human lung tissue from SCLC patients. Right, hematoxylin and eosin staining (H&E) in human lung tissue from SCLC patients. Squares are respectively shown in left part of the panel at higher magnification. Scale bars, 150 µm (left) and 500 µm (right).

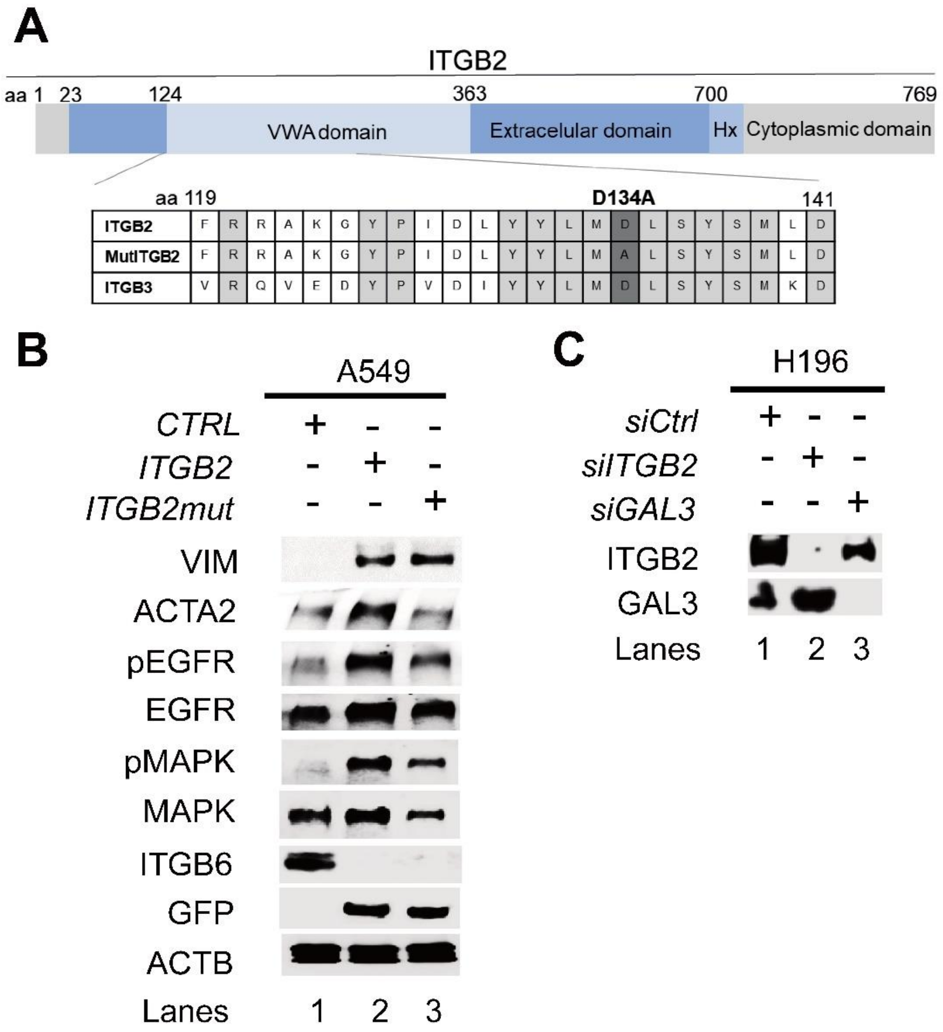

Figure S5: Non-canonical ITGB2 signaling activates EGFR. (**A**) Top, representation of ITGB2 functional domains. The numbers indicate amino acids (aa) positions. VWA, von Willebrand factor type A domain. Bottom, amino acid sequence alignment between ITGB2, mutITGB2 and ITGB3 in a part of the VWA domain highlighting the amino acid position 134, in which the point mutation D134A was incorporated to generate the ligand-binding-deficient ITGB2 mutant following a similar strategy as previously published for ITGB3 [PMID: 24747441; 28860622]. (**B**) Total protein extracts of A549 cells transfected with *ITGB2* or *mutITGB2* were analyzed by WB

using the indicated antibodies. (**C**) Total protein extracts from NCI-H196 cells that were transfected with Ctrl, ITGB2- or GAL3-specific small interfering RNAs (*siCtrl*, *siITGB2* or *siGAL3*) were analyzed by WB using the indicated antibodies.

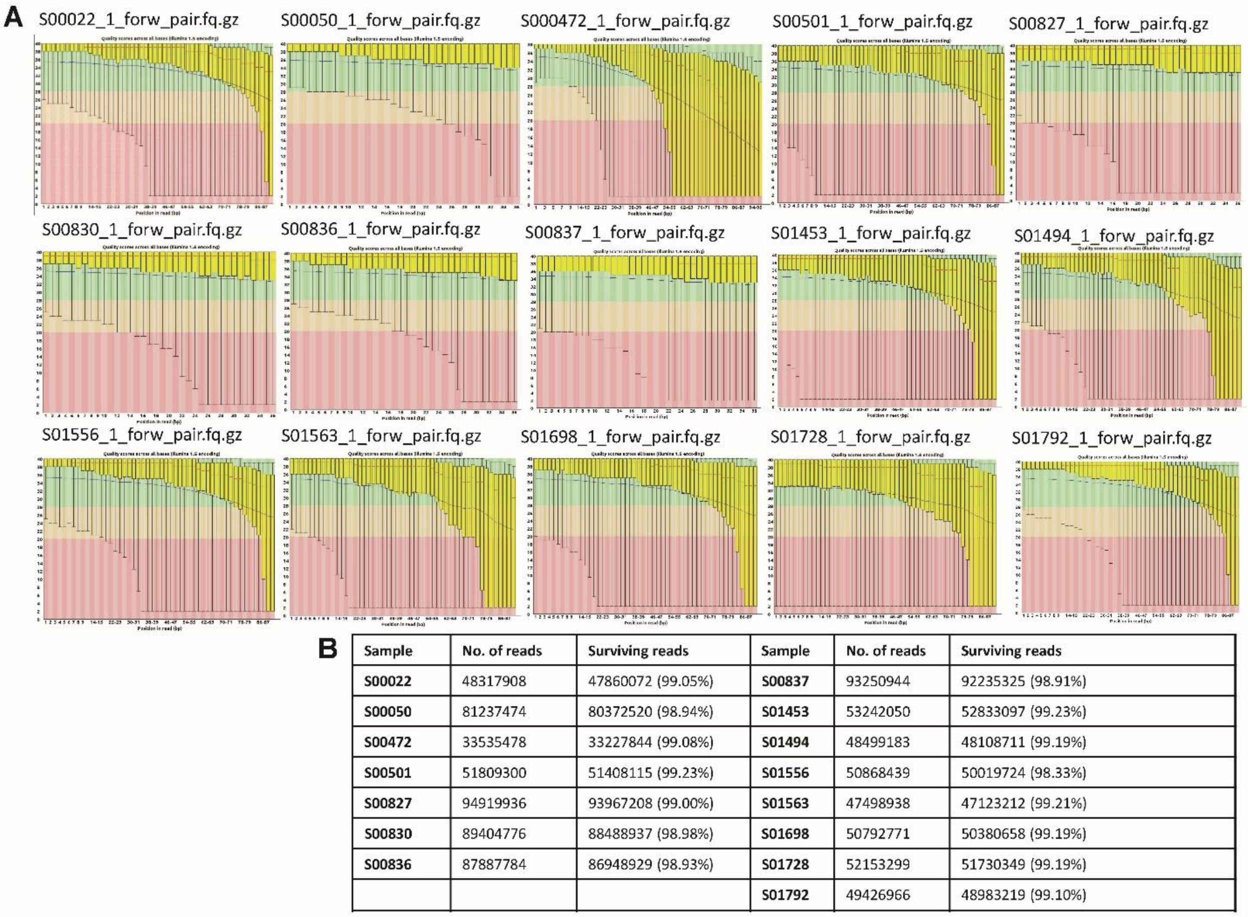

B

Sample	No. of reads	Surviving reads	Sample	No. of reads	Surviving reads
**S00022**	48317908	47860072 (99.05%)	**S00837**	93250944	92235325 (98.91%)
**S00050**	81237474	80372520 (98.94%)	**S01453**	53242050	52833097 (99.23%)
**S00472**	33535478	33227844 (99.08%)	**S01494**	48499183	48108711 (99.19%)
**S00501**	51809300	51408115 (99.23%)	**S01556**	50868439	50019724 (98.33%)
**S00827**	94919936	93967208 (99.00%)	**S01563**	47498938	47123212 (99.21%)
**S00830**	89404776	88488937 (98.98%)	**S01698**	50792771	50380658 (99.19%)
**S00836**	87887784	86948929 (98.93%)	**S01728**	52153299	51730349 (99.19%)
			**S01792**	49426966	48983219 (99.10%)

Figure S6: Transcriptomic analysis of SCLC patients tissue. (**A**), RNA-sequencing using lung tissue from SCLC patients (accessible through the European Genome Archive with accession number EGAS0000100299). Phred quality score distribution over all reads in each base. The score is divided into very good quality calls (green), calls of reasonable quality (orange), and calls of poor quality (red). (**B**), Description of the RNA-seq data sets supports the quality of the experiment.

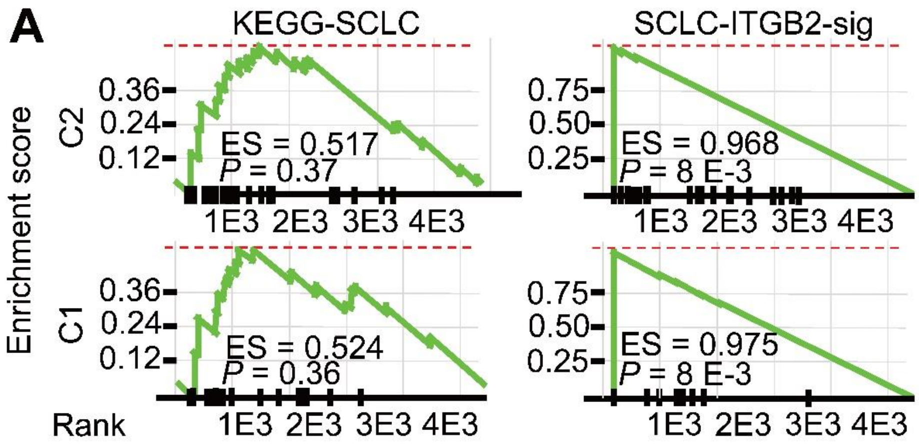

Figure S7: Confirmation of the SCLC-ITGB2 gene expression signature (SCLC-ITGB2-sig) using RNA-seq data from SCLC patients. Gene Set Enrichment Analysis (GSEA) using RNA-seq data from SCLC patients in cluster 1 (C1) and cluster 2 (C2) from Figure 2A comparing the conventional SCLC signature in KEGG (left) versus the SCLC-ITGB2-sig (right) identified in Figure 2E. ES, enrichment score; P-value after two-tailed t-test.

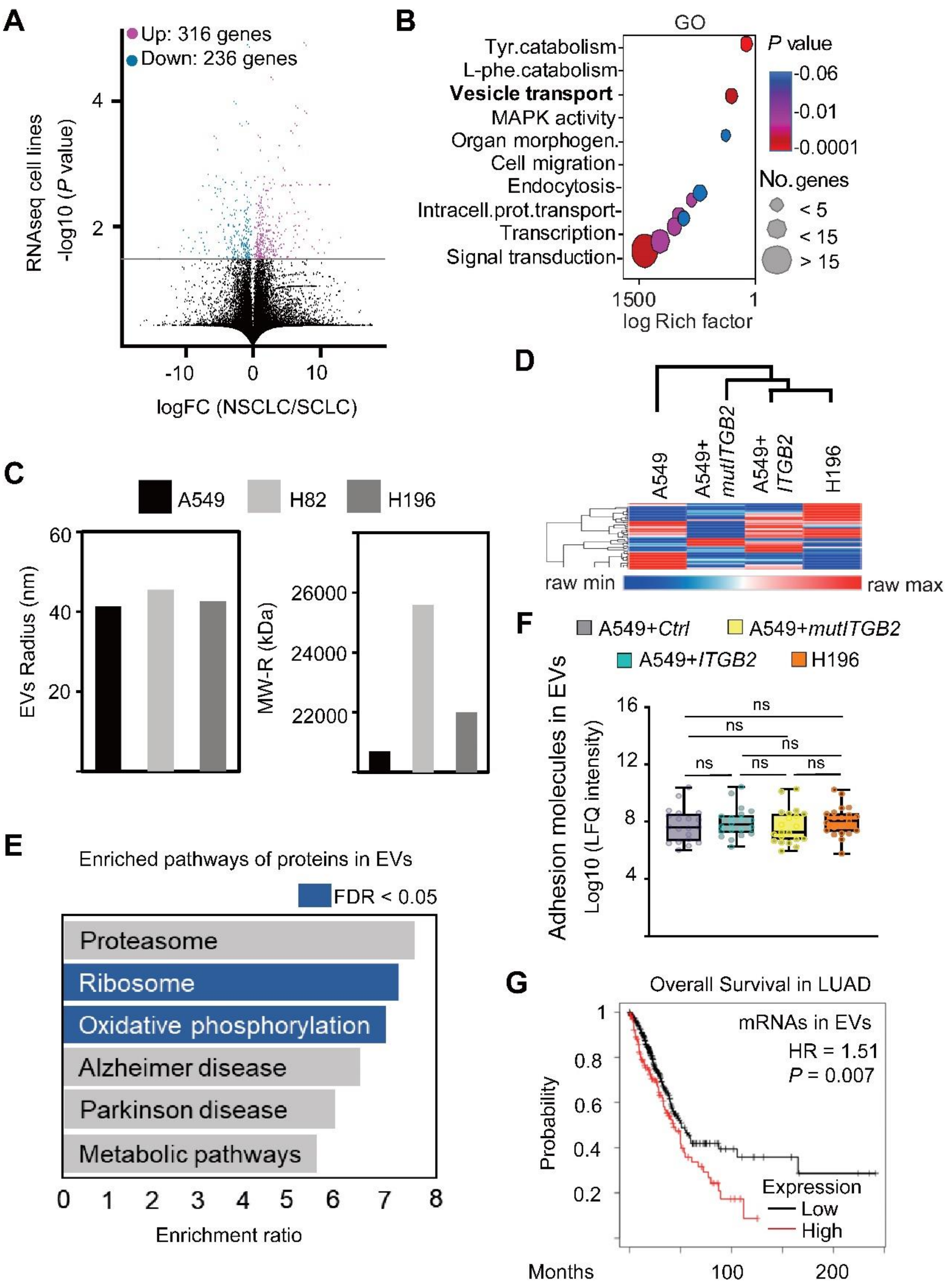

Figure S8: Extracellular vesicles containing ITGB2 are secreted from SCLC cells and ITGB2-transfected NSCLC cells. (**A**) Volcano plot representing the significance (-log10 *P*-

values after two-tailed Welch´s t-Test) versus expression fold change (log2 expression ratios) between NSCLC and SCLC cells. Magenta dots show significantly upregulated transcripts, blue dots show significantly downregulated transcripts. (**B**) Gene Ontology-based enrichment analysis of up-regulated transcripts in SCLC using Webgestalt bioinformatics tool and plotted by highest significance (log Rich factor). Tyr., tyrosine; phe., phenylalanine; intracell., intracelular; prot., protein. (**C**) Radius (nm) and molecular weight (kDa) of extracellular vesicles (EVs) isolated from the culture medium of A549, NCI-H82 and NCI-H196 cells. Differential Light Scattering (DLS) was used to determine the EVs size. (**D**) Heatmap showing a hierarchical clustering from secreted EVs cargo proteins detected by Mass Spectrometry in supernatants of A549, A549+*ITGB2*, A549+*mutITGB2* and NCI-H196 cells. The complete proteomics data was submitted in the PRIDE repository with the accession number PX576520. (**E**) KEGG -based pathway enrichment analysis of the 189 common proteins detected by HRMS in EVs using the WebGestalt toolkit and plotted by highest significance (log Rich factor). (**F**) Box plots indicating the protein levels of cell adhesion molecules (CAMs) identified by mass spectrometry in the protein cargo of EVs that were isolated from non-transfected NCI-H196 cells (n = 21) or A549 cells transfected with control plasmid (*Ctrl*, n = 20) or expression constructs for wild-type *ITGB2* (n = 21) or mutated *ITGB2* (*mutITGB2*; n = 20). Values are represented as log10 LFQ. Box plots indicate median (middle line), 25th, 75th percentile (box) and 5th and 95th percentile (whiskers); P-values after two-tailed t-test; ns, not significant. (**G**) Overall survival rates by Kaplan-Meier plotter of LUAD patients expressing low (n = 441) or high (n = 160) of 189 common proteins (50.93 vs 42.93 months, respectively, P = 0.007 after Log Rank test). HR, hazard ratio.

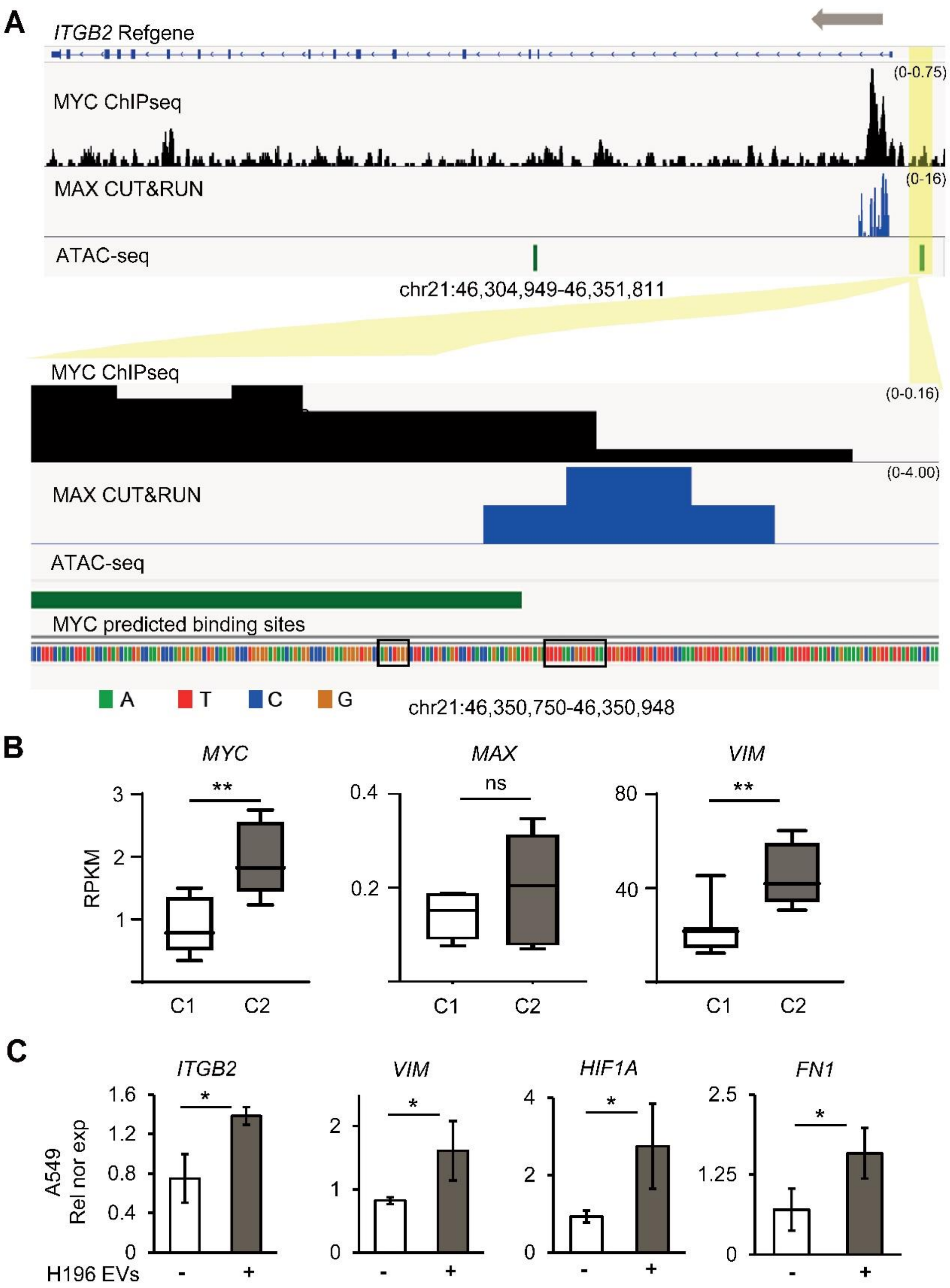

Figure S9: ITGB2 expression appears to be mediated by MYC-MAX heterodimer. (**A**) Upper panel: Visualization of ITGB2 locus showing enrichment of MYC (black) and MAX (blue) in the 5’ end of the gene. ATAC-seq (green) showing open chromatin in a region upstream the

transcriptional start site. Lower panel: Zoom in to the region showing open chromatin, also showing MYC and MAX enrichment consistent with the MYC predicted binding sites (black squares). All datasets were downloaded from GEO repository (GSM3073949, GSM6222857 and GSM4729164). The binding sites were predicted using ConTra3 [PMID: 28472390]. (**B**) Box plots of RNA-seq-based expression analysis of *MYC*, *MAX* and *VIM* in SCLC patients C1 and C2. Values are represented as RPKM. Box plots indicate median (middle line), 25th, 75th percentile (box) and 5th and 95th percentile (whiskers); *P*-values after two-tailed t-test; **$P<0.01$; ns, not significant. Source data are provided as Source Data S1. (**C**) qRT-PCR-based expression analysis of *ITGB2* in A549 cells non-treated (-) or incubated with EVs isolated from NCI-H196 cells. Rel nor exp, relative normalized expression to *GAPDH*. Data are shown as means ± SD (n = 3); asterisks, *P*-values after two-tailed t-test, * $P < 0.05$. Source data are provided as Source Data S1.

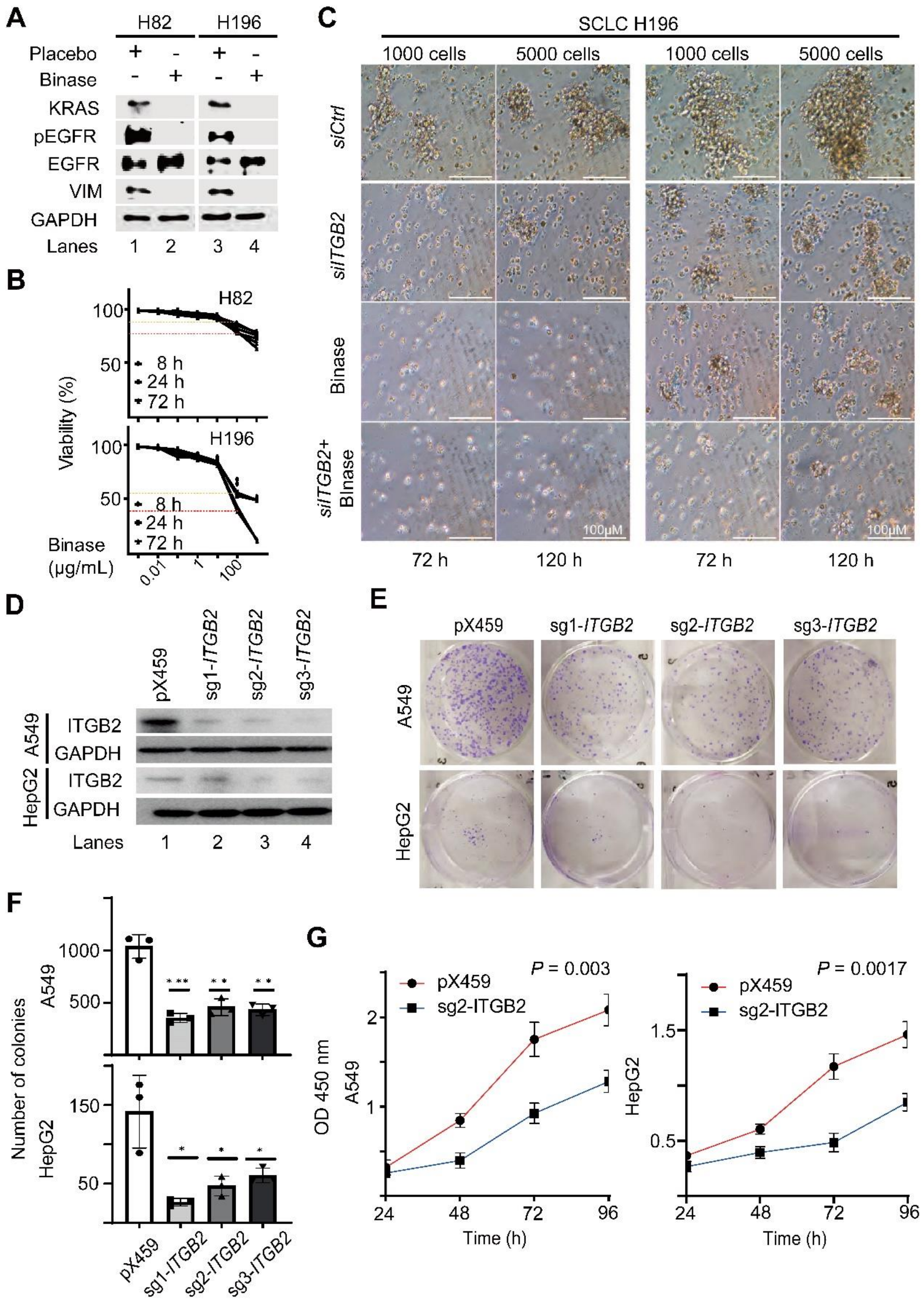

Figure S10: ITGB2 loss-of-function and binase reduce cancer hallmarks. (**A**) Total protein extracts of NCI-H82 and NCI-H196 cells treated with Placebo or with binase analyzed by WB

using the indicated antibodies. (**B**) NCI-H82 and NCI-H196 cells were treated with increasing concentrations of binase for 8, 24 and 72h. Cell viability was determined using the BrdU incorporation fluorimetric assay. (**C**) Representative morphology of cell aggregates from NCI-H196 cells transfected with *siCtrl* or *siITGB2*, alone or in combination with binase treatment. The images represent the change of morphology of cell aggregates after aggregate formation for 72h and 120h with initial 1000 or 5000 cells. (**D**) CRISPR-Cas9 mediated loss of *ITGB2* in A549 and HepG2 cells. Protein extracts of A549 (top) and HepG2 cells (bottom) were analyzed by WB using the indicated antibodies. The cells were transfected with control construct (*pX459*) or three different guide RNAs targeting the ITGB2 locus (*sg1-ITGB2*, *sg2-ITGB2* and *sg3-ITGB3*). (**E-F**) CRISPR-Cas9 mediated loss of *ITGB2* reduced colony formation ability in A549 and HepG2 cells. Representative pictures (E) and quantification of three independent experiments (F). In the bar plots, data are shown as means ± SD (n = 3); asterisks, P-values after one-tailed t-test, *** $P < 0.001$; ** $P < 0.01$. (**G**) CRISPR-Cas9 mediated loss of *ITGB2* reduced cell proliferation in A549 and HepG2 cells as measured by CCK-8 assay. data are shown as means ± SD (n = 3). P-values were calculated after one-tailed t-test. Source data for all plots are provided as Source Data S1.

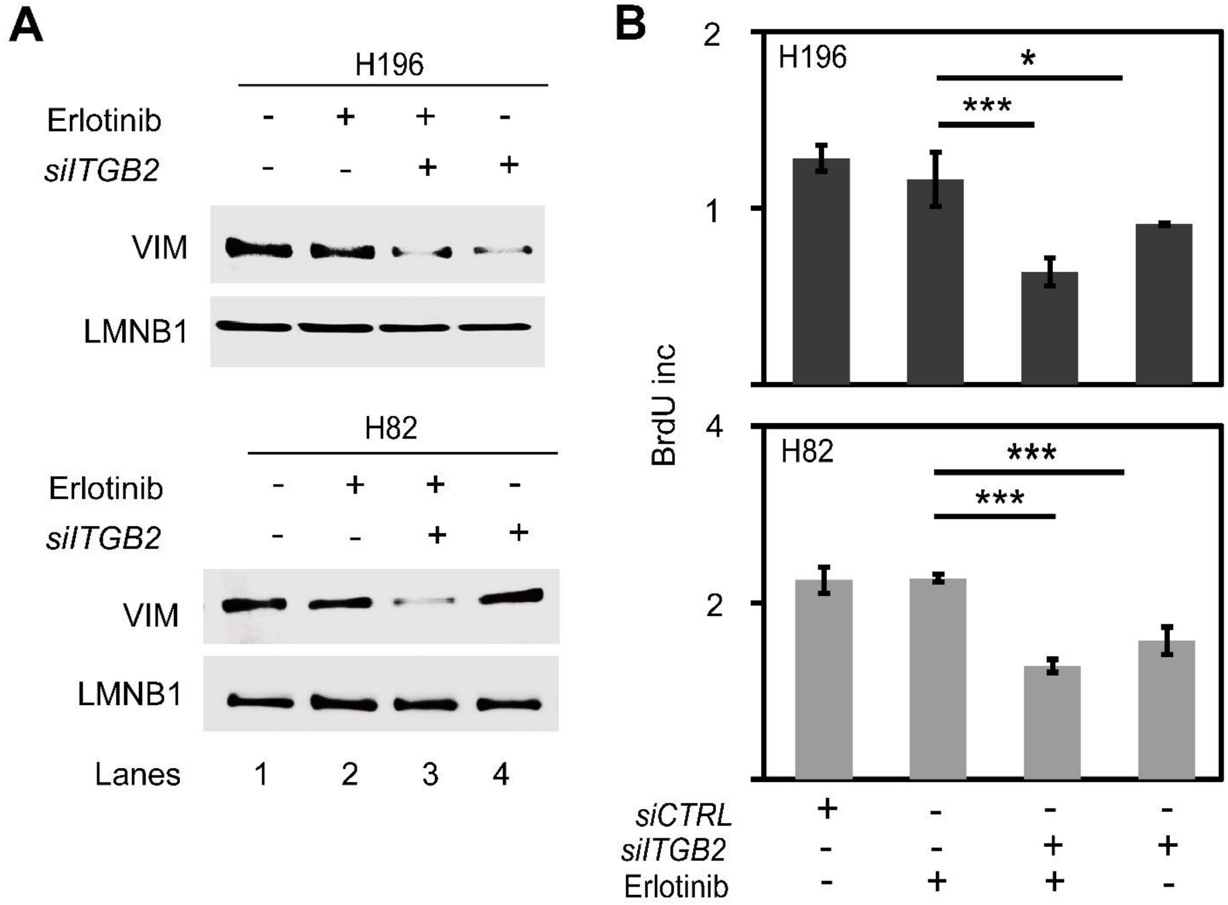

Figure S11: ITGB2 loss-of-function sensitizes SCLC cells to EGFR inhibitor. (**A**) Total protein extracts of NCI-H82 and NCI- H196 cells treated with 200 nM Erlotinib or transfected with *siCtrl* or *siITGB2* were analyzed by WB using the indicated antibodies. (**B**) Proliferation activity of NCI-H82 (left) or NCI-H196 (right) cells treated with 200 nM Erlotinib or transfected with *siCtrl* or *siITGB2* was measured by the colorimetric method using BrdU incorporation. Data are shown as means ± SD ($n = 3$); asterisks, P values after t-Test, *** $P < 0.001$; * $P < 0.05$.

Table S6: Summary of pre-clinical and clinical studies, in which binase and other RNases have been tested in the context of different cancer types.

Tumor	Ribonuclease sensitivity	Compound	Model	PMID
Lung				
NSCLC	Yes	Binase	*In vivo*	23759588
NSCLC	Yes	Binase	*In vivo*	24565811
NSCLC	Yes	RNase A	*In vivo*	29108266
NSCLC	Yes	Binase	*In vitro*	22856132
NSCLC	Yes	Binase	*In vitro*	23567038
NSCLC	Yes	Onconase	*In vitro, In vivo*	27590062
NSCLC	Yes	Onconase	*In vivo*	17352247
NSCLC	Yes	OnconaseR	*In vitro*	1503903
NSCLC	Yes	Binase+ Bleomycin	*In vitro*	27420619
Mesothelioma	Yes	Onconase	*In vitro*	27590062
Colon	Yes	Binase	*In vitro*	28955235
Colon	Yes	Binase	*In vitro, In vivo*	16586499
Pancreatic	Yes	OnconaseR	*In vitro*	1503903
Stomach	Yes	Binase	*In vitro*	32163398
Cervical	Yes	Binase	*In vitro*	33375305
Cervical	Yes	Binase	*In vitro*	29069817
Leukemia	Yes	Binase	*In vitro*	36165015
Leukemia	Yes	Binase	*In vitro*	27239856
Leukemia	Yes	RNase Sa	*In vitro*	12228255
Lymphosarcoma	Yes	Binase	*In vivo*	23759588
Lymphosarcoma	Yes	Binase	*In vivo, In vitro*	33147876
Ovarian	Yes	Binase	*In vitro*	25301481
Ovarian	Yes	T2 RNase	*In vivo*	23630276
Ovarian	Yes	T2 RNase	*In vitro*	16586499
Breast	Yes	Binase	*In vitro*	33375305
Breast	Yes	Binase	*In vitro*	16586499
Breast	Yes	RNAase A	*In vitro*	26502078
Melanoma	Yes	Binase	*In vivo*	23759588

Tumor	Clinical trial	Drug	PMID/identifier
Mesothelioma	Phase IIIb	Doxorubicin+Ranpirnase	NCT00003034
Renal	Phase II	Ranpirnase (Onconase®)	11561684

Table S7: Tissue and cell lines from SCLC exhibit EGFR inhibitor resistance. Tyrosine kinase-inhibitors (TKI) sensitivity for patient derived NSCLC and SCLC tissues and cell lines.

Lung tumor	EGFR inhibitors-sensitivity	Drug	PMID
NSCLC patients			
	Yes	Erlotinib†	28772281
	Yes	Erlotinib†	28761738
	Yes	Erlotinib†	28503070
	Yes	Erlotinib†	28408243
	Yes	Erlotinib†, Tivantinib[i]	27843623
	Yes	Erlotinib†	25758528
	No (T790M mutation)	Erlotinib†, Gefitinib‡	28854272
	No (T790M mutation)	Erlotinib†, Afatinib*	28838400
	No (T790M mutation)	Erlotinib†	28778263
NSCLC cell lines			
A549	Yes	Erlotinib†	16014882
NCI-H3255	Yes	Erlotinib†	17493170
PC9	Yes	Erlotinib†	17493170
NCI-H2170	Yes	Erlotinib†	17493170
NCI-H2073	Yes	Erlotinib†	17493170
SCLC patients			
	No	Erlotinib†	25758528
	No	Erlotinib†	25700797
	No	Erlotinib†	24828667
SCLC cell lines			
NCI-H82	No	Erlotinib†	25758528
NCI-H196	No	Erlotinib†	25758528
HCC4006	No	Erlotinib†	21597390

NCI-H1975	No	Erlotinib†	17493170
NCI-H460	No	Erlotinib†	17493170
†*Erlotinib* (Tarceva, CAS 183321-74-6, Genentech, OSI Pharmaceuticals, Roche)			
[i]*Tivantinib* (ARQ197, CAS 905854-02-6, Arqule Inc.)			
‡*Gefitinb* (Iresssa, CAS 184475-35-2, AstraZeneca)			
Afatinib (Gilotrif, CAS 850140-72-6, Boehringer Ingelheim)			

Data S1. (separate files)

Source Data S1 - This is an Excel file that contains the data for all the plots presented in the article, including the values for statistical significance and the implemented statistical tests.

Source Data S2 - This is a PDF file that contains the data for all the Western Blots presented in the article, including the molecular size markers and the cropped selection.

Tables S1 – This is an Excel file that contains the 50 interaction partners of human ITGA2 with high confidence (combined score ≥ 0.9; 2 nodes), including ITGB2 (combined score = 0.96) and ITGB6 (combined score = 0.97), that we identified using the STRING database.

Table S2 – This is an Excel file that contains the clinical and pathological characteristics of SCLC and Ctrl patients, from which we obtained the formalin-fixed paraffin embedded (FFPE) human lung tissues.

Table S3 - This is an Excel file that contains the clinical and pathological characteristics of LUAD and Ctrl patients, from which we retrieved RNA-seq data from The Cancer Genome Atlas (TCGA).

Table S4 – This is an Excel file that contains the IDs of the transcripts that we suggest as SCLC-ITGB2 gene expression signature (SCLC-ITGB2-sig).

Table S5 – This is an Excel file that contains the official symbols of the 189 proteins that we have identified by mass spectrometry analysis of the protein cargo of the isolated EVs from NCI-H196 cells transfected with control plasmid (*Ctrl*) and from A549 cells transfected either with *ITGB2* or *mutITGB2* (Figure 5D and Figure S9D)
