# Supplementary material for: Non-canonical integrin signaling activates EGFR and RAS-MAPK-ERK signaling in small cell lung cancer
Source: Theranostics. 2023 Apr 17;13(8):2384–407. doi: 10.7150/thno.79493 (PMC10196829; doi:10.7150/thno.79493)

Fig 1A. WB of an IP assay using MLE-12 cells co-transfected with ITGA2-HIS and ITGB2-YFP or ITGA2-HIS and ITGB6-GFP

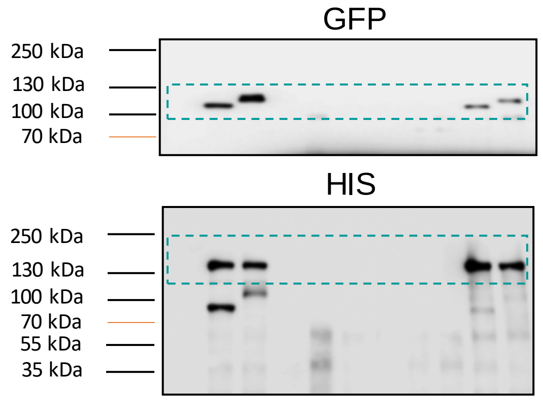

Fig 2A. WB of total protein extracts of A549, NCI-H82 and NCI-H196 cell lines transfected with ITGB2 or ITGB6

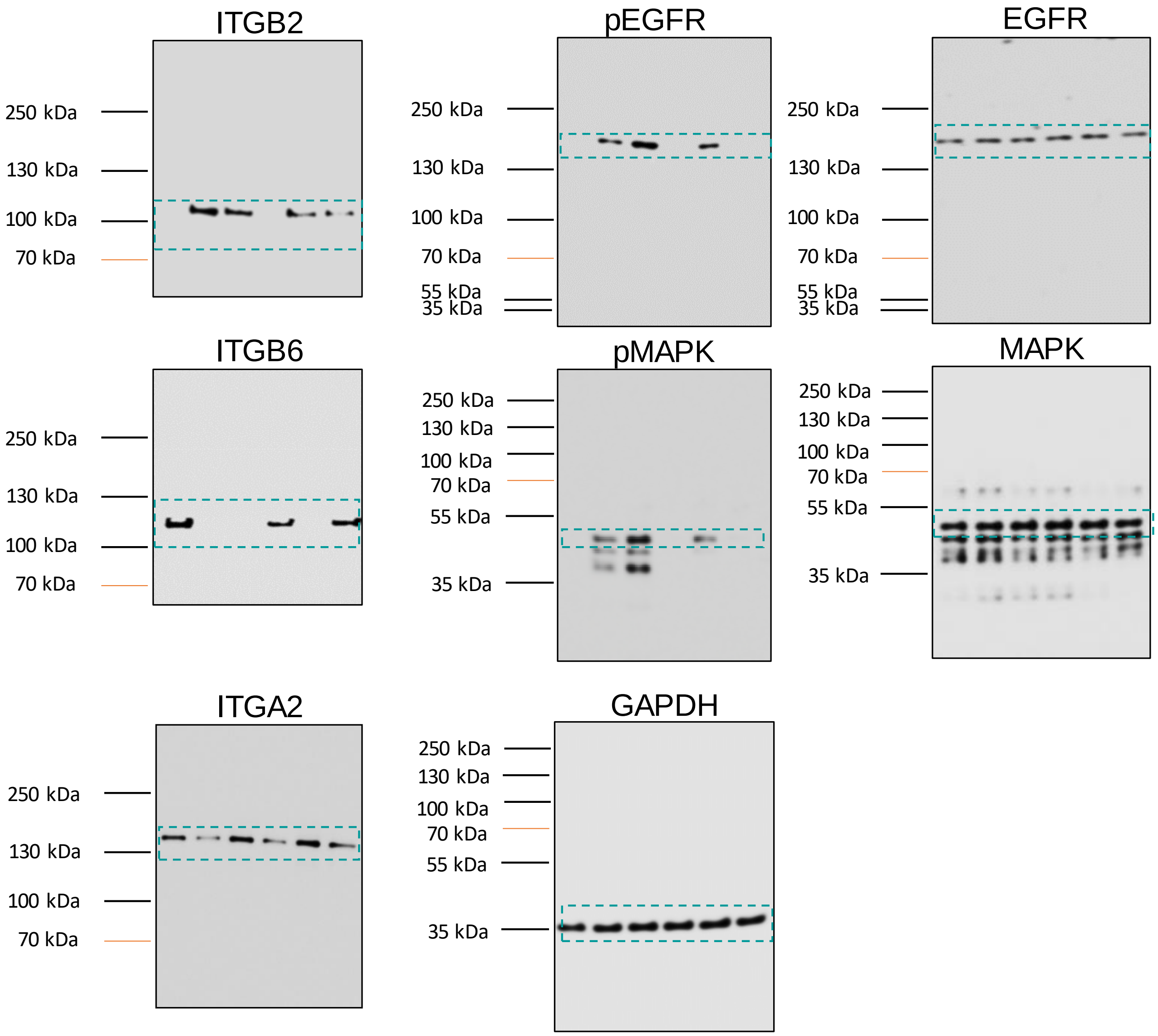

Fig 2B. WB of total protein extracts of A549 cells transfected with ITGB2, mutITGB2 or siGAL3

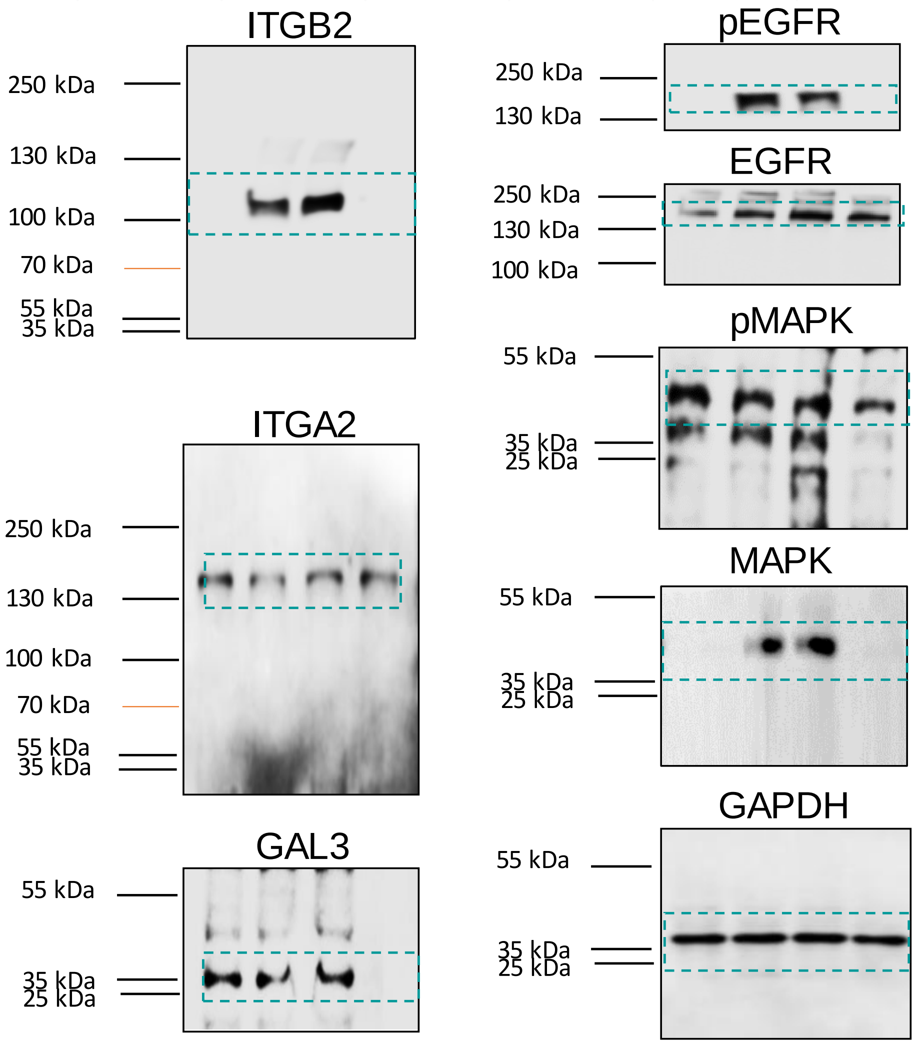

Fig 2E. Total protein extracts of NCI-H196 cell lines transiently transfected with Ctrl, or MYC-tagged EGFR-WT or EGFR mutants

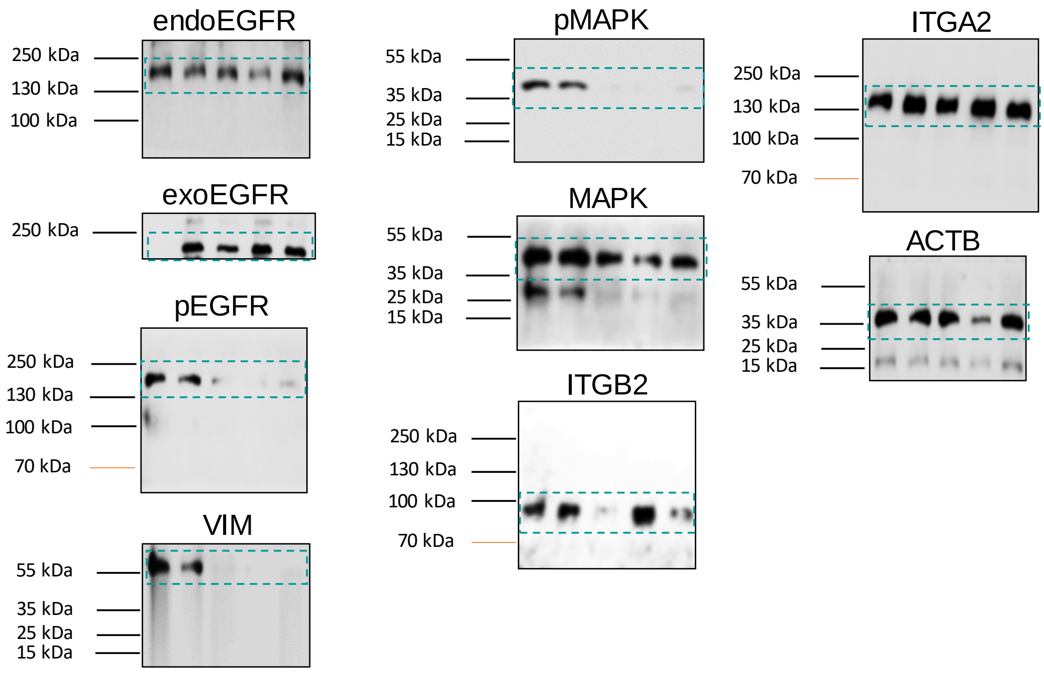

Fig 5C. WB of protein extracts of A549, NCI-H196 and NCI-H82 after IP using a KRAS-specific antibody or RAF-RBD coated beads

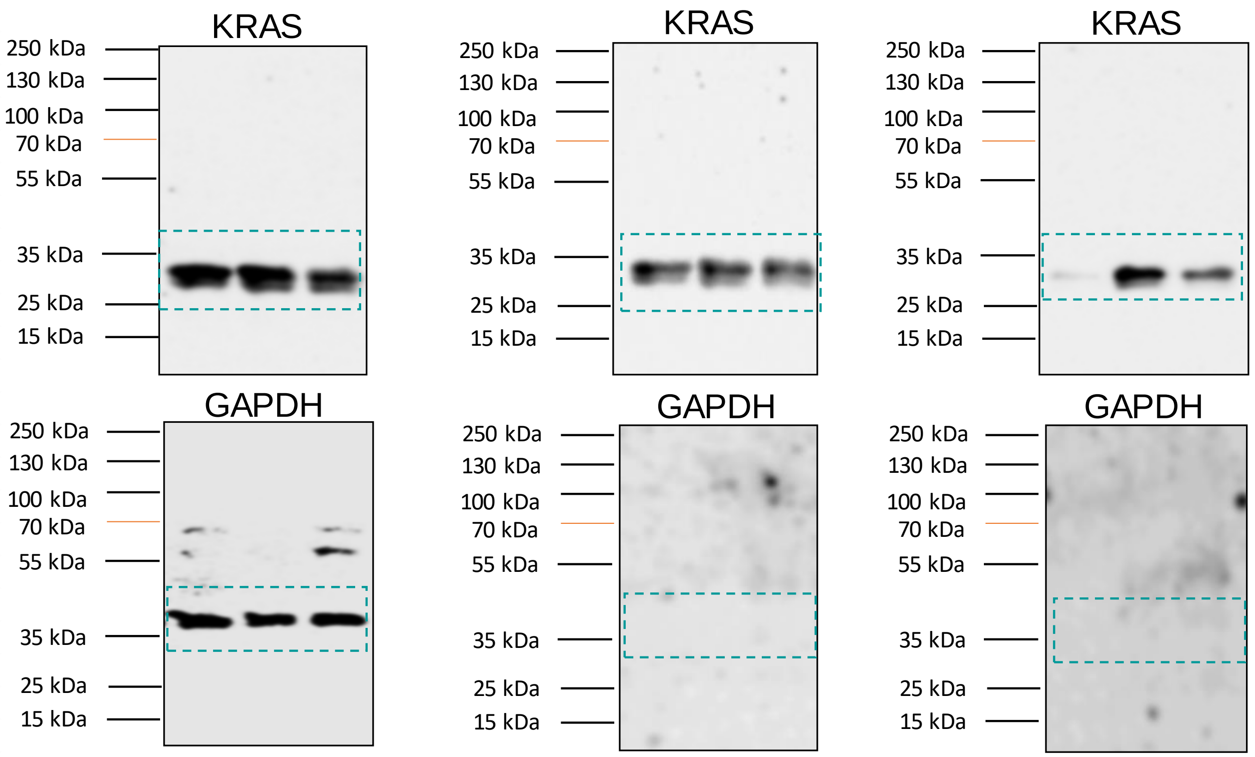

Fig 5D. WB of total protein extracts of A549 cells transfected with ITGB2 or mutITGB2

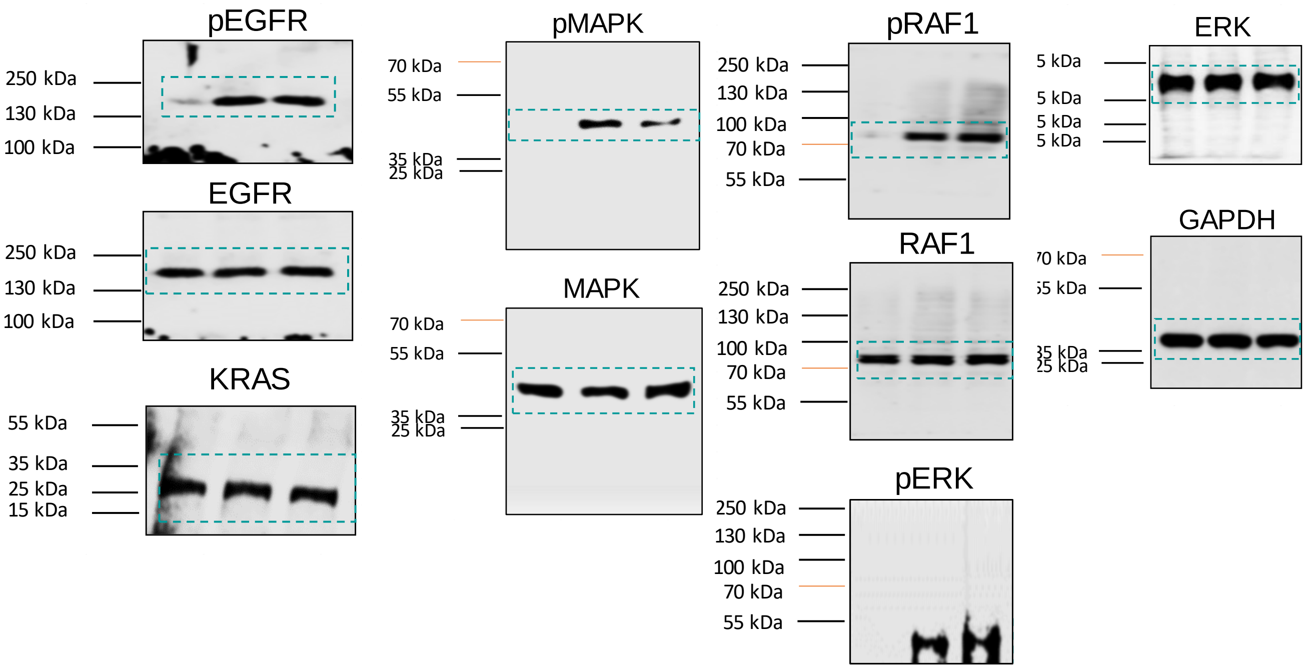

Fig 5E. WB of total protein extracts of NCI-H82 and NCI-H196 cells transfected with *siITGB2* or *siKRAS*

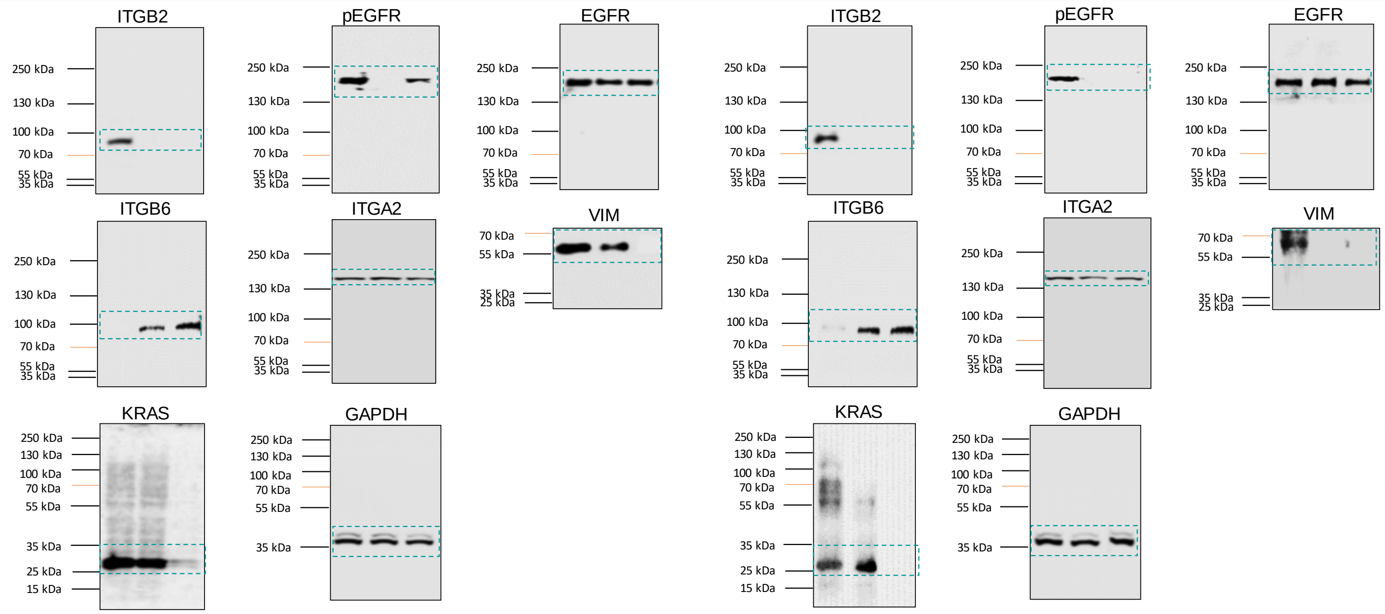

Fig 6A. WB of total protein extracts of A549 cells transfected with Ctrl or ITGB2, and immunoprecipitated using either IgG control or ITGB6 and ITGB2-specific antibodies

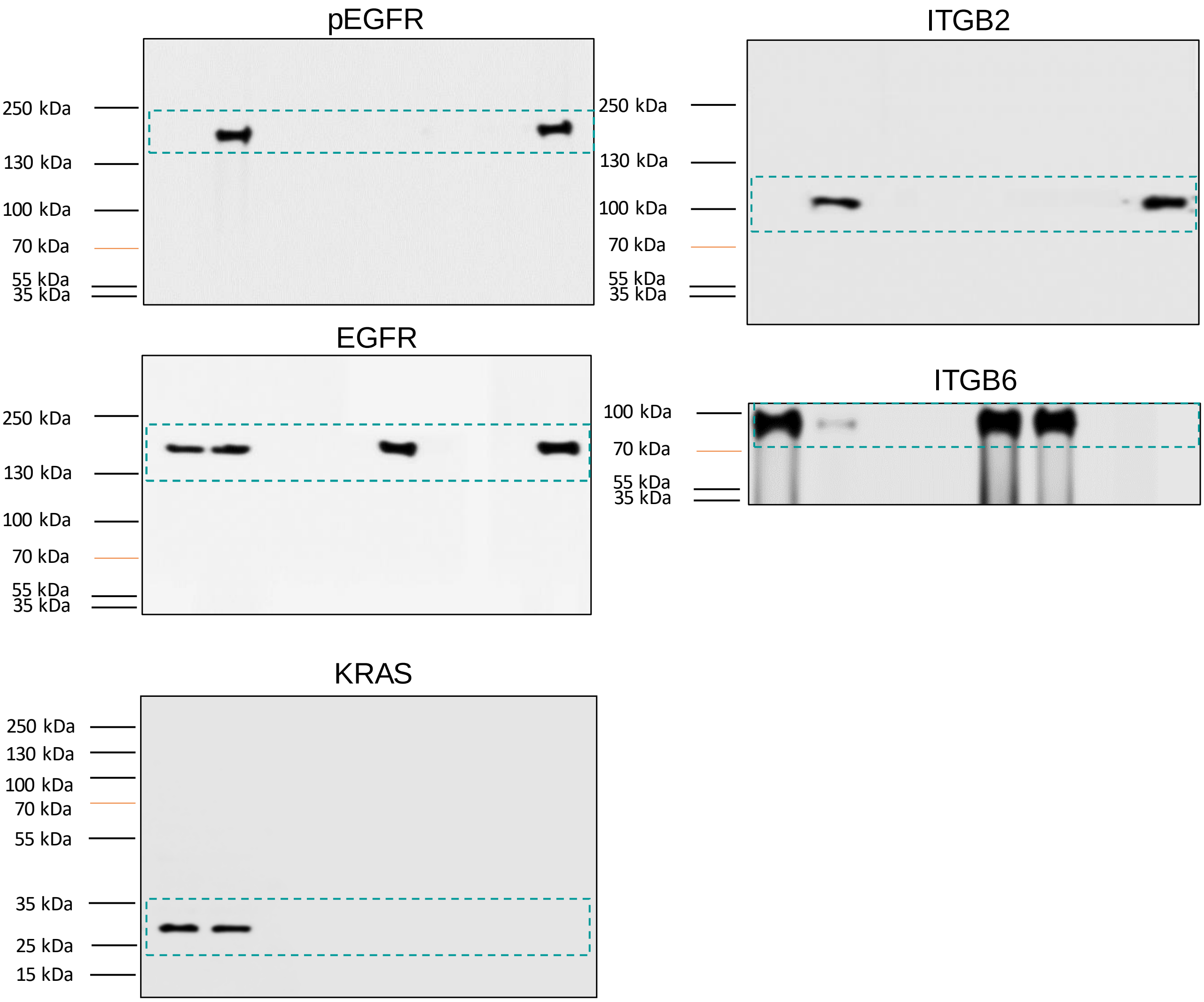

Fig 6B. WB of total protein extracts of NCI-H196 cells transfected with Ctrl or ITGB6, and immunoprecipitated using either IgG control or ITGB2-specific antibodies

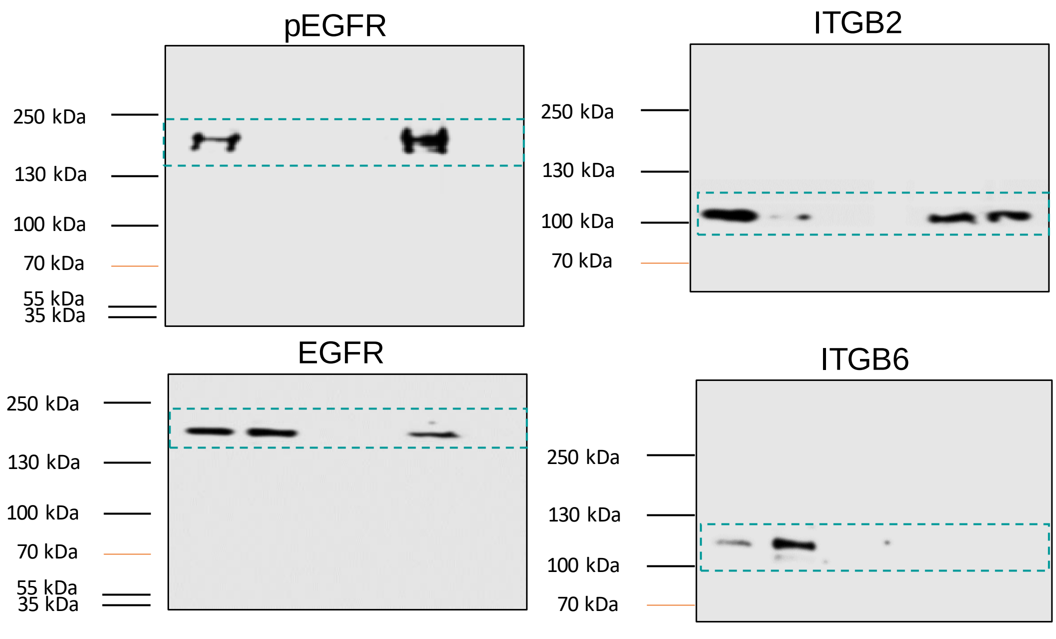

Fig 6C. WB of protein extracts of NCI-H196 cells transfected with *siITGB2*, *siGAL3* or *siKRAS*, and immunoprecipitated using either IgG control or EGFR-specific antibodies

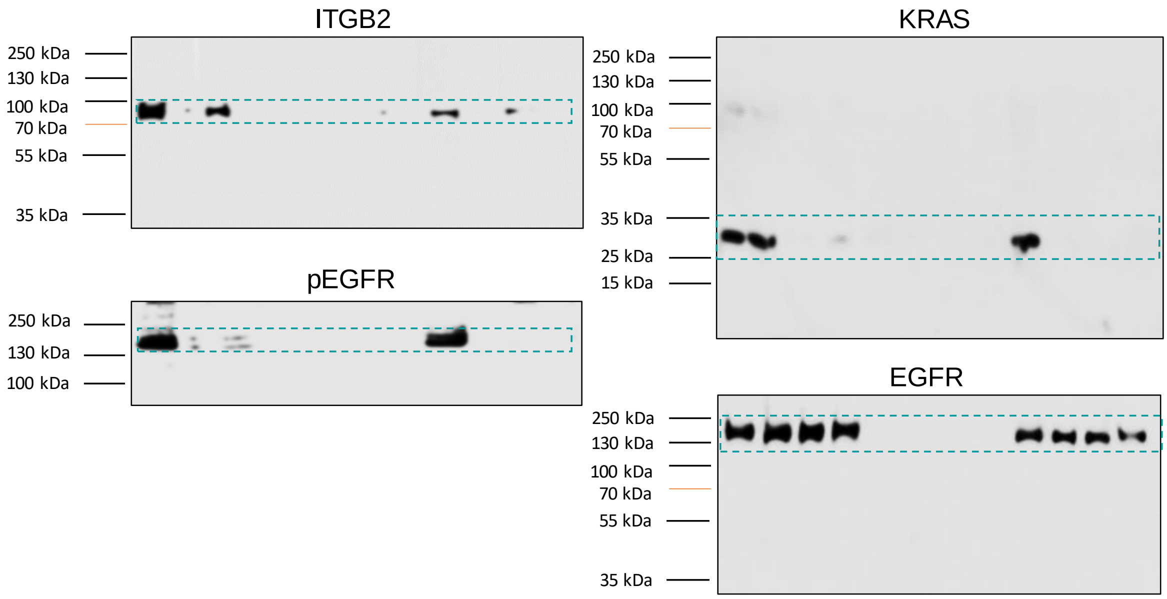

Fig 6D. WB of protein extracts of A549 cells transfected with ITGB2 or mutITGB2, and *siGAL3*, and immunoprecipitated using KRAS-specific antibody or RAF-RBD coated beads

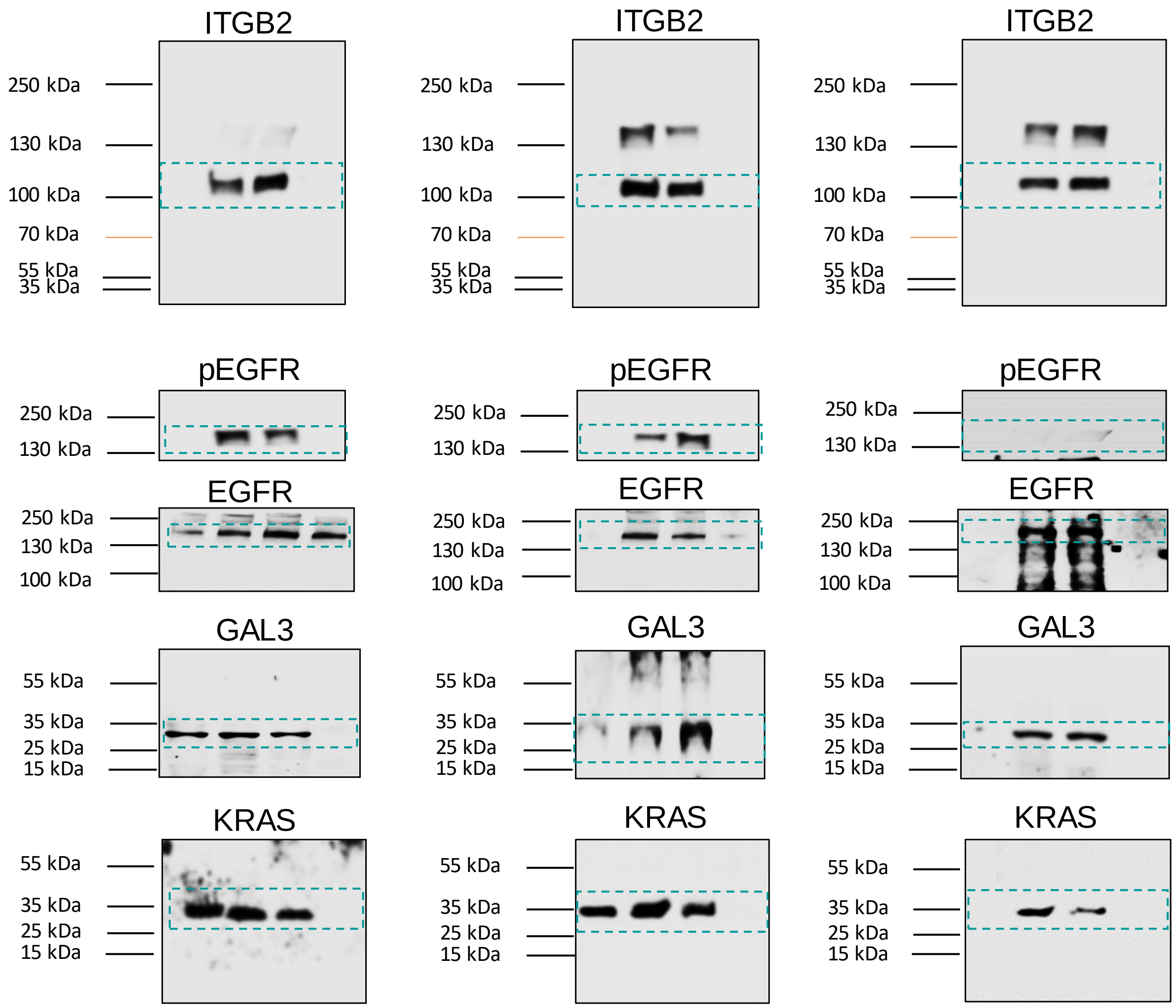

Fig 7F. WB of total protein extracts of EVs from A549 cells transfected with ITGB2 or mutITGB2

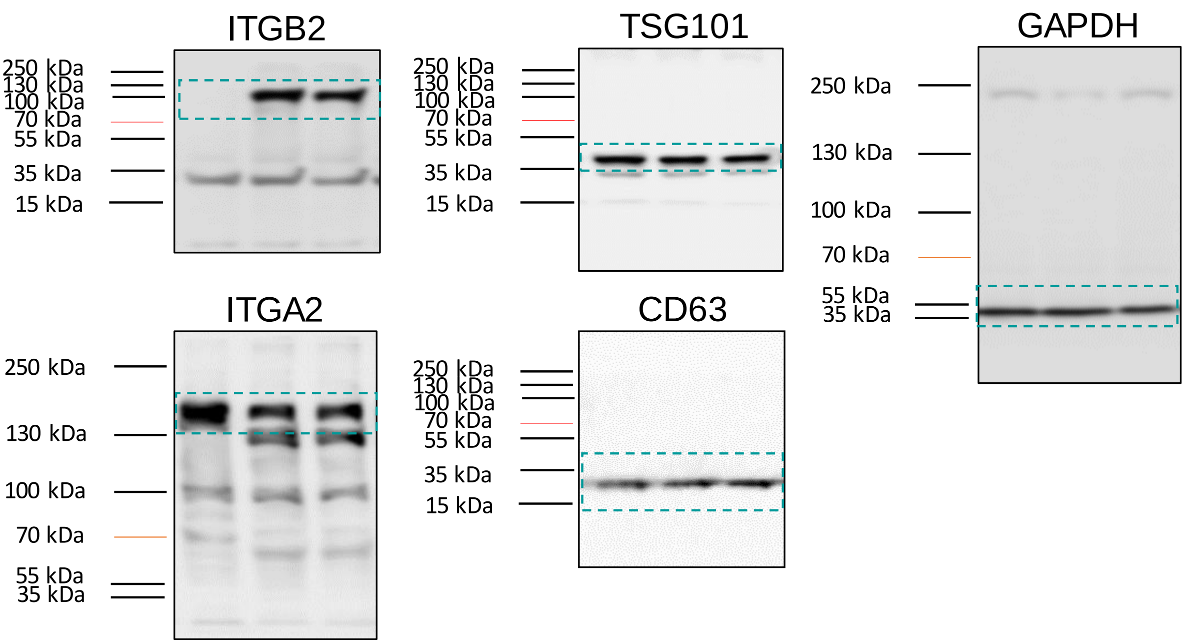

Fig 7H. WB of total protein extracts of hPCLS incubated with EVs from A549 cells previously transfected with ITGB2 or mutITGB2, alone or in combination with binase

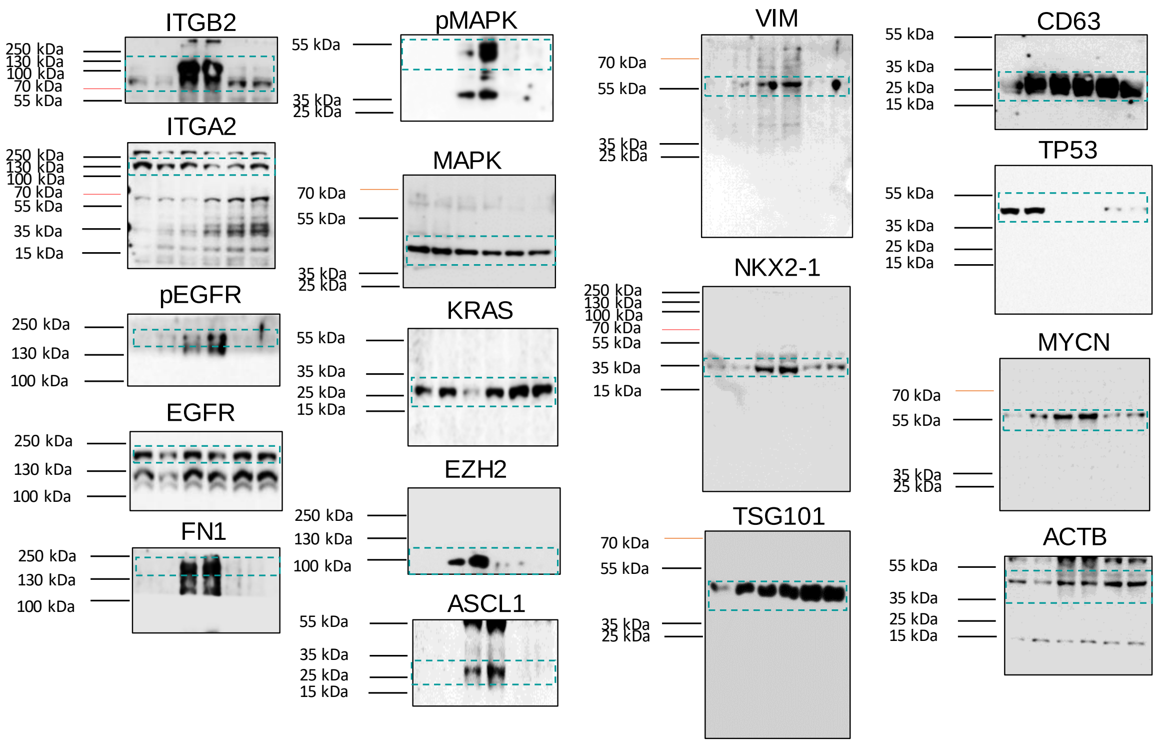

Fig 8A. WB of total protein extracts of EVs from NCI-H196 cells transfected with control, *siCtrl*, *siITGB2*, or treated with binase

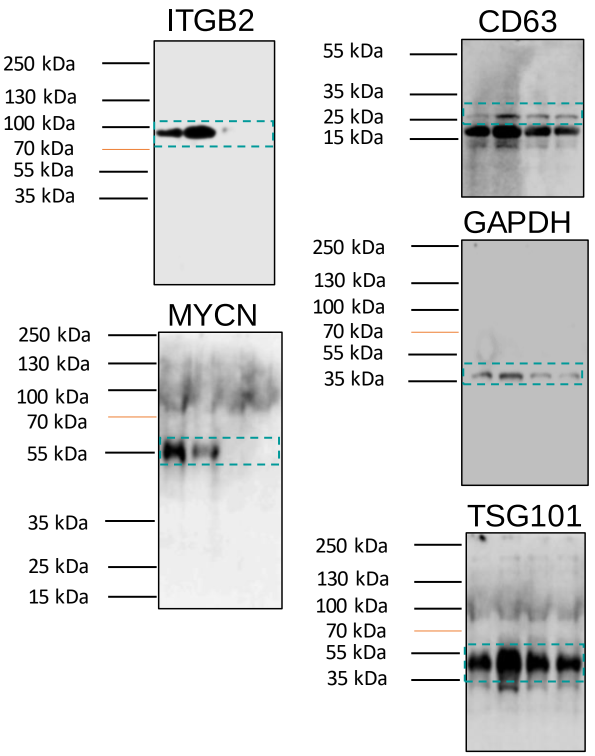

Fig 8D. WB of total protein extracts of hPCLS incubated with EVs from NCI-H196 cells previously transfected with *siCtrl* or *siITGB2*, alone or in combination with binase

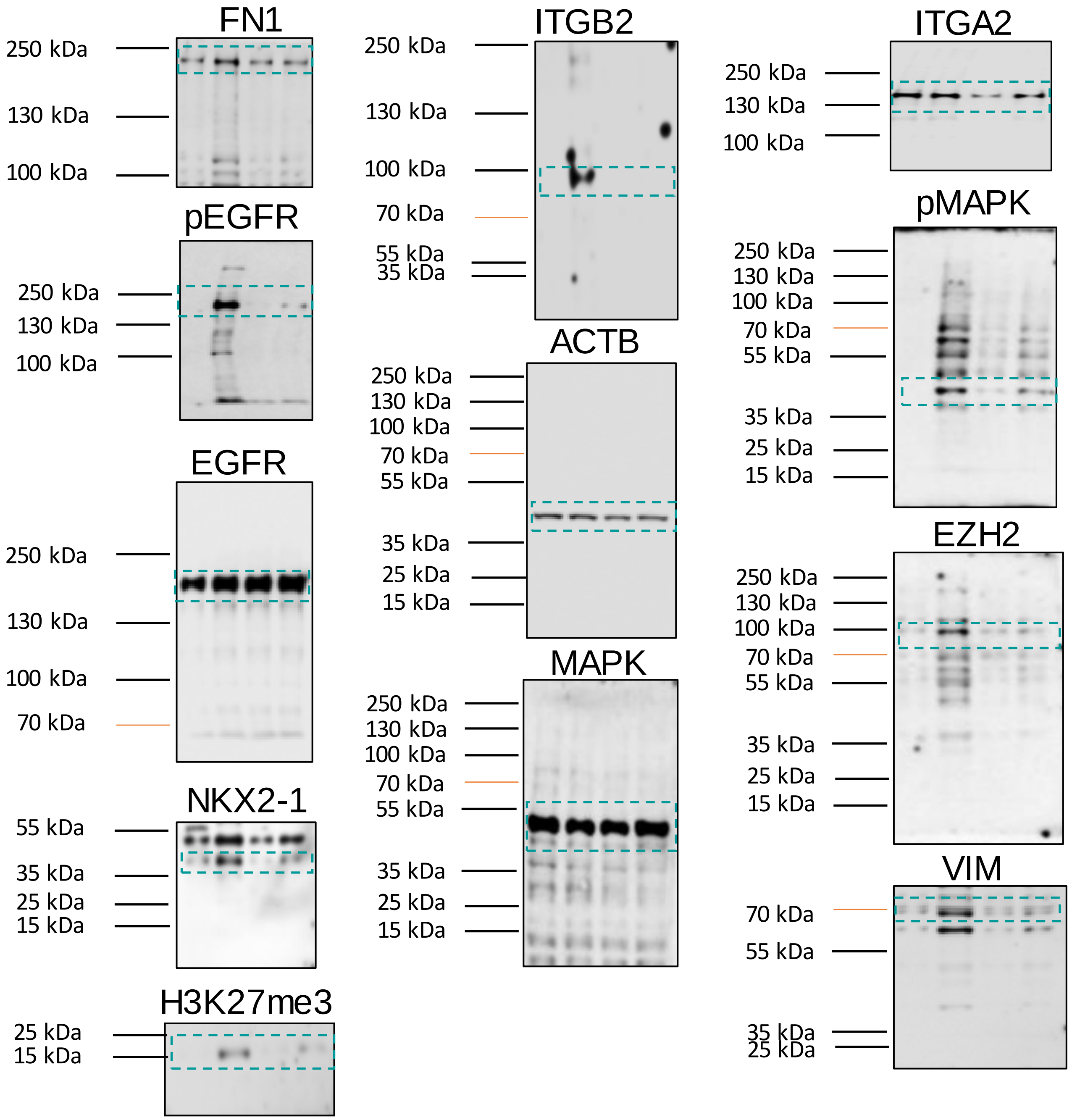

Fig S5B. WB of total protein extracts of A549 cells transfected with ITGB2 or mutITGB2

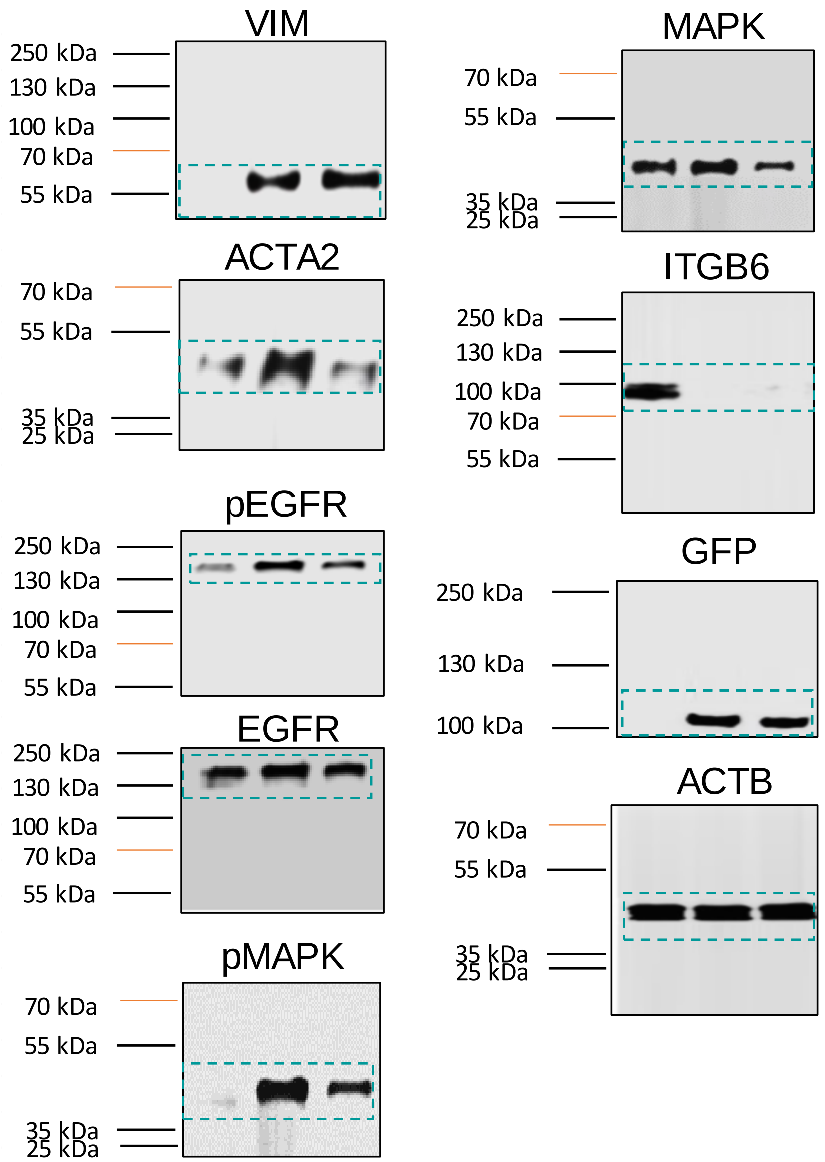

Fig S5C. WB of total protein extracts from NCI-H196 cells transfected with Ctrl, ITGB2- or GAL3-specific siRNAs

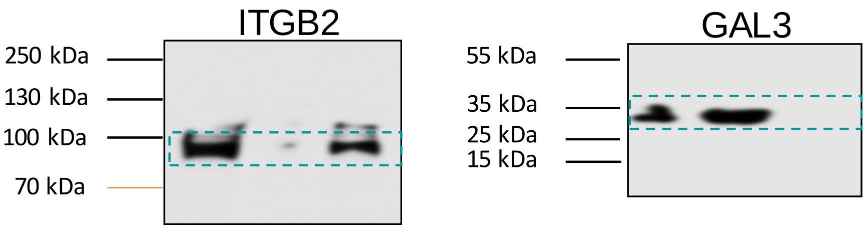

Fig S10A. WB of total protein extracts of NCI-H82 and NCI-H196 cells treated with Placebo or with binase

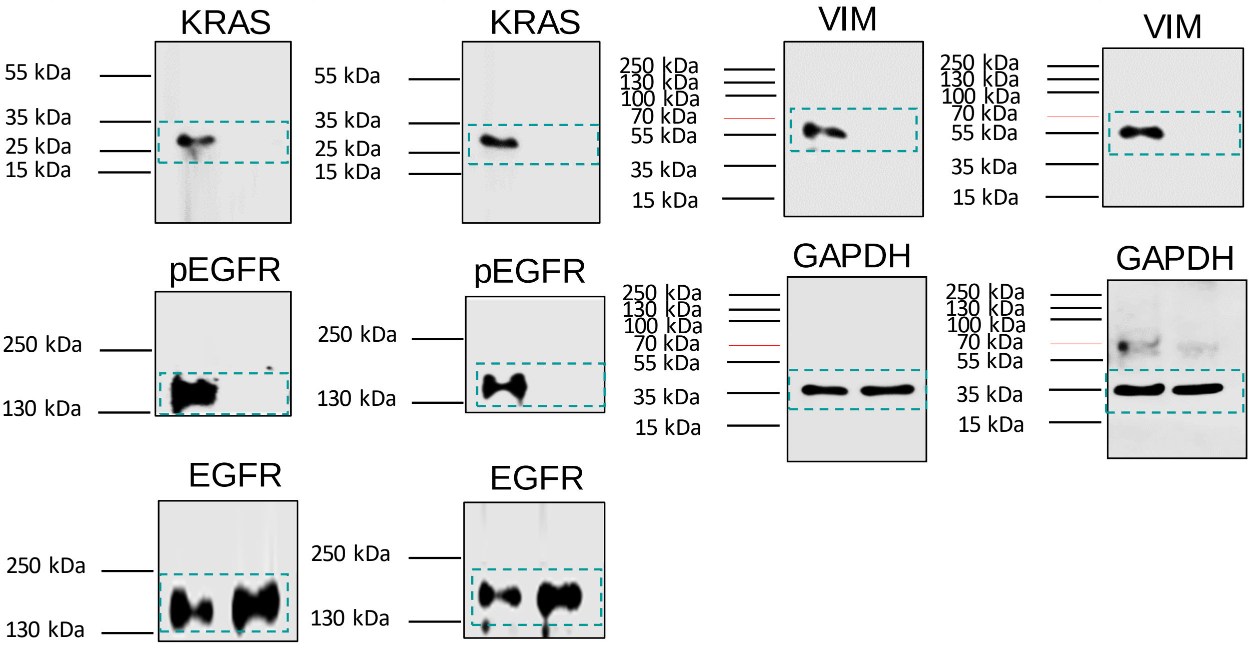

Fig S10E. WB of CRISPR-induced KO of *ITGB2*

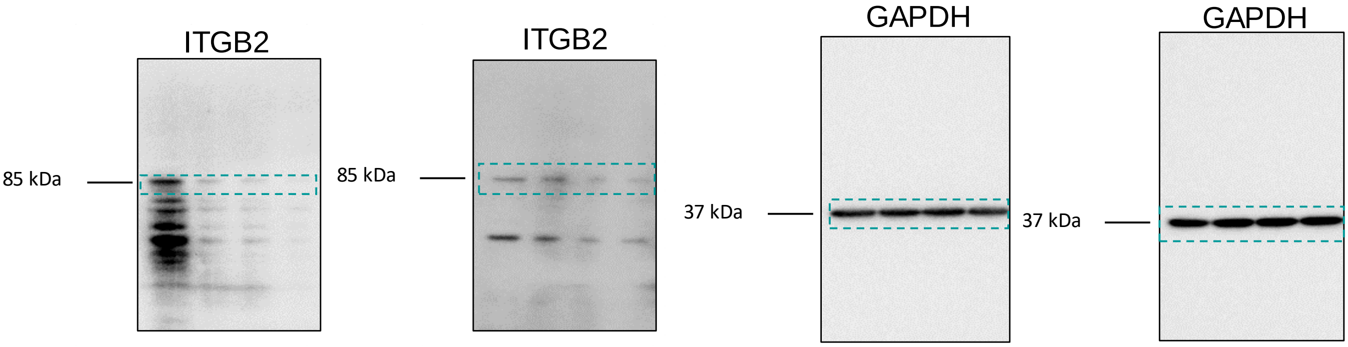

Fig S11A. WB of SCLC cells treated with erlotinib alone or in combination with *siITGB2*

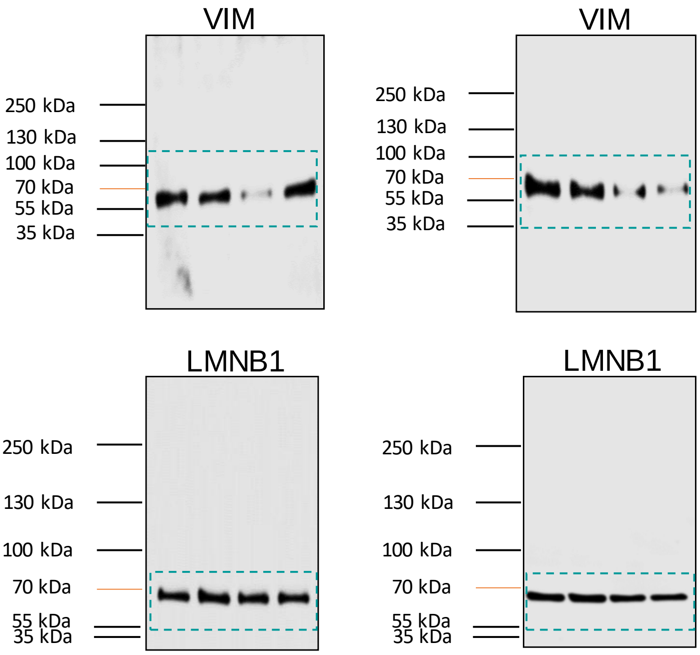

Supplement: Supplementary file 3 — Supplementary source data 2. [file thnov13p2384s3.pdf]
